# Metal-free domino amination-Knoevenagel condensation approach to access new coumarins as potent nanomolar inhibitors of VEGFR-2 and EGFR

**DOI:** 10.1080/17518253.2021.1981462

**Published:** 2021-09-24

**Authors:** Essam M. Eliwa, Marcel Frese, Ahmed H. Halawa, Maha M. Soltan, Larissa V. Ponomareva, Jon S. Thorson, Khaled A. Shaaban, Mohamed Shaaban, Ahmed M. El-Agrody, Norbert Sewald

**Affiliations:** a Organic and Bioorganic Chemistry, Faculty of Chemistry, Bielefeld University, Bielefeld, Germany; b Chemistry Department, Faculty of Science, Al-Azhar University, Nasr City-Cairo, Egypt; c Biology Unit, Central Laboratory for Pharmaceutical and Drug Industries Research Division, Chemistry of Medicinal Plants Department, Pharmaceutical and Drug Industries Research Division, National Research Centre Cairo, Egypt; d Center for Pharmaceutical Research and Innovation, University of Kentucky, Lexington, USA; e Department of Pharmaceutical Sciences, College of Pharmacy, University of Kentucky, Lexington, USA; f Chemistry of Natural Compounds Department, Pharmaceutical and Drug Industries Research Division, National Research Centre, Dokki-Cairo, Egypt

**Keywords:** Coumarin, metal-free domino reaction, C–N coupling, Knoevenagel condensation, VEGFR-2, EGFR

## Abstract

A metal-free, atom-economy and simple work-up domino amination-Knoevenagel condensation approach to construct new coumarin analogous (**4a-f** and **8a-e**) was described. Further, new formyl (**5a,d-f**) and nitro (**9a,d-f**) coumarin derivatives were synthesized via C-N coupling reaction of various cyclic secondary amines and 4-chloro-3-(formyl-/nitro)coumarins (**1a,c**), respectively. The confirmed compounds were screened for their *in vitro* anti-proliferative activity against KB-3-1, A549 and PC3 human cancer cell lines using resazurin cellular-based assay. Among them, coumarin derivatives **4e** and **8e** displayed the best anti-cervical cancer potency (KB-3-1) with IC_50_ values of 15.5 ± 3.54 and 21 ± 4.24 μM, respectively. Also, **4e** showed the most promising cytotoxicity toward A549 with IC_50_ value of 12.94 ± 1.51 μM. As well, **9d** presented a more significant impact of potency against PC3 with IC_50_ 7.31 ± 0.48 μM. Moreover, **8d** manifested selectivity against PC3 (IC_50_ = 20.16 ± 0.07 μM), while **8e** was selective toward KB-3-1 cell line (IC_50_ = 21 ± 4.24 μM). Matching with docking profile, the enzymatic assay divulged that **8e** is a dual potent single-digit nanomolar inhibitor of VEGFR-2 and EGFR with IC_50_ values of 24.67 nM and 31.6 nM that were almost equipotent to sorafenib (31.08 nM) and erlotinib (26.79 nM), respectively.

## Graphical Abstract

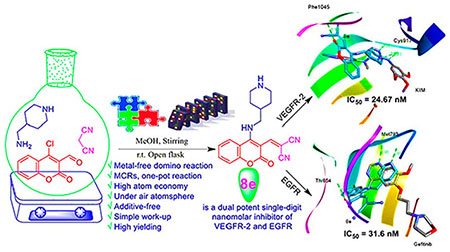

## 1. Introduction

Cancer remains one of the most daunting diseases to treat, thus, the development of new antitumor agents is still a very critical research domain. Amongst the attractive therapeutic targets for cancer, protein tyrosine kinases (PTKs) that regulate the biological potency of proteins by phosphorylation process that play a crucial role in signal transduction mechanisms by which inter-cellular signals regulate significance intra-cellular functions such as ion transport, cellular proliferation, differentiation, angiogenesis, and hormone responses. Among them, the epidermal growth factor receptor (EGFR) and vascular endothelial growth factor receptor (VEGFR) are receptor protein tyrosine kinases (RPTKs) that overexpressed or mutated in several tumors due to a mutation of a normal gene to an oncogene [1]. EGFR (a dimer of HER-1 and HER-2) and VEGFR-2 (KDR, a type of VEGFRs) are pro-angiogenic growth factor receptors that induce angiogenesis process in order to establish new blood vessels that have an important role in tumor growth and metastasis; therefore, they are attractive therapy targets and dominant strategy for the treatment of cancer [1–6].

VEGFR-2 type II inhibitors shared essential pharmacophoric features encompassing: (i) terminal heteroaromatic ring that occupies the ATP binding pocket (hinge region) via H-bond with cysteine acid (like Cys917); (ii) oxygen (N or S) linker that occupies the gatekeeper region between the hinge and DFG domains; (iii) urea or amide moiety spacer as H-bond acceptor–donor pair (HBA–HBD) that binds to DFG motif via H-bonds with glutamic (Glu833) and aspartic (Asp1044) acids; (iv) terminal lipophilic group that occupies the allosteric hydrophobic pocket (DFG-out) via hydrophobic interactions [7–11]. The well-defined EGFR inhibitors consists of: (i) central hetero aromatic unit that fits the adenine binding site through H-bond with methionine amino acid residue (Met793); (ii) hydrophobic head is a phenyl binding group with various hydrophobic substituents to interact with the hydrophobic region I; (iii) nitrogen spacer to link the hinge-binding central moiety with the terminal fragment that occupies the hydrophobic region I; (iv) hydrophobic tail is directly linked to the central heteroaromatic core and induced fit to the hydrophobic region II [12].

Coumarins are versatile oxygen-containing heterocyclic molecules in the medicinal chemistry domain due to their aspirational biological applications [13,14]. Particularly, they have anti-coagulant, anti-oxidant [15], anti-microbial, anti-viral [16–18], anti-cancer [19–24], anti-diabetic, analgesic, anti-neurodegenerative, and anti-inflammatory properties [17,25]. Interestingly, several coumarin derivatives obtained via Knoevenagel condensation reactions have been proved to be potent enzymes inhibitors [26–28]. Also, Knoevenagel reaction products are significant key intermediates for the synthesis of many marketed drugs such as atorvastatin (Lipitor®) and pioglitazone (Actos®) [29].

The recently developed transition-metal-catalyzed reactions have been confirmed as useful protocols for the synthesis of heterocyclic compounds as well. However, most of these approaches suffer from some drawbacks encompassing complex procedures (e.g. high expense, moisture sensitivity and toxicity nature of many of these metal catalysts and ligands). Consequently, studies of eco-friendly organic transformations using commercially accessible and cost-efficient reagents have also been recently extended [30–33]. Multi-component transformation domino reactions are a formidable and significant method in the organic synthesis domain due to their benefits to our environment and natural resources [34].

Synthesis and investigation of heterocyclic/aryl amines still cornerstone research area in medicinal chemistry and its neighboring disciplines. In particular, due to their high prospective to demonstrate potent biological activity and their application as lead compounds in drug design, as well as they are vital building blocks of various organic compounds [30]. The most effective methodology for the construction of heterocyclic/aryl amines is the C–N cross-coupling reaction between heterocyclic/aryl halides and primary or secondary amines via the Buchwald–Hartwig reaction using high-expensive phosphine-ligated palladium precatalysts [35]. As a result, a daunting application in the pharmaceutical industry. Hence, the development of metal-free C–N coupling reactions is a surrogate trend and hold a prominent position [30,31].

Spur by the aforementioned facts and our persistent research efforts to explore highly effective and convenient synthetic routes for the synthesis of bioactive molecules [22,24], we present in this research paper an efficient cascade amination-Knoevenagel strategy for the construction of new coumarin derivatives under metal-free conditions. Their anti-proliferative activity was evaluated against KB-3-1, A549 and PC3 cell lines using resazurin cellular assay. Moreover, we highlighted the *in silico* computational predictions including physicochemical features and biological targets of the active compounds. Furthermore, molecular docking of the most robust compounds **4e**, **8e** and **9d** as potential inhibitors of vascular endothelial growth factor receptor (VEGFR-2) and epidermal growth factor receptor (EGFR) biomolecular targets was performed. Experimentally, an enzymatic assay for the promising dual inhibitors of VEGFR-2 and EGFR was investigated as well, delivering very potent inhibitory activities.

## 2. Results and discussion

### 2.1. Chemistry

In the preliminary investigation, we tried a model reaction of 4-chloro-3-formylcoumarin (**1a**) as an example of α,β-unsaturated-β-haloaldehydes [36,37], acidic CH_2_ called methyl cyanoacetate (**2a**), and a cyclic secondary amine named pyrrolidine (**3a**) in ethanol (EtOH) at room temperature. As a result, compound **4a** was generated with 65% yield in 40 min (Table 1, entry 1). As previously reported [38,39], in nucleophilic aromatic substitution (S_N_Ar)-Knoevenagel condensation domino reaction, the amine plays twofold role as a nucleophilic and base, hence, the produced HCl was neutralized with an excess of amine or additional base to perform the reaction in smoothly way. Consequently, the reactants ratio of **1a** (1 equiv), **2a** (1 equiv), and **3a** (2.5 equiv) was used as an optimized reaction condition (Table 1). Also, the yield was further investigated by utilizing different solvents such as methanol (MeOH), dichloromethane (DCM), and water. We observed that MeOH was premium for the reaction and afforded **4a** in the best yield (Table 1, entry 3). All the optimization trials were carried out under oxygen and air atmospheres (open flask).

**Scheme 1.**
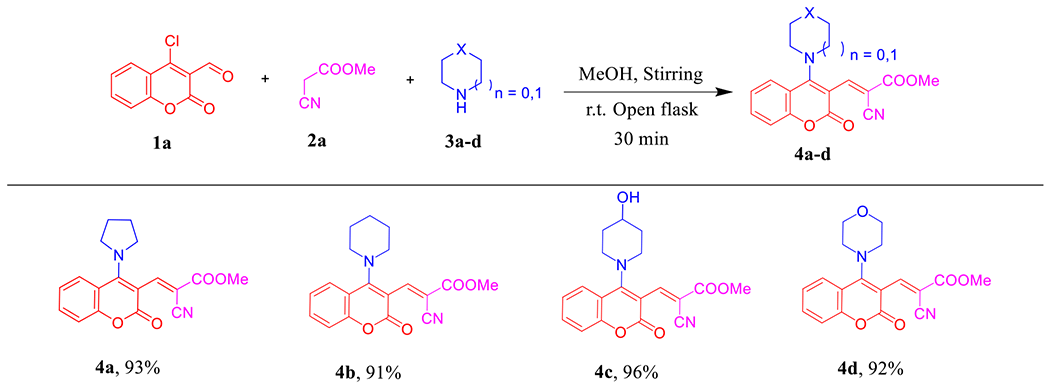
Scope of various cyclic secondary amines for the synthesis of **4a–d**. *Reagents and conditions:* **1a** (1.0 mmol, 1.0 equiv), **2a** (1.0 mmol, 1.0 equiv), and **3a–d** (2.5 mmol, 2.5 equiv) in MeOH (2 mL) using open flask at room temperature under stirring conditions.

Once the optimal reaction conditions had been established (Table 1, entry 3), we explored the scope and generality of the tandem approach (Scheme 1). A variety of cyclic secondary amines [piperidine (**3b**), 4-hydroxypiperidine (**3c**), and morpholine (**3d**)] were used and to our delight, all the corresponding desired products **4b-d** were obtained in excellent yields (91–96%) and purified by simple recrystallization/washing with hot MeOH where we did not need to perform the column chromatography work-up as in the previously reported methods [38,39].

**Scheme 2.**
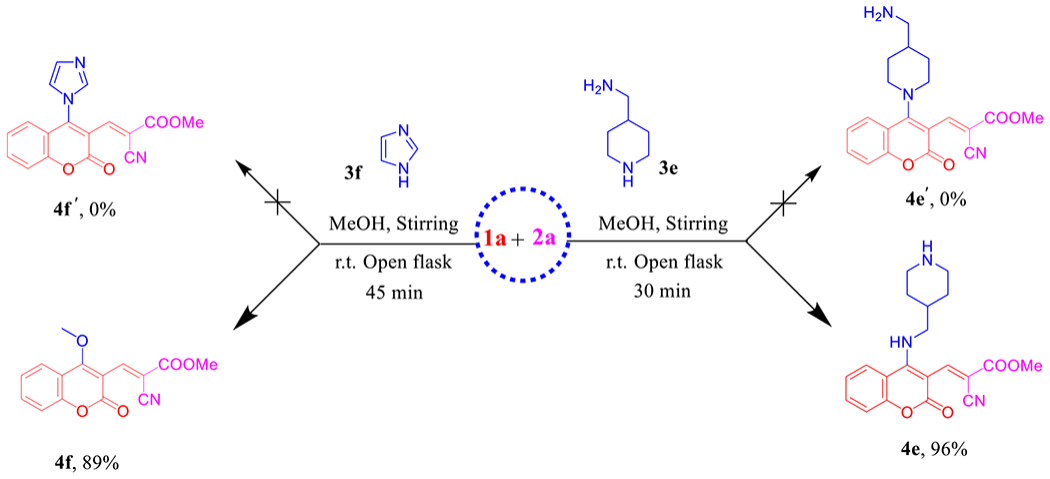
Synthesis of **4e** and **4f**.

In the case of the bifunctional amine that called; 4-(aminomethyl)piperidine (4-AMP, **3e**), we got **4e** as an exclusive and regiospecific product [40–43] in high yield instead of **4e**′ (Scheme 2). Our interpretation based on the concept; nucleophilicity is much more sensitive to steric effects than basicity, thus in this case primary amine (−CH_2_NH_2_) is more nucleophilic than the secondary one (−CH_2_NHCH_2_−) and consider as the preference orientation. The obtained secondary amine **4e** can be easily identified from the ^1^H NMR spectrum by the characteristic ^1^H chemical shift of aromatic triplet NH at *δ* 8.76 ppm (see Figure S13, Supporting Information). Also, it seems worthwhile to point out that **4e** among this series showed [M + H]^+^ adduct ion in (+)-ESI-MS, while all the others exhibited [M + Na]^+^ molecular ion peaks.

**Scheme 3.**
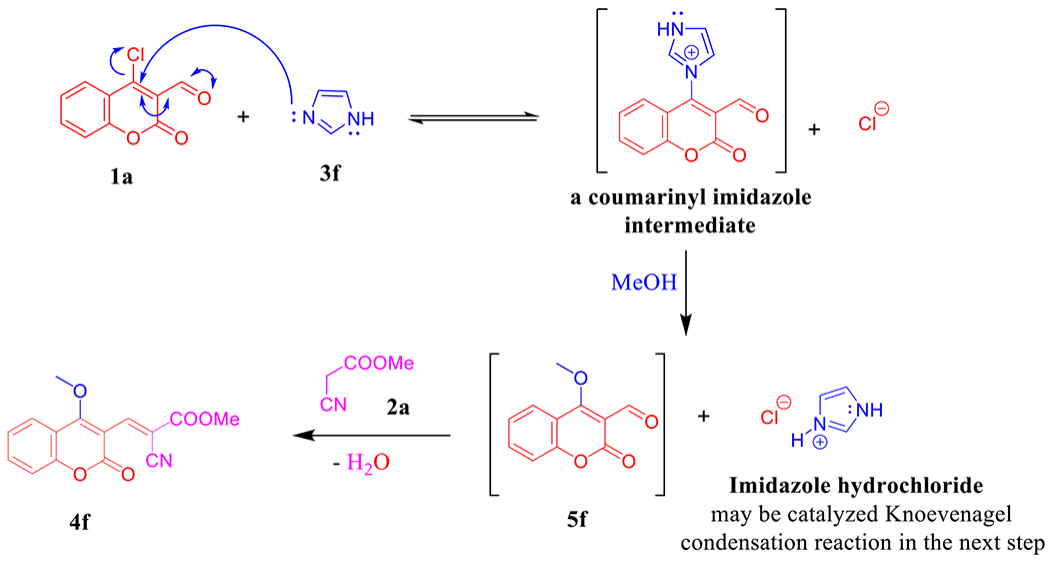
Plausible reaction mechanism of the imidazole-catalyzed C–O bond formation in **4f**.

Under the aforementioned conditions, our reconnaissance was expanded to utilize imidazole (**3f**) instead of the above-mentioned amines in an attempt to get the desirable product **4f**′, astonishingly, 4-methoxy coumarin derivative **4f** was isolated in 89% yield (Scheme 2). Unambiguously, imidazole dealing with the reaction as a nucleophilic organocatalyst (covalent catalyst) by forming a covalent bond with **1a** [44] to produce a reactive intermediate because the positively charged nitrogen makes imidazole a very good leaving group and subsequently promoted the MeOH to attack the intermediate. Hence, imidazole hydrochloride may be catalyzed Knoevenagel condensation reaction in the next step to furnish **4f** (Scheme 3). NMR data of **4f** manifested the new methoxy ^1^H resonance at *δ* 4.13 and the corresponding ^13^C chemical shift at *δ* 63.8 ppm. The (+)-ESI-MS analysis of compound **4f** proved its molecular weight as 285 Da, where it showed the molecular ion peak at *m/z* 308 [M + Na]^+^ (see Supporting Information, Figs. S16–S18).

**Scheme 4.**
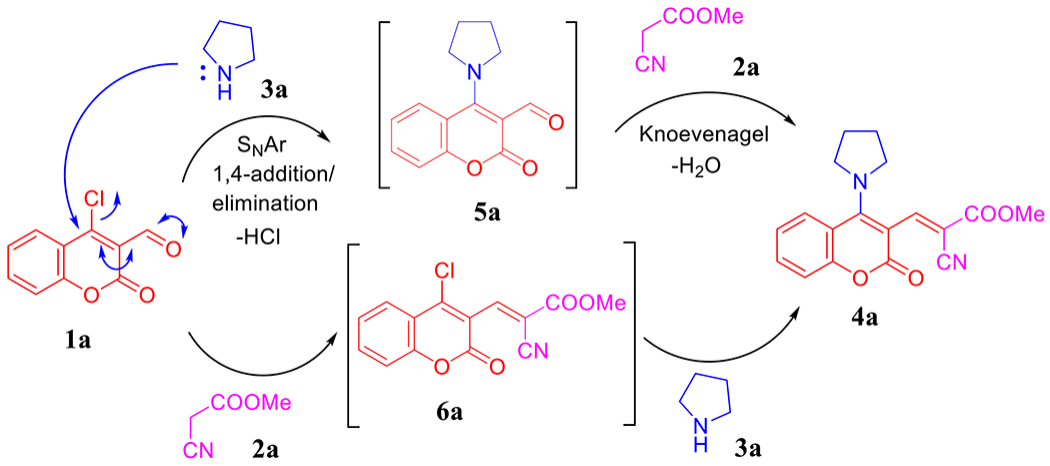
Plausible reaction mechanism.

Having in mind the previous studies [38,45], we propose that the cascade one-pot reaction mechanism could be implemented via two routes as depicted in Scheme 4. First, **1a** underwent S_N_Ar reaction with pyrrolidine (**3a**) through 1,4-addition/elimination pathway to afford **5a** that condensed with methyl cyanoacetate (**2a**) to generate **4a**. As an alternative strategy, Knoevenagel product **6a** was formed by pyrrolidine-catalyzed condensation reaction between **1a** and **2a**. Hence, subsequent S_N_Ar reaction between **6a** and **3a** furnished **4a**.

**Scheme 5.**
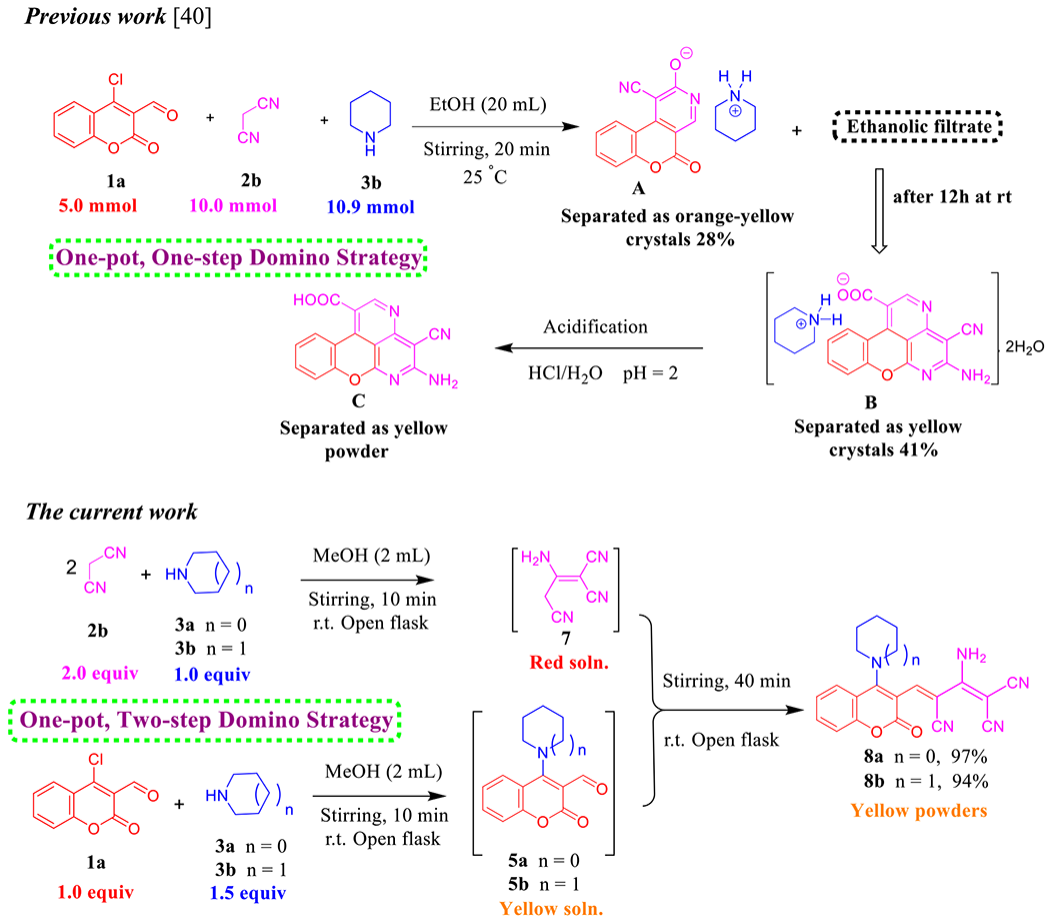
Previous work by Angelova group and synthesis of **8a,b**.

Besides methyl cyanoacetate (**2a**), ethyl nitroacetate or cyanoacetic acid were used as a source of active methylene in this reaction with pyrrolidine (**3a**) and our substrate **1a**. Under the optimized conditions, we obtained a mixture of products that may be involved amine salt and S_N_Ar product **5a**. Instead, we worked on expanding the application of this approach to involve malononitrile (**2b**) as a suitable starting material for the Knoevenagel condensation. Hereupon, we used the above-mentioned reaction conditions albeit with one-pot, two-step domino strategy. First, base (1.0 equiv, **3a**/**3b**)-catalyzed dimerization of malononitrile (2.0 equiv, **2b**) into non-isolated 2-amino-1,1,3-tricyanopropene (**7**) [ 46] that has drawn tremendous attention of interest due to its comprehensive implementations in the synthesis of heterocyclic compounds that manifest diverse biological and pharmaceutical properties [47]. At the same time, in another conical flask, suspension solution of 4-(pyrrolidin-1-yl)-3-formylcoumarin (**5a**)/4-(piperidin-1-yl)-3-formylcoumarin (**5b**) in MeOH (2 mL) were prepared from direct amination of **1a** (1.0 equiv) and **3a/3b** (1.5 equiv). Next, sequential addition of **7** to **5a**/**5b** afforded the corresponding products **8a**/**8b** in excellent isolated yields (Scheme 5).

**Figure 1.**
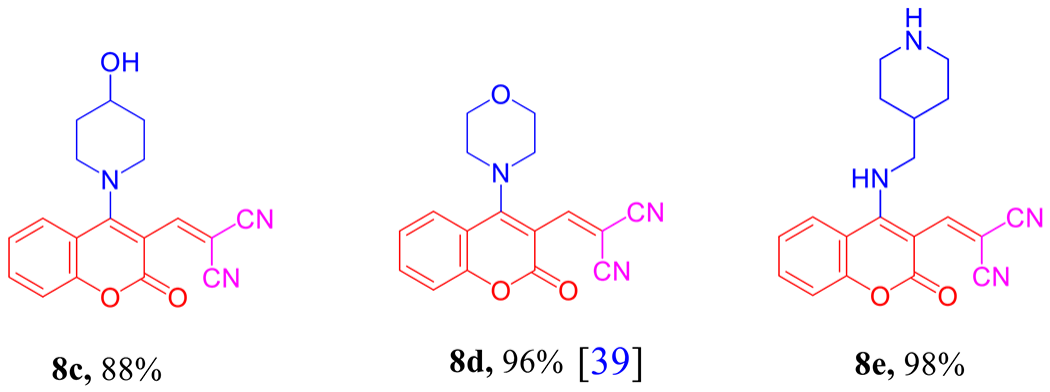
Chemical structures of the prepared methylene malononitrile derivatives **8c–e**.

As delineated in Scheme 5, in contrast to the published research paper by Angelova et al. [46] who described the synthesis of aminium salt **A** and piperidinium 5-amino-4-cyanochromeno[4,3,2-*de*]-1,6-naphthyridine-1-carboxylate dihydrate (**B**) that converted to the acidic form **C** via one-pot, one-step cascade reaction of starting materials **1a**, **2b**, and **3b** as developed route to generate polyfunctionality substituted heterocyclic compounds with anticipated biological performance. Herein, our chemistry proposal was aimed to synthesize buta-1,3-diene-1,1,3-tricarbonitrile derivatives **8a,b** with potential anti-proliferative activity and act as a key intermediate to construct privileged molecules. Therefore, we developed a striped pathway to achieve our target but, unfortunately, we failed to expand the scope of this methodology to include other bases such as 4-hydroxypiperidine (**3c**), morpholine (**3d**), and 4-AMP (**3e**), where the corresponding methylene malononitrile derivatives **8c–e** (**8d** [39]) were produced in high yields 88–98% (Figure 1). We postulate this contradiction is attributed to the difference in basicity power between **3a,b** and **3c–e**.

**Scheme 6.**
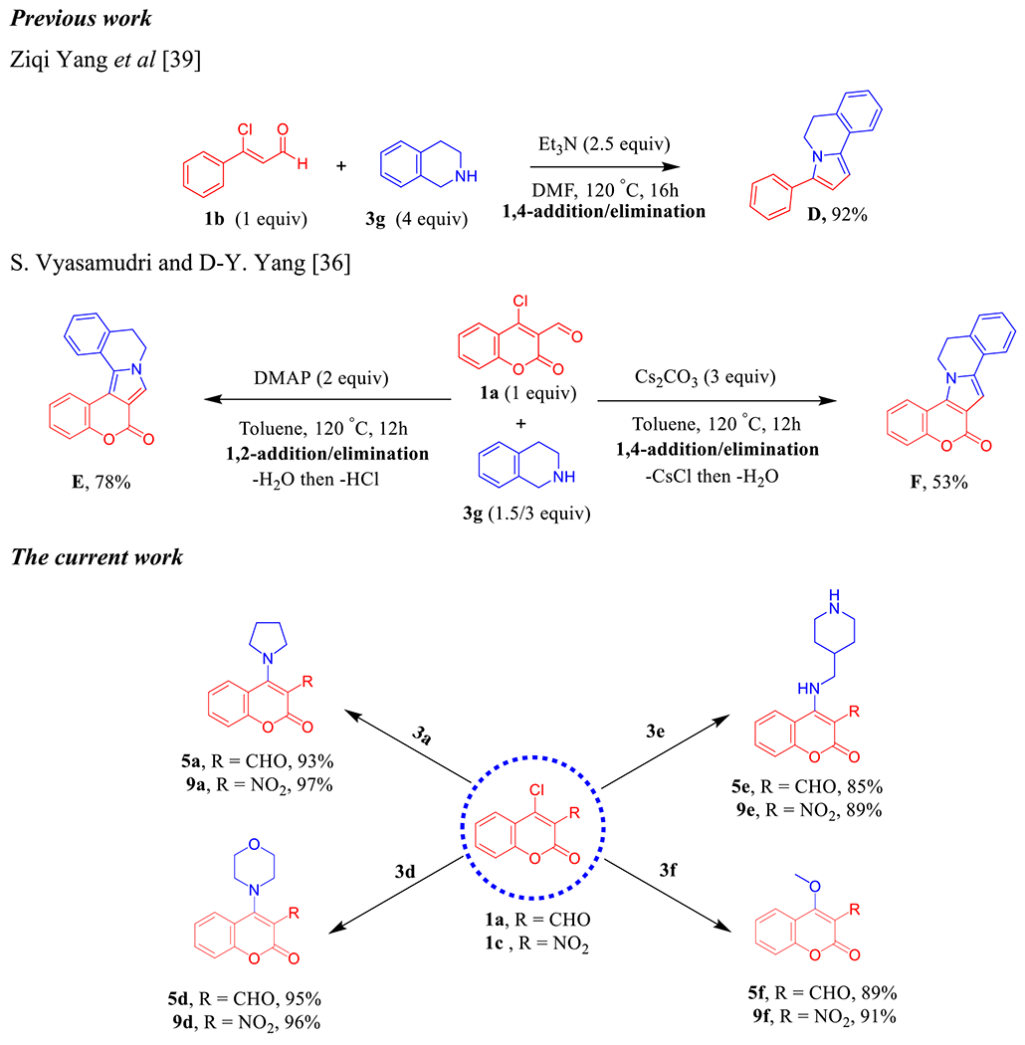
Previous work by Yang and Vyasamudri groups as well as scope of various cyclic secondary amines for the synthesis of **5a, d–f** and **9a,d–f**. *Reagents and conditions:* **1a,c** (1.0 mmol, 1.0 equiv), **3a,d–f** (1.0 mmol, 1.0 equiv), and Et_3_N (1 mmol, 1 equiv) in MeOH (2 mL) using open flask at room temperature under stirring conditions, R = CHO, 15 min, R= NO_2_, 5 min.

Next, we turned our concern to confirm the amination occurs via 1,4-addition/elimination S_N_Ar reaction as a part of the mechanistic study (Scheme 4) and control experiments. Earlier study by Yang et al. [48] has been showed that the reaction between 3-chloro-3-phenylacrylaldehyde **1b** and 1,2,3,4-tetrahydroisoquinoline (THIQ, **3g**) exhibited predominant product **D** through Et_3_N-catalyzed 1,4-addition/elimination followed by intramolecular cyclization process (Scheme 6). In 2019, Vyasamudri and Yang [45] have reported that condensation of 4-chloro-3-formylcoumarin (**1a**) and THIQ (**3g**) using 4-dimethylaminopyridine (DMAP) as an organic base afforded **E** in good yield 78% and the reaction goes forward with 1,2-addition/elimination then cyclization process through removing of HCl. On the other hand, when Cs_2_CO_3_ was utilized as the base, the reaction proceeds via 1,4-addition/elimination then annulation fashion to furnish **F** with moderate yield 53% (Scheme 6). In our manner, the amination process of **1a** and **3a,d,e** passed through Et_3_N-promoted1,4-addition/elimination at room temperature in the open flask using MeOH to supply **5a,d,e** with high yields and the subsequent intramolecular cyclization did not take place. We likewise sought to expand this fashion to include 4-chloro-3-nitrocoumarin (**1c**) with **3a,d,e**. The desired corresponding amination products **9a,d,e** were isolated in excellent yields and shorter time. As produced in the above series, we got the corresponding methoxy coumarin analogs **5f** and **9f** when imidazole (**3f**) was utilized as a reactant base (Scheme 6).

### 2.2. Biological evaluation

#### 2.2.1. In vitro anti-proliferative activity

**Figure 2.**
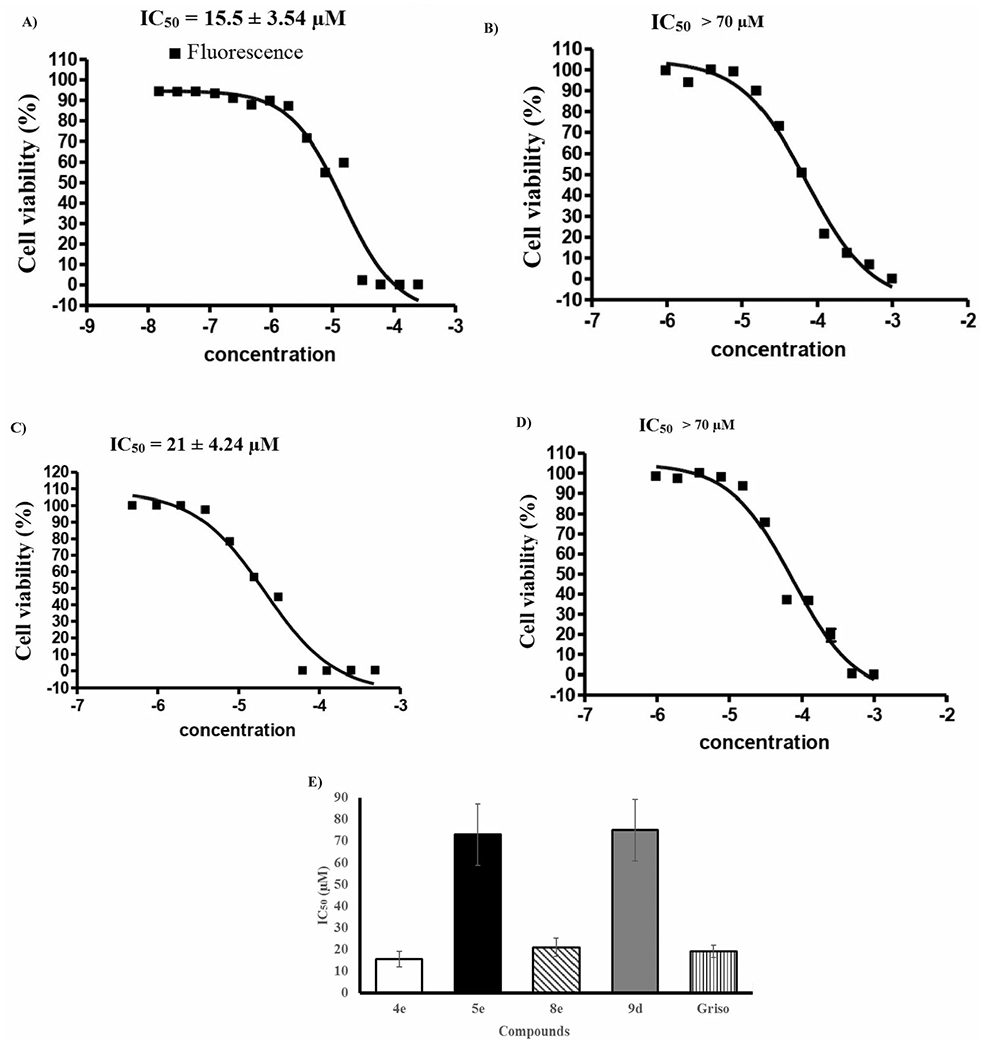
Sigmoidal dose–response against KB-3-1 cell line; (A) **4e**, (B) **5e**, (C) **8e**, (D) **9d**, and (E) graph show the IC_50_ values of the tested compounds **4e**, **5e**, **8e**, **9d**, and (+)-griseofulvin as a positive control.

Anti-cervical cancer activity of all the synthesized compounds was evaluated for their *in vitro* cytotoxicity toward KB-3-1 cell line by resazurin-based assay [49] using (+)-griseofulvin as a positive control. The results were expressed as IC_50_ values that outlined in Table 2, Figure 2, and the values are an average ± SD of at least two separate experiments. Table 2 and Figure 2 show that the domino amination-Knoevenagel product **4e** that containing 4-AMP core displayed superior potency (IC_50_ = 15.5 ± 3.54 μM) than (+)-griseofulvin (IC_50_ = 19 ± 2.83 μM) while **8e** is the closer one (IC_50_ = 21 ± 4.24 μM). The amination products **5e** and **9d** showed moderately convergent activity with IC_50_ > 70 μM. On the other hand, compounds **4c,d**, **5d**, **8d**, and **9a** indicated low activity with IC_50_ values over than 100 μM, whilst molecules **4a,b**, **4f**, **5a,f**, **8a–c**, and **9e,f** showed no activity against KB-3-1. The results manifested that the presence of 4-AMP fragment was benefit for the anti-cervical cancer activity.

**Figure 3.**
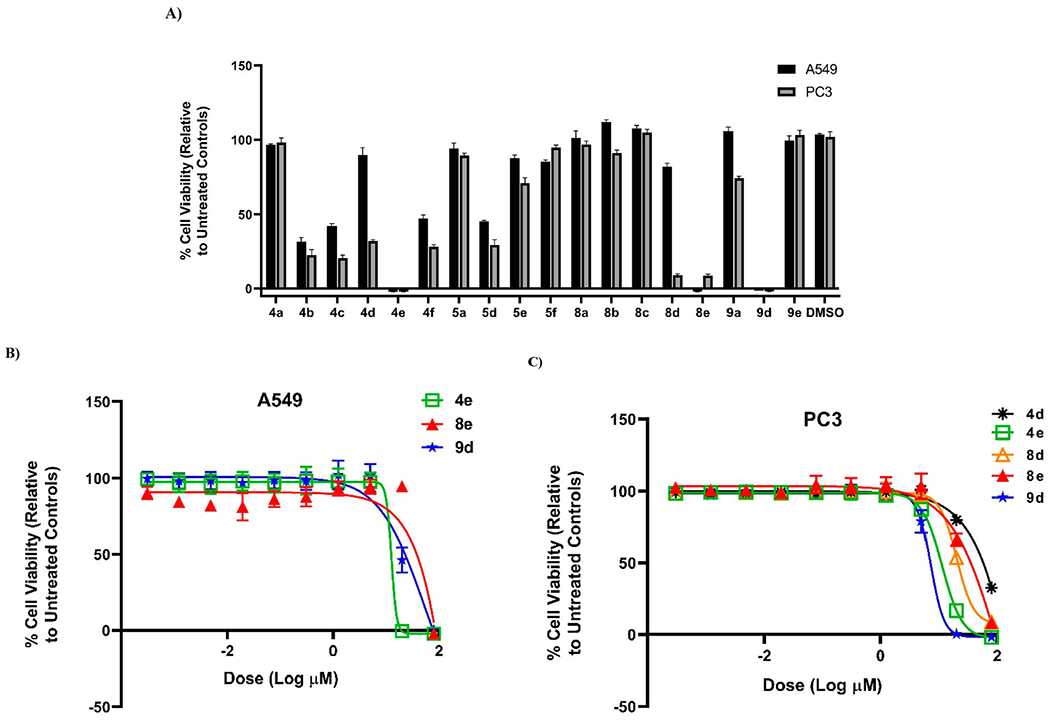
(A) % Viability of A549 (non-small lung) and PC3 (prostate) human cancer cell lines (after 72 h) at 80 μM concentration of compounds **4a–f**, **5a**, **5d–f**, **8a–e**, **9a**, and **9d–e**. (B) Dose–response of compounds **4e**, **8e**, and **9d** against A549 (non-small cell lung) human cancer cell line (72 h). (C) Dose–response of compounds **4d**, **4e**, **8d**, **8e**, and **9d** against PC3 (prostate) human cancer cell line (72 h).

Next, anti-proliferative potency of our synthesized compounds was tested against A549 (non-small lung) and PC3 (prostate) human cancer cell lines using also resazurin-based cytotoxicity assay as reported by Thorson research group [50–52] (Table 2, Figure 3). Similar trend was detected with **4e** (the most active) against A549 (IC_50_ = 12.94 ± 1.51 μM), whilst **9d** was found to be the best toward PC3 (IC_50_ = 7.31 ± 0.48 μM). From overall cytotoxicity results, compound **8d** that bearing malononitrile fragment and morpholino unit showed selectivity against PC3 (IC_50_ = 20.16 ± 0.07 μM), while **8e** that embraced 4-AMP core instead of morpholine ring was selective toward KB-3-1 cell line. The remaining molecules pretended with low cytotoxic effect. Grounded on these outcomes, we concluded that the 4-AMP and morpholine moieties would be the optimal cyclic secondary amines in this study.

#### 2.2.2. VEGFR-2 and EGFR inhibitory activity

**Figure 4.**
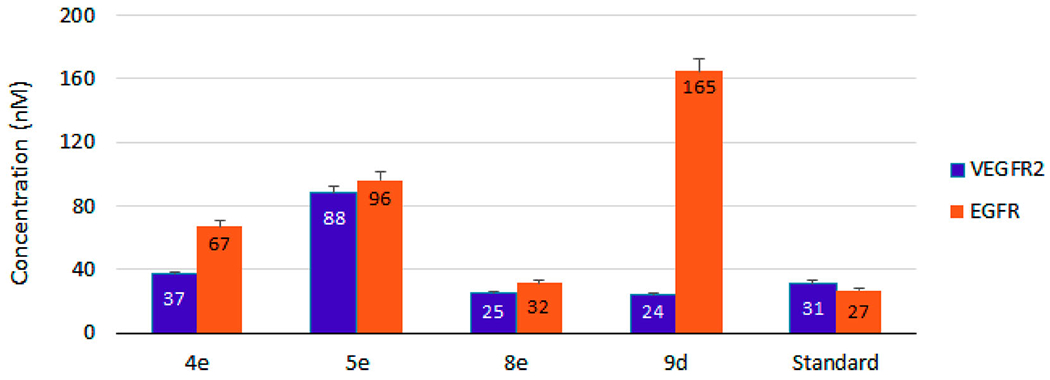
IC_50_ of compounds **4e**, **5e**, **8e**, and **9d** as inhibitors to the VEGFR-2 and EGFR human kinases. Sorafenib is the standard inhibitor to VEGFR-2 enzyme, while erlotinib is the equivalent in the case of the EGFR.

Overexpression and/or hyperactivation of VEGFR-2 and EGFR kinases are strongly associated with several malignant cancers such as non-small cell lung carcinoma (NSCLC), breast, ovarian, colon, and prostate cancers [53]. Targeting one or both of them is a promising pathway to explore more anti-angiogenic inhibitors [54,55]. In the present study (Table 3), our examined compounds; **4e**, **5e**, **8e**, and **9d** were more convenient to VEGFR-2 than EGFR. They showed VEGFR-2 inhibitory activities ranging from 24 to 88 nM while IC_50_ values with EGFR were 32–165 nM. Figure 4 and Table 3 display remarkable inhibition potency toward VEGFR-2 by **8e** (IC_50_ = 24.67 ± 1.1 nM) and **9d** (IC_50_ = 24.26 ± 1.1 nM). They are able to inactivate VEGFR-2 by 20% enhancement (0.8-fold) over the standard sorafenib (IC_50_ = 31.08 ± 1.8 nM). Concerning the EGFR inhibitory activity, the power of **8e** was also comparable to the standard erlotinib (1.2-fold). Among the tested compounds, **9d** recorded the lower inhibitory activity against EGFR (IC_50_ = 165.00 ± 8.0 nM), confirming the selectivity of **9d** toward VEGFR-2.

The most potent one against KB-3-1 and A549 cell lines (Table 2), **4e** showed 1.2 and 2.5-fold inactivation toward VEGFR-2 and EGFR, respectively. Moderate activities were also obtained by **5e** against the examined kinases. Regarding COVID-19, *in silico* studies have predicted higher affinity between the viral spike glycoprotein (S) and the inhibitors of the hepatocyte growth factor receptor (HGFR/c-MET), VEGFR and EGFR [56]. Consequently, exploring more agents against the above receptors is more than welcome.

### 2.3. In silico studies

#### 2.3.1. Physicochemical descriptors, drug-likeness, and medicinal chemistry friendliness

*In silico* computational prediction represents a robust, fast, and cost-effective approach to find new ligands or drug leads as a part in drug discovery and development strategy. Motivated by the aforementioned biological results and to explore the drug-likeness, lead-likeness and biological target of the active molecules (**4e**, **5e**, **8d,e**, and **9d**), *in silico* studies using SwissADME platform tools were effectuated [57]. To our delight, the estimated compounds show the optimal range for each physicochemical property where, the molecular weight as an indication for the size parameter is between 150 and 500 g/mol (MW, 276.24–367.40), flexibility: no more than 9 rotatable bonds (nROTB, 2-6), saturation: fraction of carbons in the *sp*^3^ hybridization not less than 0.25 (Fraction Csp3, 0.31–0.38), except **8d** is very close (Fraction Csp3 = 0.24), polarity: topological polar surface area is between 20 and 130 Å^2^ (TPSA, 71.34–104.36) (Table 4).

Lipophilicity is a significant physicochemical parameter quantified by the partition coefficient Log *P*_o/w_ between water and *n*-octanol that shows a good indicator of permeability across the cell wall [58]. Our estimated compounds manifested in XLOGP3 model Log *P*_o/w_ values between −0.7 and +5.0, ranging from 1.42 to 2.15, suggesting good permeability and absorption through the cell membrane of infected cells. Moreover, solubility is one pivotal property affecting the absorption and impacts many processes in drug development activities such as handling and formulation [59]. Concerning qualitative water solubility, all the predicted molecules based on ESOL topological model are soluble (Table 4).

**Figure 5.**
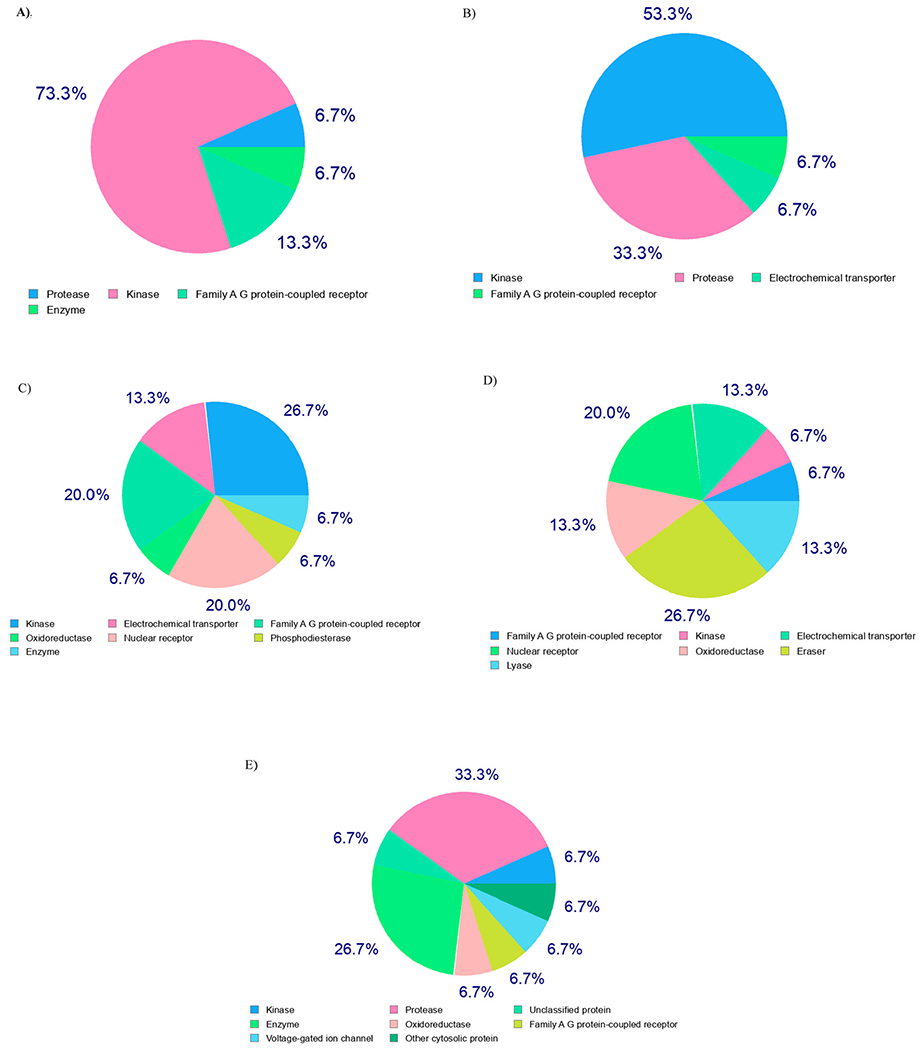
Ligand-based target prediction by SwissTarget-Prediction web tool; (A) **4e**, (B) **5e**, (C) **8d**, (D) **8e**, and (E) **9d**.

In light of the above informative predictions, the estimated compounds (**4e**, **5e**, **8d,e**, and **9d**) passed all the drug-likeness metrics (Lipinski, Ghose, Veber, Egan, and Muegge) and have the same bioavailability score (0.55). As a consequence, they could be possible drug lead candidates. Regarding with medicinal chemistry parameters, recognition of potentially problematic fragments based on Pan Assay Interference Structures (PAINS) shows zero alerts for all the predicted compounds, confirming the safety and metabolic stability of them. According to the rule of three (RO3), all compounds are lead-likeness excepting compound **4e** have one violation for this rule (MW>350), therefore, **4e** as a lead hopping and potent compound will be subjected to lead optimization and extra studies in the future. In the quest for the biological target, we used SwissTarget-Prediction web tool to predict the most probable targets of our bioactive small molecules (**4e**, **5e**, **8d**,**e**, and **9d**) [60]. The website performs target fishing using ligand-based target prediction method that based on the molecular similarity principle and the results are shown in Figure 5.

#### 2.3.2. Docking study

**Figure 6.**
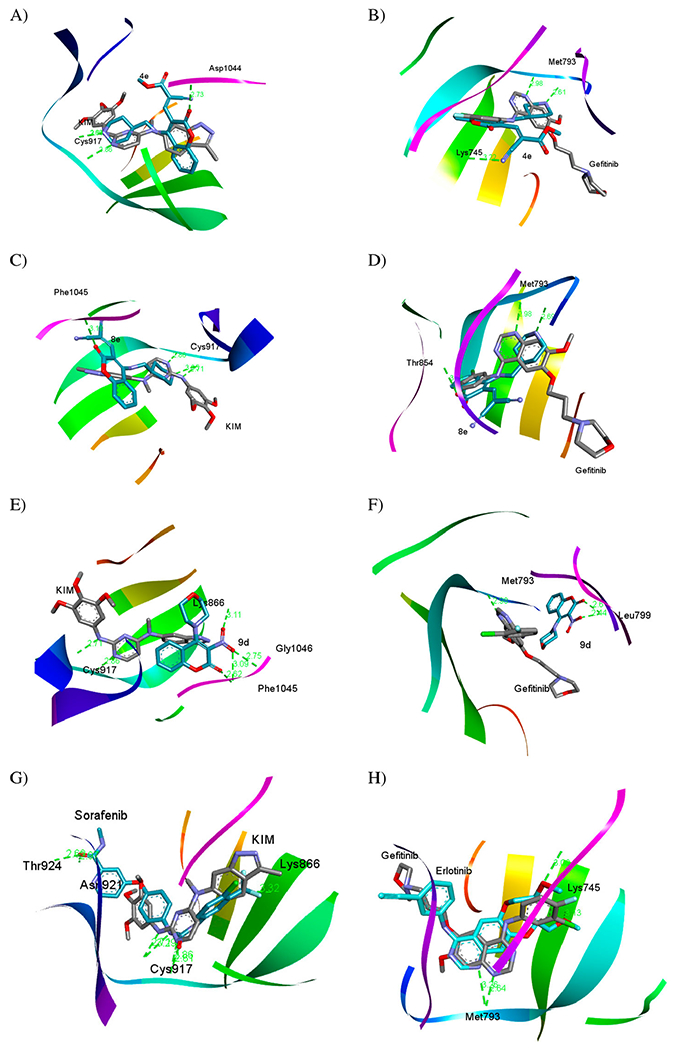
(A, C, E and G) Three-dimensional docking poses of **4e**, **8e**, **9d**, and sorafenib (cyan), respectively, with KIM (grey) within the binding site of VEGFR-2 (PDB ID: 3CJG). (B, D, F and H) Three-dimensional binding modes of **4e**, **8e**, **9d**, and erlotinib (cyan), respectively, with gefitinib (grey) within the active site of EGFR (PDB ID: 4WKQ). H-bonds are denoted by dashed lines in green. All pictures were prepared with Discovery Studio Visualizer Client 2020, and are simple for clarity of presentation.

The results that emanated from the biological evaluation exhibit that **4e** is the best in the cellular assay toward cancerous cell lines and **8e** is the most potent against VEGFR-2 and EGFR in enzymatic assay as well as taking in our account the biological target prediction (Figure 5) and the previous literatures [4,5,61,62], docking study of **4e**, **8e**, and **9d** against VEGFR-2 (KDR) and EGFR receptor tyrosine kinases as targeted therapy was implemented using *i*GEMDOCK program version 2.1 [63]. As a potent VEGFR-2 inhibitor, the crystal structure of VEGFR-2 kinase domains in complex with *N^4^*-methyl-*N^4^*-(3-methyl-1*H*-indazol-6-yl)-*N^2^*-(3,4,5-trimethoxy-phenyl)pyrimidine-2,4-diamine (three-letter code: KIM) (PDB ID: 3CJG), manifests that the indazole moiety fit well into the inside pocket of VEGFR-2. In the hinge region, the pyrimidine N-1 and the C-2 anilino N–H were predicted to make two significant hydrogen acceptor (2.86 Å) and donor (2.71 Å) bonds with the peptide backbone of Cys917, respectively (Figure 6A). Gefitinib (Iressa®) is a selective inhibitor of EGFR tyrosine kinase and an oral bioavailable drug utilized for certain breast, lung and other cancers [6,64,65]. The docked pose of gefitinib bound to EGFR (PDB ID: 4WKQ) shows that the quinazoline ring occupies the same region as the ATP purine ring (ATP-competitive inhibitor), and its ring N-1 makes an important hydrogen bond with the backbone nitrogen of Met793 (2.98 Å) (Figure 6B).

Thereafter, we docked **4e**, **8e**, and **9d** with VEGFR-2 and EGFR binding sites to compare the docking pose with co-crystallized ligands KIM and gefitinib, respectively. Table 5 and Figure 6A demonstrate that compound **4e** is mostly overlapped with co-crystallized KIM into the binding site of VEGFR-2 by predicted fitness (total energy) value of – 104.204 kcal/mol comparable to KIM with the fitness value of −102.401 kcal/mol. Also, **4e** docking mode with binding pocket of VEGFR-2 produced two conventional H-bonds, one among them in the hinge region between donor NH-1 of 4-AMP unit and carbonyl oxygen of the key amino acid residue Cys917 (2.60 Å), while the second one is observed between C-2 oxygen of coumarin moiety and peptide backbone of Asp1044 (2.73 Å) as a part of the DFG cavity. Consequently, these interactions proving the importance of 4-AMP and coumarin fragments in **4e**.

As amply illustrated in Figure 6B, **4e** is overlapped with gefitinib in the active binding site of EGFR (PDB ID: 4WKQ) by the fitness value of −112.020 kcal/mol comparable to gefitinib with the fitness value of −104.299 kcal/mol (Table 5). Analysis of **4e** docking mode in the binding pocket of EGFR shows two conventional H-bonds. In the same manner, NH-1 of 4-AMP core is stabilized by hydrogen bond with the carbonyl oxygen of the key amino acid residue Met793 (2.61 Å) in the hinge region. The second one is generated between cyano nitrogen (CN) in the side chain as a proton acceptor and the distal NH_2_ of Lys745 (3.22 Å). Moreover, the coumarin moiety inserts into a hydrophobic pocket.

Like **4e**, NH-1 of 4-AMP fragment in compound **8e** interacts with Cys917 (3.35 Å) in the binding cavity of VEGFR-2 (−100.502 kcal/mol) and Met793 (2.69 Å) in EGFR (−113.558 kcal/mol) via hydrogen bonding. At the same time, coumarin moiety plays an important role for interaction stabilizing between **8e** and kinases, where its carbonyl oxygen forms H-bond with Phe1045 (3.18 Å) in VEGFR-2 and O-1 act as a proton acceptor from Thr854 (3.08 Å) in EGFR (Figure 6C and D).

The binding mode of compound **9d** against VEGFR-2 (Figure 6E) and EGFR (Figure 6F) explains why **9d** was a selective and highly potent nanomolar inhibitor of VEGFR-2 with the IC_50_ value of 24.26 ± 1.1 nM (Table 3). Figure 6E shows that **9d** was overlapped with co-crystallized ligand KIM via coumarin unit only and the nitro group fit into the outside pocket of VEGFR-2. Thus, carbonyl coumarin and nitro group as hydrogen acceptors were predicted to form new four H-bonds with amino acid residues Lys866, Phe1045, and Gly1046. Noteworthy, **9d** did not form the foremost H-bond with Cys917, confirming the importance of 4-AMP unit and establishing a new pathway to suppress VEGFR-2. On the contrary, Figure 6F displays that **9d** did not overlap with gefitinib within the binding site of EGFR, thus, this interaction pattern dramatically decreases the activity of **9d** toward EGFR kinase (IC_50_= 165.00 ± 8.0 nM).

Finally, the docking modes of sorafenib and erlotinib as the reference drugs in VEGFR-2 and EGFR inhibitory activity assay, respectively, were predicted (Table 5, Figure 6G,H). Sorafenib and erlotinib demonstrated high binding affinity toward VEGFR-2 and EGFR, respectively, as well displayed the major H-bonds in the hinge region with the key amino acid residues Cys917 and Met793, respectively, as generated with our compounds binding modes.

### 2.4. Structure–activity relationship

As stated above in the biological evaluation and predicted docking modes of compounds **4e**, **8e**, and **9d**, structure–activity relationship (SAR) study on the cyclic secondary amine fragment shows that 4-AMP and morpholine units are the optimal for cellular and enzymatic potencies. Compound **4e** with 4-AMP and methyl cyanoacrylate cores has good anti-proliferative activity toward all the three tumor cell lines and more active against VEGFR-2 than EGFR. When methyl cyanoacrylate fragment is replaced by malononitrile and 4-AMP is retained, the generated compound **8e** is selective toward KB-3-1 and a promising dual inhibitor VEGFR-2 and EGFR. Compound **9d** with morpholine and nitro groups is effective against A549 and PC3 cell lines, but selective inhibitor of VEGFR-2 (IC_50_ = 24.26 ± 1.1 nM) in comparison with EGFR (IC_50_= 165.00 ± 8.0 nM). When 3-NO_2_ group in **9d** is changed by methylene malononitrile unit, the corresponding compound **8d** is selective toward PC3 cell line (Table 2). It should be noted that, when methylene malononitrile unit in **8e** is substituted by formyl group, the obtained small molecule **5e** showed lower inhibitory activity against VEGFR-2 and EGFR kinases (Table 3).

**Figure 7.**
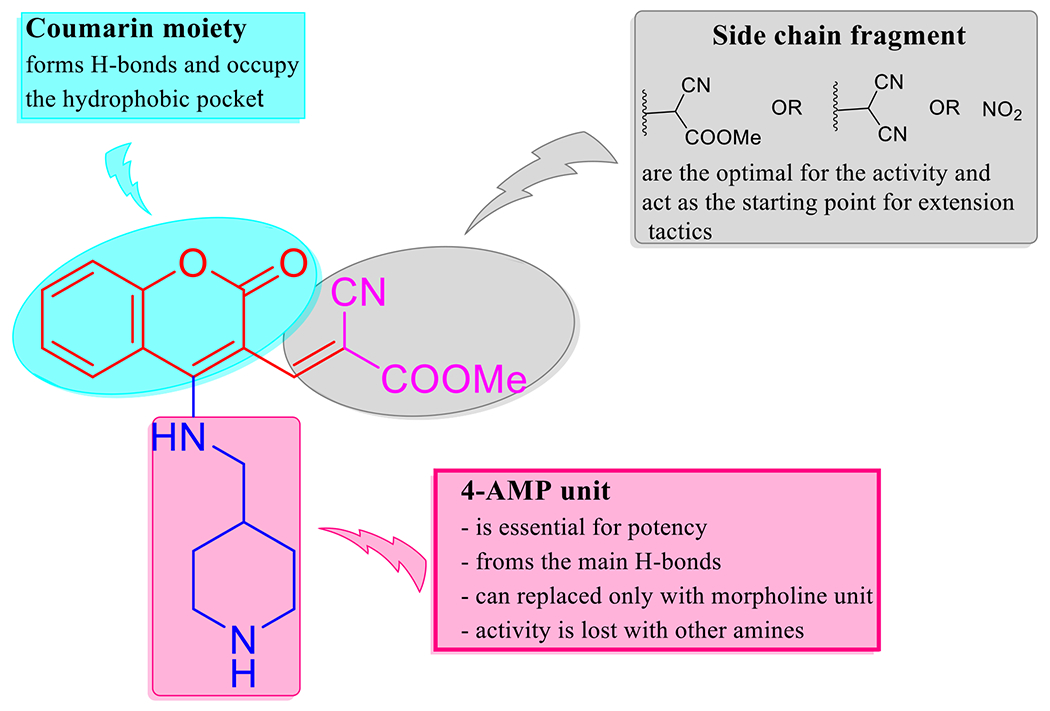
The SAR study of the synthesized compounds.

In cellular anti-proliferative assay, a big drop-in activity is detected when 4-AMP and morpholine units are replaced with other amines. As illustrated by the docking study, coumarin framework is an important for potent activity, where it forms H-bonds and suitable to occupy the hydrophobic pocket, thereby improving the binding affinity of the hit molecules to the therapeutic targets. Through the inclusive valuation, cyclic secondary amines including 4-AMP and morpholine are crucial for maintaining the potency of the target small molecules in our study. As well side chain fragments encompassing methyl cyanoacetate, malononitrile and nitro group have an obviously impact on cellular and enzymatic activities. Coumarin system can be considered good bioisostere of quinazoline scaffold to discovery and development of EGFR potent inhibitors. These finding tell us the useful pathway to create a rational design using extension tactics in order to achieve the optimal binding interactions, hence get more potent VEGFR-2 and EGFR inhibitors (Figure 7).

## 3. Materials and methods

### 3.1. Materials and instruments

Melting points were determined on a BÜCHI Melting Point B-540 apparatus in open capillaries and are uncorrected (BÜCHI Germany). NMR spectra (^1^H NMR and ^13^C NMR) were recorded on Bruker Avance DRX 500HD MHz (^1^H: 500 MHz, ^13^C: 125 MHz) spectrometer (Bruker, USA) at 298 K. Tetramethylsilane (TMS) is used for internal calibration (^1^H NMR and ^13^C NMR: 0.00 ppm). Chemical shifts were reported in parts per million (ppm) on the δ scale and relative to residual solvent peaks (DMSO-*d*_6_: ^1^H: 2.50 ppm, ^13^C: 39.5 ppm). Coupling constants (*J*) are reported in Hz with the following abbreviations used to indicate splitting: s = singlet, d = doublet, t = triplet, q = quartet, m = multiplet, br = broad signal. ESI mass spectra were recorded using an ion trap mass spectrometer equipped with a standard ESI/APCI source. Samples were introduced by direct infusion with a syringe pump. Nitrogen served both as the nebulizer gas and the dry gas. Nitrogen was generated by a nitrogen generator. Helium served as cooling gas for the ion trap and collision gas for MS^n^ experiments (Bruker Daltonik GmbH, Bremen, Germany). Starting materials and reagents were obtained from commercial sources and used without further purification, unless otherwise indicated. Solvents were dried and purified following standard procedures in organic chemistry. The purity of the synthesized compounds was investigated by TLC, performed on Merck precoated silica gel 60 F_254_ aluminum sheets with a solvent mixture of DCM-MeOH (982) as eluent. Spots were visualized under UV illumination at 254 and 366 nm.

### 3.2. Synthesis and characterization

#### 3.2.1. Synthesis of 4-chloro-3-formylcoumarin (1a)

Our substrate **la** was synthesized from 4-hydroxycoumarin according to the Vilsmeier–Haack protocol [36].

#### 3.2.2. General procedure for the synthesis of methyl (E)-2-cyano-3-(4-substitutedcoumarinyl)acrylates 4a–f

Methyl cyanoacetate (**2a**, 1 mmol) was treated with 4-chloro-3-formylcoumarin (**la**, 1.0 mmol) and cyclic secondary amines (**3a–f**, 2.5 mmol) in MeOH (2 mL). Using the open flask, the mixture was stirred at room temperature for 30-45 min. After the reaction was completed (TLC), the solvent was removed under reduced pressure and the crude product was purified by simple recrystallization (MeOH) or washing with hot methanol to afford the desired compounds.

Methyl (*E*)-2-cyano-3-(2-oxo-4-(pyrrolidin-1-yl)-2*H*-chromen-3-yl)acrylate (**4a**)

Yield 93%; yellow solid; mp 230–232 °C; ^1^H NMR (500 MHz, DMSO-*d*_6_) δ 8.44 (s, 1H), 8.04 (dd, *J* = 8.1, 1.5 Hz, 1H), 7.67 (ddd, *J* = 8.5, 7.3, 1.4 Hz, 1H), 7.43–7.36 (m, 2H), 3.77 (s, 3H), 3.34–3.33 (m, 4H, *overlapped with water of DMSO*), 2.05–2.01 (m, 4H); ^13^C NMR (125 MHz, DMSO-*d*_6_) δ 164.7 (C4—N), 161.6 (C = O ester), 158.7 (C = O lactone), 151.5 (C—O lactone), 151.1 (CH olefinic), 133.2, 126.7, 124.1, 118.6, 117.7, 116.5, 93.9, 90.1, 56.1 (OCH_3_), 52.8 (2CH_2_N), 25.0 (2CH_2_); (+)-ESI-MS, *m/z* (%): 347 [M + Na]^+^ (100), 671 [2M + Na]^+^ (31). See Supporting Information, Figs. S1–S3.

Methyl (*E*)-2-cyano-3-(2-oxo-4-(piperidin-1-yl)-2*H*-chromen-3-yl)acrylate (**4b**)

Yield 91%; faint orange solid; mp 192–194 °C; ^1^H NMR (500 MHz, DMSO-*d*_6_) δ 8.17 (s, 1H), 7.93 (dd, *J* = 8.2, 1.5 Hz, 1H), 7.70 (ddd, *J* = 8.5, 7.2, 1.4 Hz, 1H), 7.44–7.38 (m, 2H), 3.85 (s, 3H), 3.68–3.64 (m, 4H), 1.77–1.73 (m, 6H); ^13^C NMR (125 MHz, DMSO-*d*_6_) δ 163.5 (C4—N), 163.1 (C = O ester), 158.9 (C = O lactone), 153.4 (C—O lactone), 152.5 (CH olefinic), 134.0, 127.3, 124.6, 118.0, 117.5, 115.8, 102.8, 100.9, 54.8 (2CH_2_N), 53.5 (OCH_3_), 27.0 (2CH^2^), 23.6 (CH_2_); (+)-ESI-MS, *m/z* (%): 361 [M + Na]^+^ (100), 699 [2M + Na]^+^ (6). See Supporting Information, Figs. S4–S6.

Methyl (*E*)-2-cyano-3-(4-(4-hydroxypiperidin-1-yl)-2-oxo-2*H*-chromen-3-yl)acrylate (**4c**)

Yield 96%; orange solid; mp 194–196 °C; ^1^H NMR (500 MHz, DMSO-*d*_6_) δ 8.16 (s, 1H), 7.92 (dd, *J* = 8.2, 1.6 Hz, 1H), 7.70 (ddd, *J* = 8.4, 7.2, 1.4 Hz, 1H), 7.44–7.38 (m, 2H), 4.97 (d, *J* = 3.7 Hz, 1H, OH), 3.89 (dq, *J* = 7.8, 3.8 Hz, 1H), 3.85 (s, 3H, OCH_3_), 3.81 (ddd, *J* = 12.7, 6.3, 3.0 Hz, 2H), 3.58 (ddd, *J* = 12.7, 8.6, 3.3 Hz, 2H), 2.00–1.93 (m, 2H), 1.69–1.60 (m, 2H); ^13^C NMR (125 MHz, DMSO-*d*_6_) δ 163.5 (C4—N), 162.9 (C = O ester), 158.9 (C = O lactone), 153.3 (C—O lactone), 152.5 (CH olefinic), 134.0, 127.2, 124.6, 118.0, 117.5, 115.8, 102.8, 100.9, 64.7 (CHOH), 53.5 (OCH_3_), 51.2 (2CH_2_N), 35.4 (2CH_2_); (+)-ESI-MS, *m/z* (%): 377 [M + Na]^+^ (100), 731 [2M + Na]^+^ (12). See Supporting Information, Figs. S7–S9.

Methyl (*E*)-2-cyano-3-(4-morpholino-2-oxo-2*H*-chromen-3-yl)acrylate (**4d**)

Yield 92%; yellow solid; mp 218–220 °C; ^1^H NMR (500 MHz, DMSO-*d*_6_) δ 8.19 (s, 1H), 7.95 (dd, *J* = 8.3, 1.5 Hz, 1H), 7.71 (ddd, *J* = 8.5, 7.2, 1.4 Hz, 1H), 7.45 (dd, *J* = 8.3, 1.2 Hz, 1H), 7.40 (ddd, *J* = 8.4, 7.2, 1.3 Hz, 1H), 3.86 (s, 3H), (t, *J* = 4.6 Hz, 4H), 3.72 (t, *J* = 5.5, 3.7 Hz, 4H); ^13^C NMR (125 MHz, DMSO-*d*_6_) δ 163.4 (C4—N), 162.4 (C = O ester), 158.8 (C = O lactone), 153.4 (C—O lactone), 151.9 (CH olefinic), 134.1, 127.3, 124.7, 118.0 117.1, 115.7, 103.8, 101.3, 67.22 (2CH_2_O), 53.7 (2CH_2_N), 53.5 (OCH_3_); (+)-ESI-MS, *m/z* (%): 363 [M + Na]^+^ (100), 703 [2M + Na]^+^ (25). See Supporting Information, Figs. S10–S12.

Methyl (*E*)-2-cyano-3-(2-oxo-4-((piperidin-4-ylmethyl)amino)-2H-chromen-3-yl)acrylate (**4e**)

Yield 96%; yellow solid; mp 172–174 °C; ^1^H NMR (500 MHz, DMSO-*d*_6_) δ 8.76 (t, *J* = 5.8 Hz, 1H), 8.64 (s, 1H), 8.34 (dd, *J* = 7.9, 1.7 Hz, 1H), 7.68 (ddd, *J* = 8.6, 7.3, 1.7 Hz, 1H), 7.44 (td, *J* = 7.6, 1.1 Hz, 1H), 7.39 (dd, *J* = 8.2, 1.0 Hz, 1H), 3.90 (s, 3H), 3.59 (t, *J* = 6.2 Hz, 2H), 2.95 (dt, *J* = 12.1, 3.4 Hz, 2H), 2.44 (td, *J* = 12.0, 2.5 Hz, 2H), 1.82–1.72 (m, 2H), 1.66 (dd, *J* = 12.6, 3.5 Hz, 2H), 1.23–1.17 (m, 1H), 1.17–1.12 (m, 1H); ^13^C NMR (125 MHz, DMSO-*d*_6_) δ 166.5 (C4—NH), 160.1 (C=O ester), 159.71 (C=O lactone), 155.0 (C—O lactone), 153.9 (CH olefinic), 142.1, 133.8, 125.25, 125.17, 118.8, 117.5, 107.8, 105.8, 53.0 (OCH3), 47.1 (CH_2_NH), 46.3 (2CH_2_NH), 36.7 (CH aliphatic), 31.3 (2CH_2_); (+)-ESI-MS, *m/z* (%): 368 [M + H]*+* (100), 735 [2M + H]*+* (6). See Supporting Information, Figs. S13–S15.

Methyl (*E*)-2-cyano-3-(4-methoxy-2-oxo-2*H*-chromen-3-yl)acrylate (**4f**)

Yield 89%; yellow solid; mp 148–150 °C; ^1^H NMR (500 MHz, DMSO-*d*_6_) δ 8.34 (s, 1H), 7.97 (dd, *J* = 8.0, 1.6 Hz, 1H), 7.78 (ddd, *J* = 8.7, 7.2, 1.6 Hz, 1H), 7.49 (dd, *J* = 8.4, 1.1 Hz, 1H), 7.46 (ddd, *J* = 8.3, 7.3, 1.1 Hz, 1H), 4.13 (s, 3H), 3.89 (s, 3H); ^13^C NMR (125 MHz, DMSO-*d*_6_) δ 167.2, 162.0, 159.9, 150.5, 149.3, 133.2, 126.2, 125.1, 117.9, 117.2, 116.7, 103.9, 99.2, 63.8, 52.3; (+)-ESI-MS, *m/z* (%): 308 [M + Na]^+^ (100), 593 [2M + Na]*+* (22). *See* Supporting Information, Figs. S16–S18.

#### 3.2.3. General procedure for the synthesis of buta-1,3-diene-1,1,3-tricarbonitrile derivatives 8a,b and methylene malononitrile analogs 8c–e

Using open flask, a solution of malononitrile (**2b**, 2 mmol) and cyclic secondary amines (**3a–e**, 1 mmol) in MeOH (2 mL) was stirred at room temperature for 10 min. At the same time, anther mixture of 4-chloro-3-formylcoumarin (**1a**, 1.0 mmol) and the same cyclic secondary amines (**3a–e**, 1.5 mmol) in MeOH (2 mL) was also produced by stirring at ambient temperature for 10 min. Next, the two mixtures were combined together and sequential stirring was continued at room temperature until a new product was formed (TLC). Hence, the solvent was removed under reduced pressure using the rotary evaporator and the crude product was purified by simple recrystallization (MeOH) or only washing with hot methanol to get the desired compound.

(*Z*)-2-amino-4-(2-oxo-4-(pyrrolidin-1-yl)-2*H*-chromen-3-yl)buta-1,3-diene-1,1,3-tricarbonitrile (**8a**)

Yield 97%; yellow solid; mp > 320 °C; ^1^H NMR (500 MHz, DMSO-*d*_6_) δ 8.72 (s, 1H), 7.85 (dd, *J* = 8.2, 1.5 Hz, 1H), 7.73 (ddd, *J* = 8.5, 7.2, 1.5 Hz, 1H), 7.63 (s, 2H), 7.56 (dd, *J* = 8.4, 1.3 Hz, 1H), 7.45 (ddd, *J* = 8.3, 7.2, 1.3 Hz, 1H), 3.72–3.58 (m, 2H), 3.29–2.95 (m, 2H), 1.97–1.86 (m, 2H), 1.86–1.62 (m, 2H); ^13^C NMR (125 MHz, DMSO-*d*_6_) δ 166.51(C—NH_2_), 161.8 (C4—N), 159.5, 155.9, 155.3, 152.4, 134.0, 133.5, 126.1, 125.9, 120.6, 118.9, 116.7, 116.6, 103.7, 76.4, 48.1, 46.1, 25.9, 24.6; (+)-ESI-MS, *m/z* (%): 737 [2M + Na]*+* (90), 1094 [3M + Na]^+^ (2). See Supporting Information, Figs. S19–S21.

(*Z*)-2-amino-4-(2-oxo-4-(piperidin-1-yl)-2*H*-chromen-3-yl)buta-1,3-diene-1,1,3-tricarbonitrile (**8b**)

Yield 94%; yellow solid; mp 308–310 °C; ^1^H NMR (500 MHz, DMSO-*d*_6_) δ 8.67 (s, 1H), 7.92 (dd, *J* = 8.3, 1.5 Hz, 1H), 7.73 (ddd, *J* = 8.5, 7.2, 1.5 Hz, 1H), 7.65–7.60 (m, 2H), 7.56 (dd, *J* = 8.4, 1.2 Hz, 1H), 7.46 (ddd, *J* = 8.3, 7.1, 1.3 Hz, 1H), 3.84–3.73 (m, 2H), 3.30–3.21 (m, 1H), 3.21–3.13 (m, 1H), 1.69–1.58 (m, 4H), 1.46–1.36 (m, 1H), 1.26–1.16 (m, 1H); ^13^C NMR (125 MHz, DMSO-*d*_6_) δ 166.8, 161.7, 159.5, 155.9, 155.0, 152.4, 134.0, 133.9, 126.5, 125.8, 119.6, 118.9, 116.7, 116.5, 103.9, 76.3, 47.8, 26.3, 24.4; (+)-ESI-MS *m/z* (%): 372 [M + H]^+^. See Supporting Information, Figs. S22–S24.

2-((4-(4-Hydroxypiperidin-1-yl)-2-oxo-2*H*-chromen-3-yl)methylene)malononitrile (8c)

Yield 88%; yellow solid; mp 294–296 °C; ^1^H NMR (500 MHz, DMSO-*d*_6_) δ 8.97 (dd, *J* = 8.3, 1.5 Hz, 1H), 8.76 (s, 1H), 7.81 (ddd, *J* = 8.5, 7.2, 1.5 Hz, 1H), 7.53 (ddd, *J* = 8.4, 7.3, 1.3 Hz, 1H), 7.47 (dd, *J* = 8.4, 1.3 Hz, 1H), 5.77–5.76 (m, 2H), 3.18–3.17 (m, 4H), 2.10–2.09 (m, 4H); ^13^C NMR (125 MHz, DMSO-*d*_6_) δ 161.9, 158.4, 153.3, 148.7, 147.9, 135.8, 126.0, 125.6, 118.9, 117.3, 115.0, 103.0, 93.5, 55.4, 49.1, 31.2; (+)-ESI-MS, *m/z* (%): 344 [M + Na]^+^ (100), 665 [2M + Na]^+^ (12). See Supporting Information, Figs. S25–S27.

2-((4-Morpholino-2-oxo-2*H*-chromen-3-yl)methylene)malononitrile (**8d**) [39]

Yield 96%; yellow solid; mp 196–198 °C; (from literature [39], yield 52%; orange solid, mp 193–195 °C). (+)-ESI-MS, *m/z* (%):637 [2M + Na]^+^ (100). See Supporting Information, Figure S28.

2-((2-Oxo-4-((piperidin-4-ylmethyl)amino)-2*H*-chromen-3-yl)methylene)malononitrile (8e)

Yield 98%; yellow solid; mp 154–156 °C; ^1^H NMR (500 MHz, DMSO-*d*_6_) δ 8.53 (s, 1H), 8.32 (dd, *J* = 7.9, 1.7 Hz, 1H), 7.69 (ddd, *J* = 8.7, 7.4, 1.8 Hz, 2H), 7.48–7.43 (m, 1H), 7.40 (d, *J* = 8.3 Hz, 1H), 3.51 (d, *J* = 6.7 Hz, 2H), 2.98 (dt, *J* = 12.4, 3.3 Hz, 2H), 2.50–2.43 (m, 2H), 1.89–1.81 (m, 1H), 1.71–1.63 (m, 3H), 1.23–1.11 (m, 2H); ^13^C NMR (125 MHz, DMSO-*d*_6_) δ 159.6, 159.4, 154.5, 153.8, 145.4, 134.0, 125.3, 118.7, 117.5, 116.6, 115.9, 106.2, 93.4, 47.5, 46.0, 36.2, 30.8; (+)-ESI-MS, *m/z* (%): 335 [M + H]^+^ (100), 669 [2M + H]^+^ (1). See Supporting Information, Figs. S29–S31.

#### 3.2.4. General procedure for the synthesis of 4-substituted-3-formyl/nitro-2H-chromen-2-ones 5a, d–f and 9a,d–f

Cyclic secondary amines (**3a,d-f**, 1 mmol) was added to a stirred solution of 4-chloro-3-formyl/nitrocoumarin (**1a,c**, 1.0 mmol) and triethylamine (Et_3_N, 1 mmol) in MeOH (2 mL). The reaction mixture was stirred at room temperature for 5–15 min. After the reaction was completed (TLC), the solvent was removed under reduced pressure and the crude product was purified by simple recrystallization (MeOH) or washing with hot methanol to afford the desired compound.

4-Pyrrolidino-3-formyl-2*H*-chromen-2-one (5a)

Yield 93%; white solid; mp 190–192 °C; ^1^H NMR (500 MHz, DMSO-d_6_) δ 9.67 (s, 1H), 8.06 (dd, *J* = 8.1, 1.5 Hz, 1H), 7.63 (ddd, *J* = 8.5, 7.3, 1.4 Hz, 1H), 7.36 (dd, *J* = 8.3, 1.2 Hz, 1H), 7.33 (ddd, *J* = 8.4, 7.3, 1.3 Hz, 1H), 3.85 (s, 4H), 1.99–1.93 (m, 4H); ^13^C NMR (125 MHz, DMSO-*d*_6_) δ 183.4 (CHO), 163.7 (C4—N), 158.0 (C=O lactone), 152.2 (C—O lactone), 133.0, 127.9, 123.5, 118.1, 117.5, 97.4, 56.7 (2CH_2_N), 24.9 (2CH_2_); (+)-ESI-MS, *m/z* (%): 266 [M+ Na^+^ (100), 509 [2M + Na]^+^ (35). *See* Supporting Information, Figs. S32–S34.

4-Morpholino-3-formyl-2*H*-chromen-2-one (5d)

Yield 95%; yellow solid; mp 150–152 °C; ^1^H NMR (500 MHz, DMSO-*d*_6_) δ 9.85 (s, 1H), 7.99 (dd, *J* = 8.2, 1.5 Hz, 1H), 7.69 (ddd, *J* = 8.5, 7.2, 1.4 Hz, 1H), 7.41 (dd, *J* = 8.4, 1.3 Hz, 1H), 7.37 (ddd, *J* = 8.4, 7.2, 1.3 Hz, 1H), 3.90–3.86 (m, 4H), 3.65 (t, *J* = 4.7 Hz, 4H); ^13^C NMR (125 MHz, DMSO-*d*_6_) δ 186.5 (CHO), 176.5 (C4—N), 162.5 (C=O lactone), 154.4 (C—O lactone), 134.3, 126.3, 124.1, 121.3, 117.0, 95.3, 66.8 (2CH_2_O), 54.5 (2CH_2_N); (+)-ESI-MS, *m/z*(%): 282 [M + Na]^+^ (35), 541 [2M + Na]^+^ (100). See Supporting Information, Figs. S35–S37.

4-((Piperidin-4-ylmethyl)amino)-3-formyl-2*H*-chromen-2-one (5e)

Yield 85%; yellow solid; mp 208–210 °C; ^1^H NMR (500 MHz, DMSO-*d*_6_) δ 11.94 (t, *J* = 5.1 Hz, 1H, NH_Ar_), 9.94 (s, 1H), 8.28 (d, *J* = 8.2 Hz, 1H), 7.78 (t, *J* = 7.8 Hz, 1H), 7.46–7.39 (m, 2H), 3.91 (t, *J* = 5.9 Hz, 2H), 3.33–3.23 (m, 3H), 2.90 (td, *J* = 12.8, 2.9 Hz, 2H), 2.04 (ttt, *J* = 10.4, 6.6, 3.4 Hz, 1H), 1.95 (d, *J* = 13.7 Hz, 2H), 1.58–1.44 (m, 2H); ^13^C NMR (125 MHz, DMSO-*d*_6_) δ 191.0 (CHO), 162.2 (C4—NH), 159.9 (C=O lactone), 155.2 (C—O lactone), 135.6, 129.3, 124.6, 118.5, 114.0, 96.3, 52.2 (CH_2_NH), 43.1 (2CH_2_NH), 34.6 (CH), 26.7 (2CH_2_); (+)-ESI-MS, *m/z* (%): 287 [M+ H]^+^. See Supporting Information, Figs. S38–S40.

4-Methoxy-3-formyl-2*H*-chromen-2-one (5f)

Yield 89%; pale yellow solid; mp 118–120 °C; ^1^H NMR (500 MHz, DMSO-*d*_6_) δ 10.12 (s, 1H), 7.99 (dd, *J* = 8.0, 1.6 Hz, 1H), 7.79 (ddd, *J* = 8.7, 7.2, 1.6 Hz, 1H), 7.48–7.41 (m, 2H), 4.19 (s, 3H); ^13^C NMR (125 MHz, DMSO-*d*_6_) δ 189.2 (CHO), 170.0 (C4—O), 162.3 (C=O lactone), 153.6 (C—O lactone), 135.5, 125.8, 125.4, 117.3, 117.1, 106.7, 65.6 (OCH_3_); (+)-ESI-MS, *m/z*(%): 227 [M + Na]^+^ (100), 431 [2M + Na]^+^ (7)^+^. See Supporting Information, Figs. S41–S43.

4-Pyrrolidino-3-nitro-2*H*-chromen-2-one (9a)

Yield 97%; yellow solid; mp 204–206 °C; ^1^H NMR (500 MHz, DMSO-*d*_6_) δ 8.18 (dd, *J* = 8.1, 1.4 Hz, 1H), 7.69 (ddd, *J* = 8.5, 7.2, 1.4 Hz, 1H), 7.41–7.36 (m, 2H), 3.83–3.77 (m, 4H), 2.01–1.95 (m, 4H); ^13^C NMR (125 MHz, DMSO-*d*_6_) δ 155.2 (C4—N), 154.4 (C=O lactone), 151.2 (C—O lactone), 133.6, 128.1, 123.8, 117.6, 117.0, 114.6, 55.6 (2 CH_2_N), 24.7 (2CH_2_); (+)-ESI-MS, *m/z*(%): 283 [M+ Na]^+^ (100), 543 [2M + Na]^+^ (17). See Supporting Information, Figs. S44–S46.

4-Morpholino-3-nitro-2*H*-chromen-2-one (9d)

Yield 96%; yellow solid; mp 210–212 °C; ^1^H NMR (500 MHz, DMSO-*d*_6_) δ 7.90 (dd, *J* = 8.1, 1.5 Hz, 1H), 7.76 (ddd, *J* = 8.6, 7.2, 1.5 Hz, 1H), 7.50–7.45 (m, 2H), 3.88–3.83 (m, 4H), 3.39–3.33 (m, 4H); ^13^C NMR (125 MHz, DMSO-*d*_6_) δ 155.5 (C4—N), 153.1 (C=O lactone), 152.5 (C—O lactone), 134.6, 127.7, 125.8, 125.5, 118.1, 116.1, 66.2 (2 CH_2_O), 51.5 (2 CH_2_N); (+)-ESI-MS, *m/z* (%): 299 [M+ Na]^+^ (100), 575 [2M+ Na]^+^ (25). See Supporting Information, Figs. S47–S49.

4-((Piperidin-4-ylmethyl)amino)-3-nitro-2*H*-chromen-2-one (9e)

Yield 89%; yellow solid; mp 208–210 °C; ^1^H NMR (500 MHz, DMSO-*d*_6_) δ 15.82 (s, 1H, NH_Ar_), 7.22–7.18 (m, 2H), 6.78–6.75 (m, 1H), 6.69 (td, *J* = 7.5, 1.2 Hz, 1H), 3.32 (d, *J* = 6.5 Hz, 2H), 3.28 (dt, *J* = 12.4, 3.4 Hz, 3H), 2.88 (td, *J* = 12.8, 3.0 Hz, 2H), 1.98–1.88 (m, 1H), 1.84 (d, *J* = 14.6 Hz, 2H), 1.36 (qd, *J* = 12.5, 4.0 Hz, 2H); ^13^C NMR (125 MHz, DMSO-*d*_6_) δ 170.0, 163.4, 162.0, 131.7, 130.0, 120.4, 117.6, 117.3, 103.3, 55.7, 49.9, 43.7, 34.5, 27.5; (+)-ESI-MS, *m/z* (%): 304 [M+ H]^+^. See Supporting Information, Figs. S50–S52.

4-Methoxy-3-nitro-2*H*-chromen-2-one (9f)

Yield 91%; white solid; mp 110–112 °C; ^1^H NMR (500 MHz, DMSO-*d*_6_) δ 7.98 (dd, *J* = 8.1, 1.6 Hz, 1H), 7.82 (ddd, *J* = 8.7, 7.3, 1.6 Hz, 1H), 7.55–7.48 (m, 2H), 4.18 (s, 3H), ^13^C NMR (125 MHz, DMSO-*d*_6_) δ 167.8 (C4—O), 158.7 (C=O lactone), 151.7 (C—O lactone), 135.4, 125.5, 123.4, 117.3, 116.5, 115.5, 60.7 (OCH3); (+)-ESI-MS, *m/z* (%): 244 [M+ Na]^+^ (100), 465 [2M+ Na]^+^ (6). See Supporting Information, Figs. S53–S55.

### 3.3. Resazurin cellular-based bioassay

Anti-cervical cancer activity of all the synthesized compounds were evaluated for the *in vitro* cytotoxicity toward KB-3-1 cell line (was obtained from the American Type Culture Collection, ATCC, Rockville, MD, USA) by resazurin-based assay [49] using (+)-griseofulvin as a positive control. Next, anti-proliferative potency of our synthesized compounds was tested against A549 (non-small lung) and PC3 (prostate) human cancer cell lines using the same assay as reported by Thorson research group [50–52].

### 3.4. Enzyme inhibitory bioassay

In the case of the VEGFR-2 enzymatic assay, we followed the instruction manual of VEGFR-2 (KDR) Kinase Assay Kit Catalog # 40325. On the other hand, EGFR Kinase Assay Ki Catalog # 40321 was followed to examine the capability of the selected compounds to inactivate the enzyme EGFR kinase. A series of 10-fold dilutions was prepared from each compound (**4e**, **5e**, **8e**, and **9d**) while sorafenib (Cat no.284461-73-0, Santa Cruz) and erlotinib (Cat no.183321-74-6, Cayman) were matched as the positive controls in synchronize to VEGFR-2 and EGFR assays, respectively. At the end of the experiment, the luminescence was measured by Tecan spark microplate reader. The compounds concentration that inactivate 50% of the measured kinases was separately, calculated using a curve fitting software; Prism, version 6.

### 3.5. In silico computational predictions

#### 3.5.1. Physicochemical descriptors, drug-likeness, and medicinal chemistry parameters

The physicochemical properties of compounds **4e**, **5e**, **8d,e**, and **9d** were predicted using SwissADME [57] as formerly described (Table 4).

#### 3.5.2. Biological target prediction

The biomolecular target of compounds **4e**, **5e**, **8d,e**, and **9d** were estimated through the SwissTarget-Prediction web tool [60] that using ligand-based target prediction approach which based on the molecular similarity principle (Table 4, Figure 5).

#### 3.5.3. Molecular docking simulation

**Figure 8.**
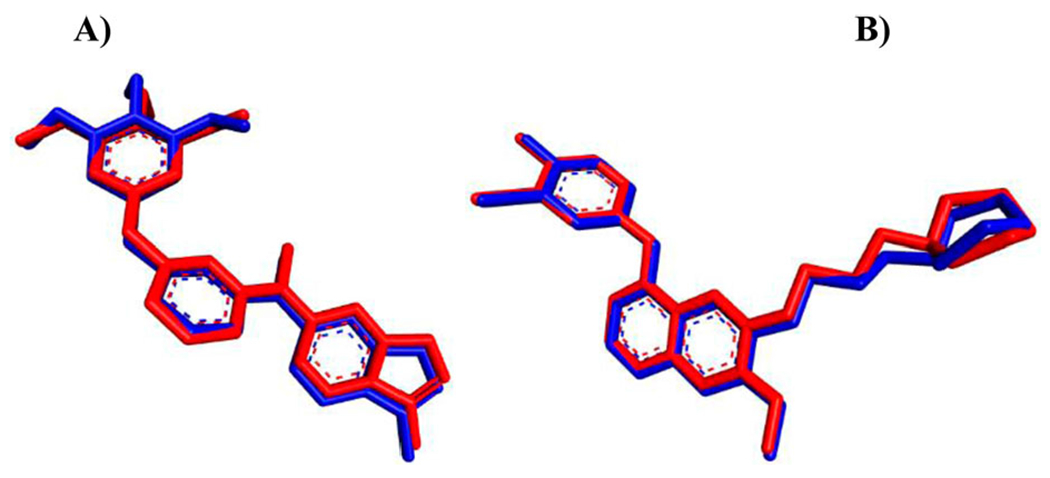
*i*GEMDOCK validation. (A) Crystal KIM (red) and the docked one (blue) display similar binding orientation in the binding pocket of VEGFR-2 with RMSD 0.4355 Å. (B) Crystal gefitinib (red) with the docked one (blue) are superimposed in EGFR binding cavity with RMSD 0.3462 Å.

*In silico* flexible ligand docking study has been performed by *i*GEMDOCK program version 2.1 (Department of Biological Science and Technology & Institute of Bioinformatics, National Chiao Tung University, Taiwan) [63] that uses a generic evolutionary approach (GA) and an empirical scoring function to explore the interaction modes between the hit molecules (**4e**, **8e**, and **9d**) and VEGFR-2/KDR (PDB ID: 3CJG) and EGFR (PDB ID: 4WKQ) as a biological targets. Firstly, the three-dimensional (3D) structures of VEGFR-2 with KIM complex and EGFR with gefitinib were obtained from RCSB Protein Data Bank (https://www.rcsb.org) and the co-crystallized ligand, was extracted and docked back to the corresponding binding site, to define the ability of docking protocol to reproduce the docking mode of the inhibitor observed in the crystal structure (*i*GEMDOCK validation) (Figure 8).

Secondly, the two-dimensional (2D) structure of hit compounds were drawn by ChemBioDraw Ultra 14.0 (PerkinElmer Informatics, Waltham, MA, USA) and converted to 3D structure by ChemBio3D Ultra 14.0 then saved as mol format after energy minimized and Molecular Dynamic (MD) performed using MMFF94 (Merck molecular force field) method [66]. Finally, the docking process has been done by uploading the protein pdb and ligand mol files to the *i*GEMDOCK program and the result has been analyzed with the Discovery Studio Visualizer Client 2020 (BIOVIA, San Diego, CA, USA). The docking study of compounds **4e**, **8e**, and **9d** toward VEGFR-2 and EGFR have been achieved in comparison with known inhibitors including KIM, gefitinib, sorafenib, and erlotinib (Figure 6). The fitness value (Table 5) is the total energy of a predicted pose in the binding site. The empirical scoring function of *i*GEMDOCK is estimated as [63]: Fitness = vdW + H-bond + Elec; where, the vdW term is pointed out to van der Waal energy. H-bond and Elec terms are denoted hydrogen bonding energy and electro statistic energy, respectively. As illustrated in Figure 8, the docked ligands (KIM and gefitinib) pretend the same binding mode as the crystal one and the Root Mean Square Distance (RMSD) was within the reliable range (≤2 Å), confirming the robustness of this approach.

## Conclusions

A simple and an effective methodology without addition of metal catalyst has been developed for mild construction of new coumarin analogous (**4a–f**, **5a,d–f**, **8a–e**, and **9a,d–f**) with high yield via domino amination-Knoevenagel condensation approach. All the synthesized compounds were characterized by various spectral methods. The anti-proliferative activity of hit compounds was performed using resazurin assay. Among them, compounds **4e** and **8e** that bearing 4-AMP unit displayed the best anti-cervical cancer activity with IC_50_ values of 15.5 ± 3.54 and 21 ± 4.24 μM, respectively. Similar way was observed with **4e** that showed the most optimistic cytotoxicity result toward A549 with the IC_50_ value of 12.94 ± 1.51 μM. Also, **9d** displayed a more significant impact of activity against PC3 with the IC_50_ value of 7.31 ± 0.48 μM. Moreover, the pooled results from the cellular anti-proliferative potency revealed that **8d** demonstrated selectivity against PC3 (IC_50_ = 20.16 ± 0.07 μM), while **8e** was selective toward KB-3-1 cell line with the IC_50_ value of 21 ± 4.24 μM. Enzymatic inhibitory activity disclosed that **8e** is a dual inhibitor of VEGFR-2 and EGFR with IC_50_ values of 24.67 and 31.6 nM, which were almost equipotent to sorafenib (31.08 nM) and erlotinib (26.79 nM), respectively. *In silico* studies showed that the estimated compounds (**4e**, **5e**, **8d,e**, and **9d**) passed all the drug-likeness metrics and they could be valuable lead compounds for further investigation in the future.

## Supplementary Material

SI

## Figures and Tables

**Table 1.** Optimization of domino amination-Knoevenagel condensation reaction to form **4a**^a^.

Entry	Solvent	Temp (°C)	Time (min)	Yield (%)^b^
1	EtOH	r.t.	40	65
2	DCM	r.t.	60	50
3	MeOH	r.t.	30	93
4	H_2_O	90	60	28

a Reaction conditions: **1a** (1.0 mmol, 1.0 equiv), **2a** (1.0 mmol, 1.0 equiv), and **3a** (2.5 mmol, 2.5 equiv) in solvent (2 mL) using open flask.

b Isolated yield.

**Table 2.** IC_50_ (μM) of all the synthesized compounds against KB-3-1(cervix), A549 (non-small cell lung), PC3 (prostate) human cancer cell lines.

Compounds	IC_50_ (μM)		
	KB-3-1^a^	A549^b^	PC3^b^
**4a**	—	>80	>80
**4b**	—	>80	>50
**4c**	>100	>80	>50
**4d**	>100	>80	>50
**4e**	**15.5 ± 3.54**	**12.94 ± 1.51**	**11.28 ± 0.06**
**4f**	—	>80	38.90*
**5a**	—	>80	>80
**5d**	>100	>50	31.12*
**5e**	>70	>80	>80
**5f**	—	>80	>80
**8a**	—	>80	>80
**8b**	—	>80	>80
**8c**	—	>80	>80
**8d**	>100	>80	**20.16 ± 0.07**
**8e**	**21 ± 4.24**	>50	>50
**9a**	>100	>80	>80
**9d**	>70	**19.78 ± 0.56**	**7.31 ± 0.48**
**9e**	—	>80	>80
**9f**	—	ND^c^	ND
(+)-Griseofulvin	19 ± 2.83	ND	ND
DMSO^d^	—	—	—

a Anti-cervical cancer activity was done at Organic and Bioorganic Chemistry, Faculty of Chemistry, Bielefeld University, Germany. IC_50_ values are the mean ± SD of two independent determinations, (+)-griseofulvin was used as positive control with IC_50_ = 19 ± 2.83 μM.

b Cytotoxicity IC_50_ values (mean ± SD of triplicate wells) against A549 (non-small lung) and PC3 (prostate) human cancer cell lines were tested at the Center for Pharmaceutical Research and Innovation, University of Kentucky, Lexington, USA, and obtained after 72 h incubation. Actinomycin D and H_2_O_2_ were used as positive control at 20 μM and 2 mM concentration, respectively (0% viable cells) without dose–response curves (IC_50_ not determined) and just utilized to make sure the cytotoxic assay is working [50–52].

c ND, not determined.

d Negative control; 0.1% dimethyl sulphoxide was used as negative control (100% live cells).

‘—’ Means no obvious inhibitory effect.

* Could not get +/− values due to the low toxicity.

**Table 3.** VEGFR-2 and EGFR inhibitory activities by compounds **4e**, **5e**, **8e**, and **9d** with their fold inactivation relative to the standard.

Compound	VEGFR-2		EGFR	
	IC_50_ (nM)	Fold to sorafenib	IC_50_ (nM)	Fold to erlotinib
**4e**	**36.76 ± 1.8**	1.2	67.09 ± 3.5	2.5
**5e**	88.08 ± 4.5	2.8	96.25 ± 4.7	3.6
**8e**	**24.67 ± 1.1**	0.8	**31.60 ± 1.5**	1.2
**9d**	**24.26 ± 1.1**	0.8	165.00 ± 8.0	6.2
Standard*	31.08 ± 1.8	1.0	26.79 ± 1.2	1.0

VEGFR-2, vascular endothelial growth factor receptor 2; EGFR, epidermal growth factor receptor.

* The standard is the sorafenib as inhibitor to VEGFR-2 enzyme while it is the erlotinib in the case of the EGFR.

**Table 4.** Physicochemical properties, drug-likeness, and medicinal chemistry parameters of compounds **4e**, **5e**, **8d**,**e**, and **9d**.

	Compounds				
Predictive models and their parameters	4e	5e	8d	8e	9d
MW (g/mol)	367.40	286.33	307.30	334.37	276.24
nROTB^a^ (≠)	6	4	2	4	2
Fraction Csp3	0.35	0.38	0.24	0.32	0.31
HBA^b^ (≠)	6	4	6	5	5
HBD^c^ (≠)	2	2	0	2	0
MR^d^	105.97	85.81	87.41	99.63	77.03
TPSA^e^ (Å^2^)	104.36	71.34	90.26	101.85	88.50
Log *P*_o/w_^f^ XLOGP3	2.14	2.15	1.46	2.01	1.42
WLOGP	1.60	1.45	1.57	1.95	1.16
MLOGP	1.18	1.20	0.47	0.94	0.37
Log *S*^g^ (ESOL)	−3.34	−3.06	−2.85	−3.21	−2.69
Quantitative solubility (mg/mL)	1.66e–01	2.50e–01	4.29e–01	2.06e–01	5.70e–01
Qualitative solubility	Soluble	Soluble	Soluble	Soluble	Soluble
Lipinski (Pfizer filter, RO5)^h^	Yes	Yes	Yes	Yes	Yes
Ghose^i^ (Amgen filter)	Yes	Yes	Yes	Yes	Yes
Veber^j^ (GSK filter)	Yes	Yes	Yes	Yes	Yes
Egan^k^ (Pharmacia filter)	Yes	Yes	Yes	Yes	Yes
Muegge^l^ (Bayer filter)	Yes	Yes	Yes	Yes	Yes
Bioavailability score	0.55	0.55	0.55	0.55	0.55
PAINS	0 alert	0 alert	0 alert	0 alert	0 alert
Lead-likeness (RO3)^m^	No; 1 violation: MW>350	Yes	Yes	Yes	Yes
Target prediction	Kinase 73.3%	Kinase 53.3%	Kinase 26.7%	Nuclear receptor 20.0%	Protease 33.3%

a nROTB, no. of rotatable bonds.

b HBA, hydrogen bond acceptor.

c HBD, hydrogen bond donor.

d MR, molar refractivity.

e TPSA, topological polar surface area.

f Log *P*_o/w_, the partition coefficient between *n*-octanol and water.

g Log *S*, the decimal logarithm of the molar solubility in water.

h Lipinski (RO5) criteria range are: lipophilicity (Log *P*_o/w_) ≤ 5, MW ≤ 500, H-bond donors ≤ 5, and H-bond acceptors ≤ 10.

i Ghose filter criteria range are: Log *P*_o/w_ in −0.4 to +5.6 range, MR from 40 to 130, MW from 180 to 480, no. of atoms from 20 to 70.

j Veber rule criteria range are: RB ≤ 10 and TPSA ≤ 140 Å^2^.

k Egan rule criteria range are: WLOGP ≤ 5.88, TPSA ≤ 131.6 Å^2^.

l Muegge rule criteria range are: 200 ≤ MW ≤ 600, −2 ≤ XLOGP3 ≤ 5, TPSA = 150 Å^2^, no. of rings ≤ 7, no. of heteroatoms > 1, nROTB ≤ 15, HBA ≤ 10, HBD ≤ 5.

m RO3 criteria range are: XLOGP3 ≤ 3.5, MW ≤ 350, H-bond donors ≤ 3, H-bond acceptors ≤ 3, and RB ≤ 3 [57].

**Table 5.** Binding fitness and no. of H-bonds of **4e**, **8e**, and **9d** to VEGFR-2 and EGFR in comparison with KIM, sorafenib, gefitinib, and erlotinib as the reference drugs.

Compounds	Fitness (kcal/mol)		H-bonds (≠)	
	VEGFR-2	EGFR	VEGFR-2	EGFR
**4e**	−104.204	−112.020	2	2
**8e**	−100.502	−113.558	2	2
**9d**	−89.051	−94.544	4	2
KIM	−102.401	—	2	—
Sorafenib	−107.572	—	5	—
Gefitinib	—	−104.299	—	1
Erlotinib	−	−102.323	—	3
