# Supplementary material for: Metal-free domino amination-Knoevenagel condensation approach to access new coumarins as potent nanomolar inhibitors of VEGFR-2 and EGFR
Source: Green Chem Lett Rev. Author manuscript; Available in PMC 2022 Jul 11. (PMC9273165; doi:10.1080/17518253.2021.1981462)
Supplement: SI [file NIHMS1761482-supplement-SI.pdf]

Supporting Information

Metal-Free Domino Amination-Knoevenagel Condensation Approach to Access New Coumarins as Potent Nanomolar Inhibitors of VEGFR-2 and EGFR

Essam M. Eliwa[a,b *], Marcel Frese[a], Ahmed H. Halawa[b], Maha M soltan[c], Larissa V. Ponomareva[d,e], Jon S. Thorson[d,e], Khaled A. Shaaban[d,e], Mohamed Shaaban[a,f], Ahmed M. El-Agrody[b *] & Norbert Sewald[a]

[a] *Organic and Bioorganic Chemistry, Faculty of Chemistry, Bielefeld University, Bielefeld D-33501, Germany*

[b] *Chemistry Department, Faculty of Science, Al-Azhar University, Nasr City-Cairo 11884, Egypt*

[c] *Biology Unit, Central Laboratory for Pharmaceutical and Drug Industries Research Division, Chemistry of Medicinal Plants Department, Pharmaceutical and Drug Industries Research Division, National Research Centre, El Buhouth St. 33 Dokki-Giza Cairo 12622, Egypt*

[d] *Center for Pharmaceutical Research and Innovation, University of Kentucky, Lexington, Kentucky 40536, United States*

[e] *Department of Pharmaceutical Sciences, College of Pharmacy, University of Kentucky, Lexington, Kentucky 40536, United States*

[f] *Chemistry of Natural Compounds Department, Pharmaceutical and Drug Industries Research Division, National Research Centre, El-Behoos St. 33, Dokki-Cairo 12622, Egypt*

[*] Corresponding authors (emails: essameliwa85@azhar.edu.eg; elagrody_am@azhar.edu.eg)

Table of Contents

Figures	Page No.
**Figure S1**. ^{1}H NMR (500 MHz, DMSO-d_6) of **4a**	4
**Figure S2**. ^{13}C NMR (125 MHz, DMSO-d_6) of **4a**	4
**Figure S3**. (+)-ESI-MS of **4a**	5
**Figure S4**. ^{1}H NMR (500 MHz, DMSO-d_6) of **4b**	5
**Figure S5**. ^{13}C NMR (125 MHz, DMSO-d_6) of **4b**	6
**Figure S6**. (+)-ESI-MS of **4b**	6
**Figure S7**. ^{1}H NMR (500 MHz, DMSO-d_6) of **4c**	7
**Figure S8**. ^{13}C NMR (125 MHz, DMSO-d_6) of **4c**	7
**Figure S9**. (+)-ESI-MS of **4c**	8
**Figure S10**. ^{1}H NMR (500 MHz, DMSO-d_6) of **4d**	8
**Figure S11**. ^{13}C NMR (125 MHz, DMSO-d_6) of **4d**	9
**Figure S12**. (+)-ESI-MS of **4d**	9
**Figure S13**. ^{1}H NMR (500 MHz, DMSO-d_6) of **4e**	10
**Figure S14**. ^{13}C NMR (125 MHz, DMSO-d_6) of **4e**	10
**Figure S15**. (+)-ESI-MS of **4e**	11
**Figure S16**. ^{1}H NMR (500 MHz, DMSO-d_6) of **4f**	11
**Figure S17**. ^{13}C NMR (125 MHz, DMSO-d_6) of **4f**	12

Figure S18. (+)-ESI-MS of **4f** 12
Figure S19. ^{1}H NMR (500 MHz, DMSO-d_6) of **8a** 13
Figure S20. ^{13}C NMR (125 MHz, DMSO-d_6) of **8a** 13
Figure S21. (+)-ESI-MS of **8a** 14
Figure S22. ^{1}H NMR (500 MHz, DMSO-d_6) of **8b** 14
Figure S23. ^{13}C NMR (125 MHz, DMSO-d_6) of **8b** 15
Figure S24. (+)-ESI-MS of **8b** 15
Figure S25. ^{1}H NMR (500 MHz, DMSO-d_6) of **8c** 16
Figure S26. ^{13}C NMR (125 MHz, DMSO-d_6) of **8c** 16
Figure S27. (+)-ESI-MS of **8c** 17
Figure S28. (+)-ESI-MS of **8d** 17
Figure S29. ^{1}H NMR (500 MHz, DMSO-d_6) of **8e** 18
Figure S30. ^{13}C NMR (125 MHz, DMSO-d_6) of **8e** 18
Figure S31. (+)-ESI-MS of **8e** 19
Figure S32. ^{1}H NMR (500 MHz, DMSO-d_6) of **5a** 19
Figure S33. ^{13}C NMR (125 MHz, DMSO-d_6) of **5a** 20
Figure S34. (+)-ESI-MS of **5a** 20
Figure S35A. ^{1}H NMR (500 MHz, DMSO-d_6) of **5d** 21
Figure S35B. ^{1}H NMR (500 MHz, DMSO-d_6) of **5d** 21
Figure S36. ^{13}C NMR (125 MHz, DMSO-d_6) of **5d** 22
Figure S37. (+)-ESI-MS of **5d** 22
Figure S38A. ^{1}H NMR (500 MHz, DMSO-d_6) of **5e** 23
Figure S38B. ^{1}H NMR (500 MHz, DMSO-d_6) of **5e** 23
Figure S39. ^{13}C NMR (125 MHz, DMSO-d_6) of **5e** 24
Figure S40. (+)-ESI-MS of **5e** 24
Figure S41A. ^{1}H NMR (500 MHz, DMSO-d_6) of **5f** 25
Figure S41B. ^{1}H NMR (500 MHz, DMSO-d_6) of **5f** 25
Figure S42. ^{13}C NMR (125 MHz, DMSO-d_6) of **5f** 26
Figure S43. (+)-ESI-MS of **5f** 26
Figure S44A. ^{1}H NMR (500 MHz, DMSO-d_6) of **9a** 27
Figure S44B. ^{1}H NMR (500 MHz, DMSO-d_6) of **9a** 27
Figure S45. ^{13}C NMR (125 MHz, DMSO-d_6) of **9a** 28
Figure S46. (+)-ESI-MS of **9a** 28
Figure S47A. ^{1}H NMR (500 MHz, DMSO-d_6) of **9d** 29
Figure S47B. ^{1}H NMR (500 MHz, DMSO-d_6) of **9d** 29
Figure S48. ^{13}C NMR (125 MHz, DMSO-d_6) of **9d** 30

**Figure S49**. (+)-ESI-MS of **9d**	30
**Figure S50**. ^{1}H NMR (500 MHz, DMSO-d_6) of **9e**	31
**Figure S51**. ^{13}C NMR (125 MHz, DMSO-d_6) of **9e**	31
**Figure S52**. (+)-ESI-MS of **9e**	32
**Figure S53**. ^{1}H NMR (500 MHz, DMSO-d_6) of **9f**	32
**Figure S54**. ^{13}C NMR (125 MHz, DMSO-d_6) of **9f**	33
**Figure S55**. (+)-ESI-MS of **9f**	33

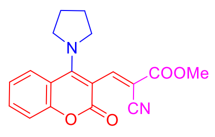

4a

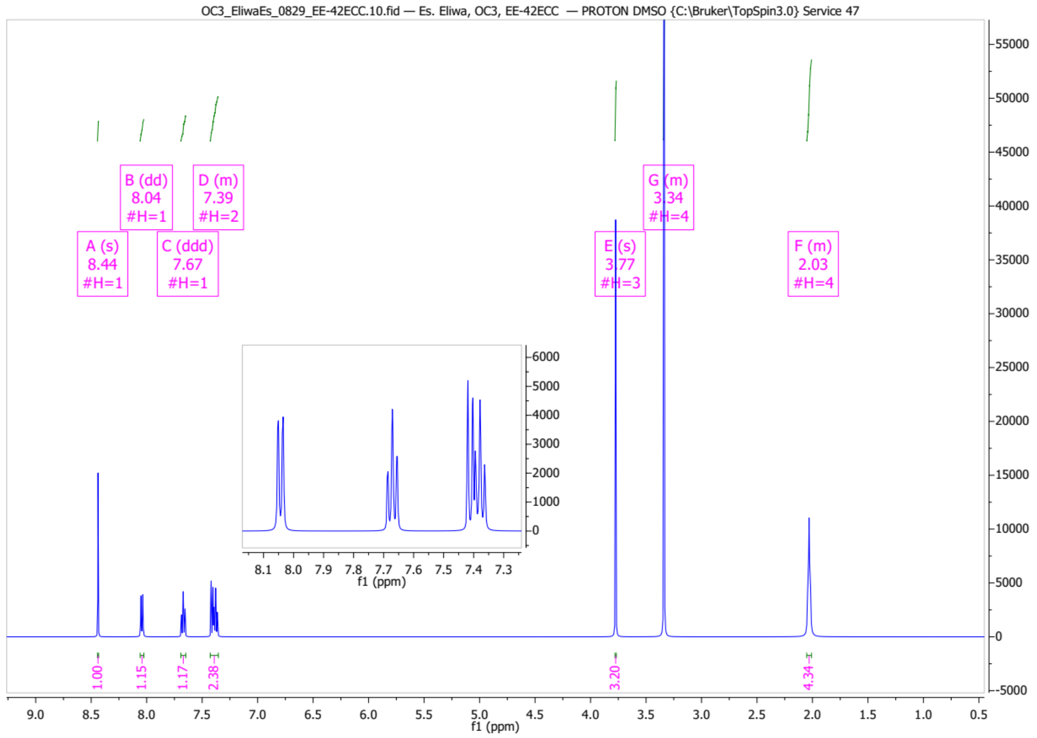

Figure S1. ^{1}H NMR (500 MHz, DMSO-d_6) of **4a**.

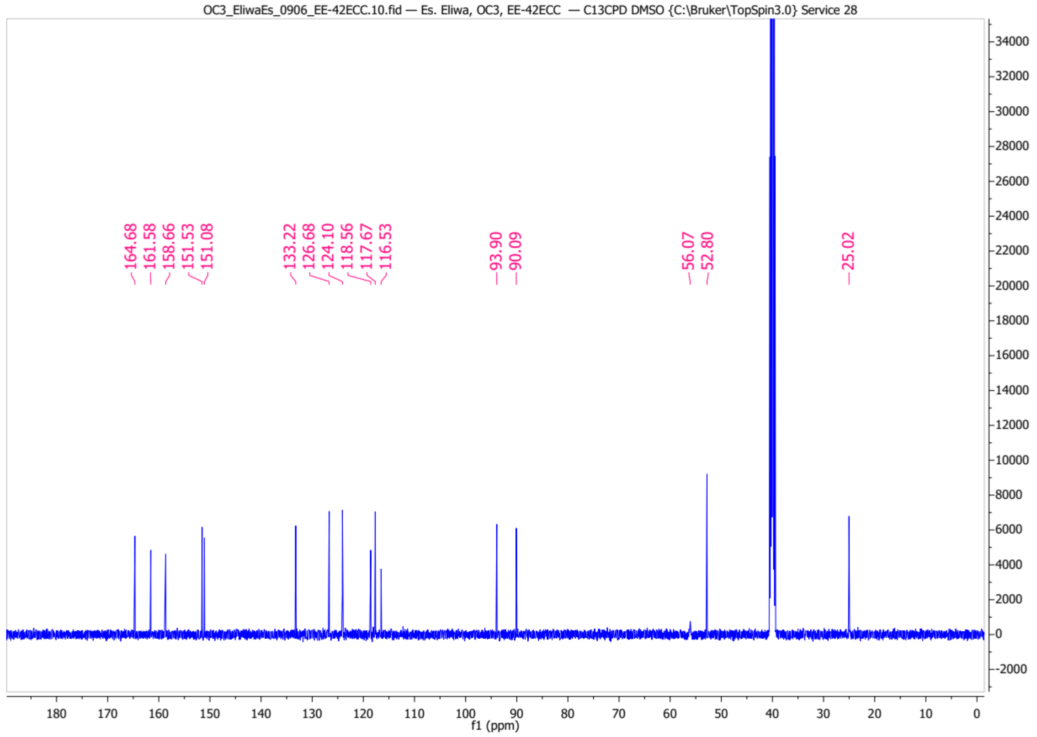

Figure S2. ^{13}C NMR (125 MHz, DMSO-d_6) of **4a**

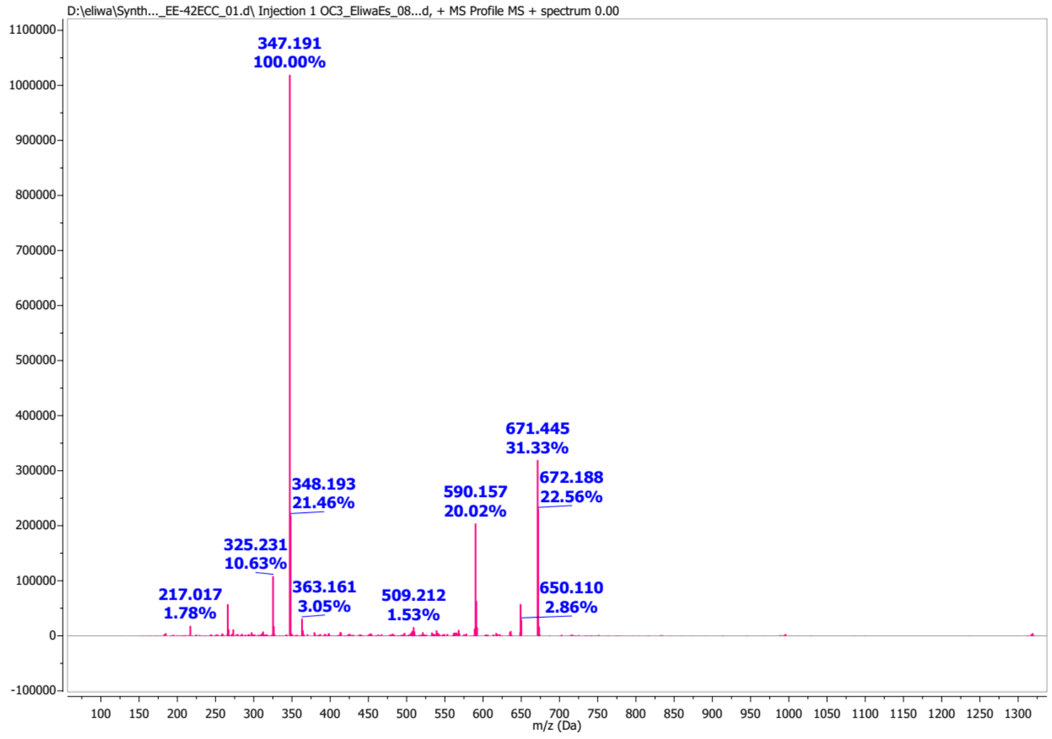

Figure S3. (+)-ESI-MS of **4a**

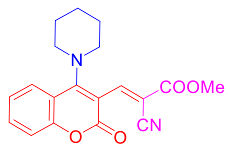

4b

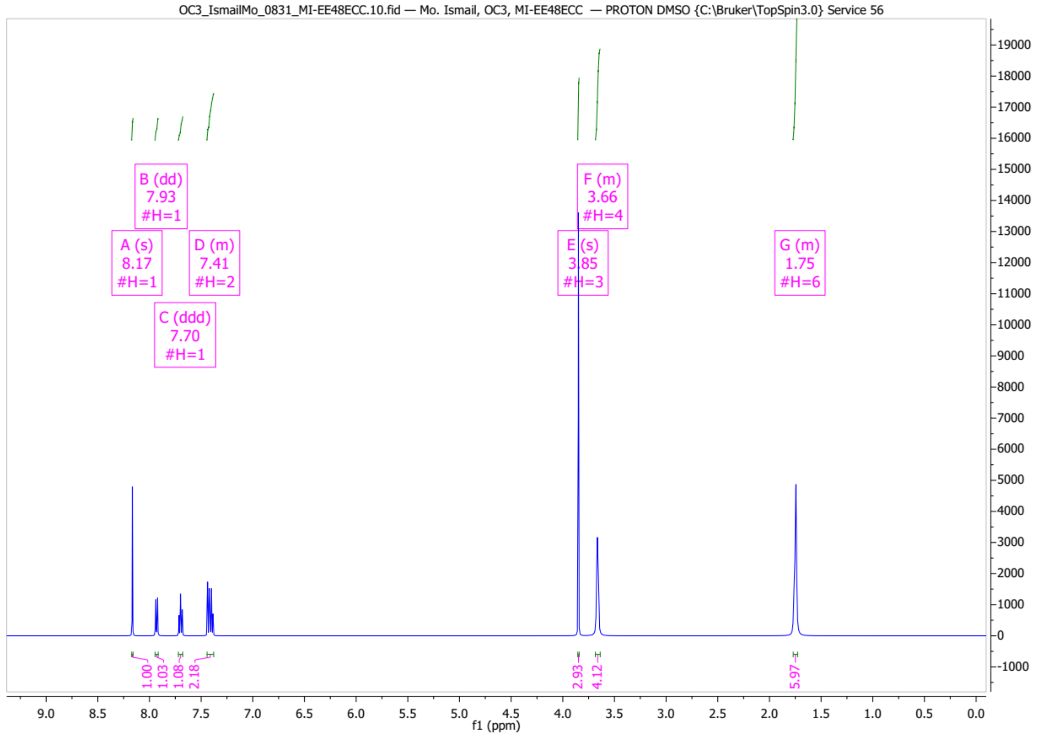

Figure S4. ^{1}H NMR (500 MHz, DMSO-d_6) of **4b**

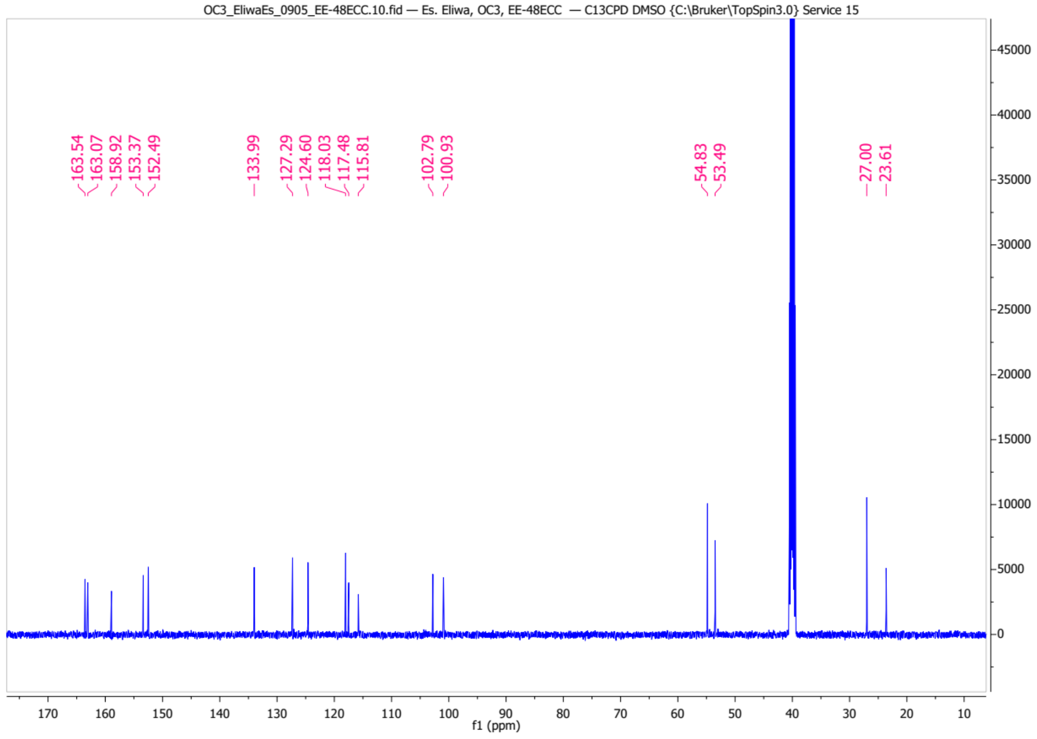

Figure S5. ^{13}C NMR (125 MHz, DMSO-d_6) of **4b**

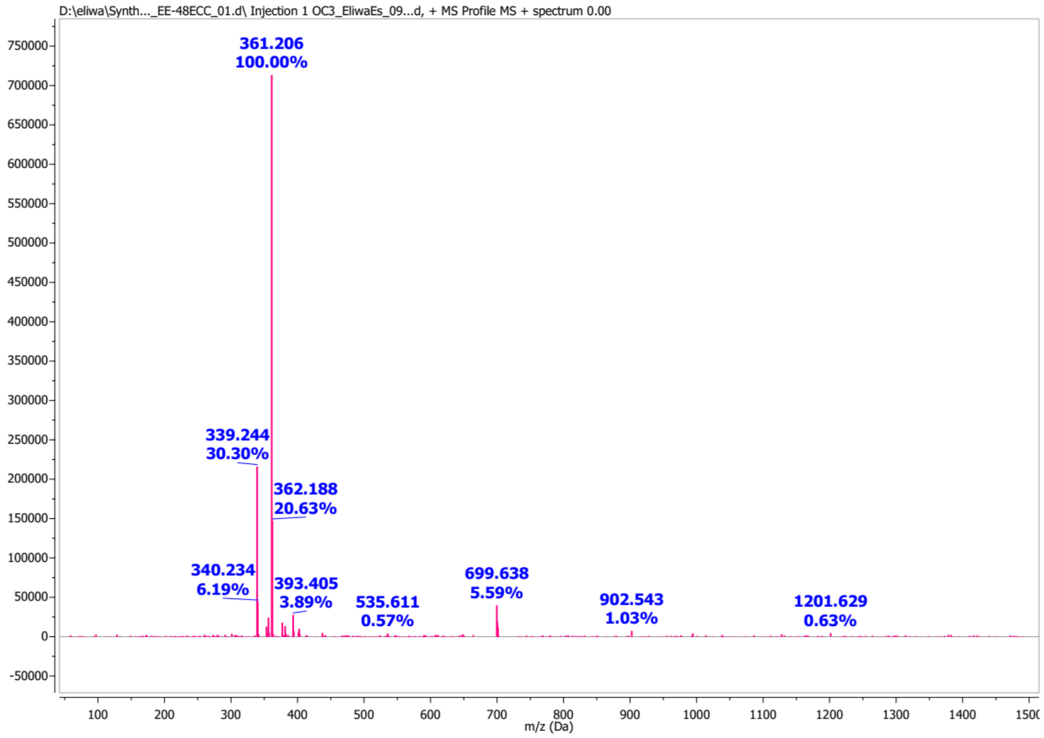

Figure S6. (+)-ESI-MS of **4b**

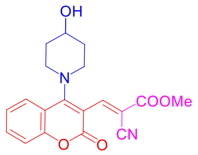

4c

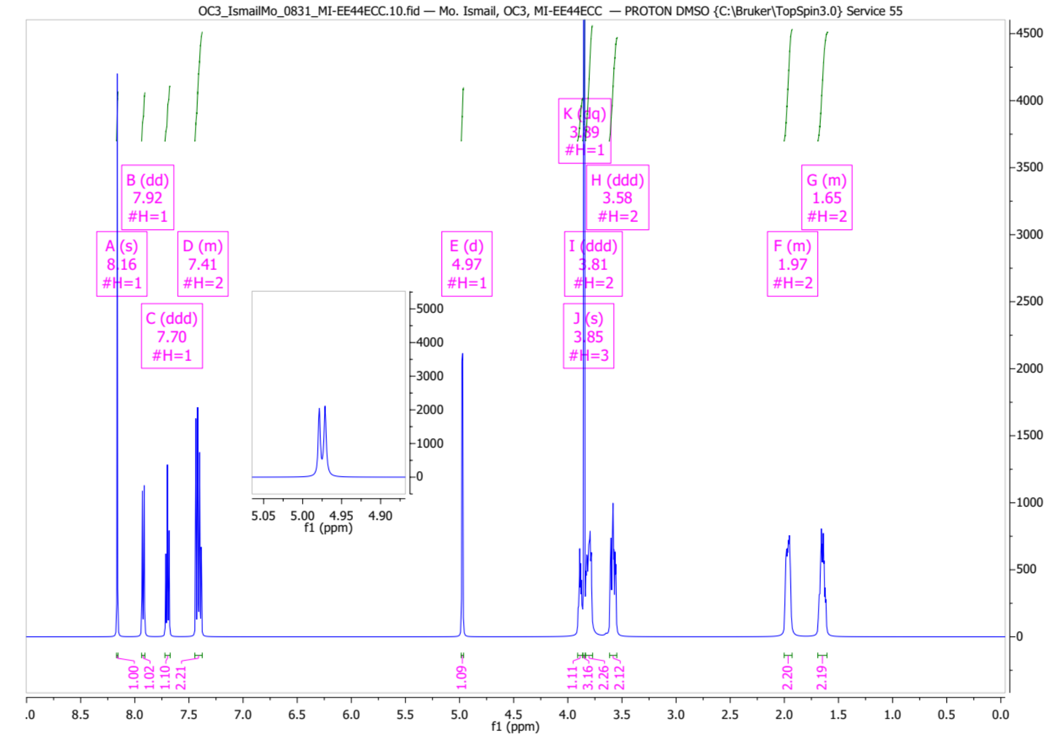

Figure S7. ^{1}H NMR (500 MHz, DMSO-d_6) of **4c**

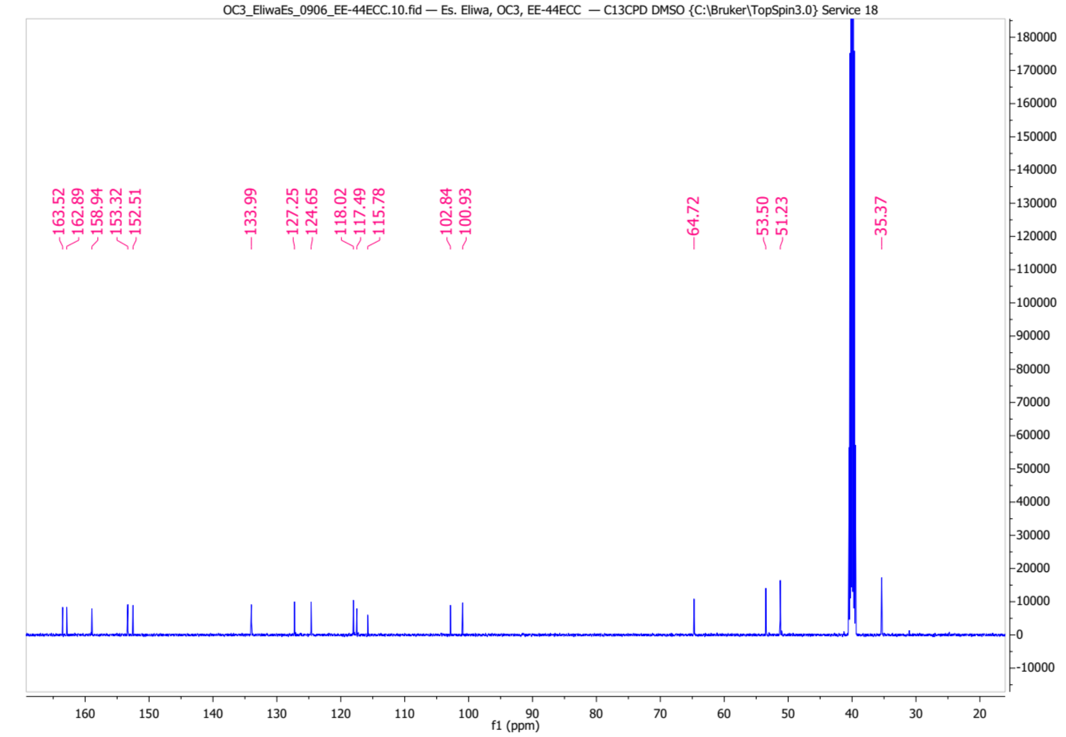

Figure S8. ^{13}C NMR (125 MHz, DMSO-d_6) of **4c**

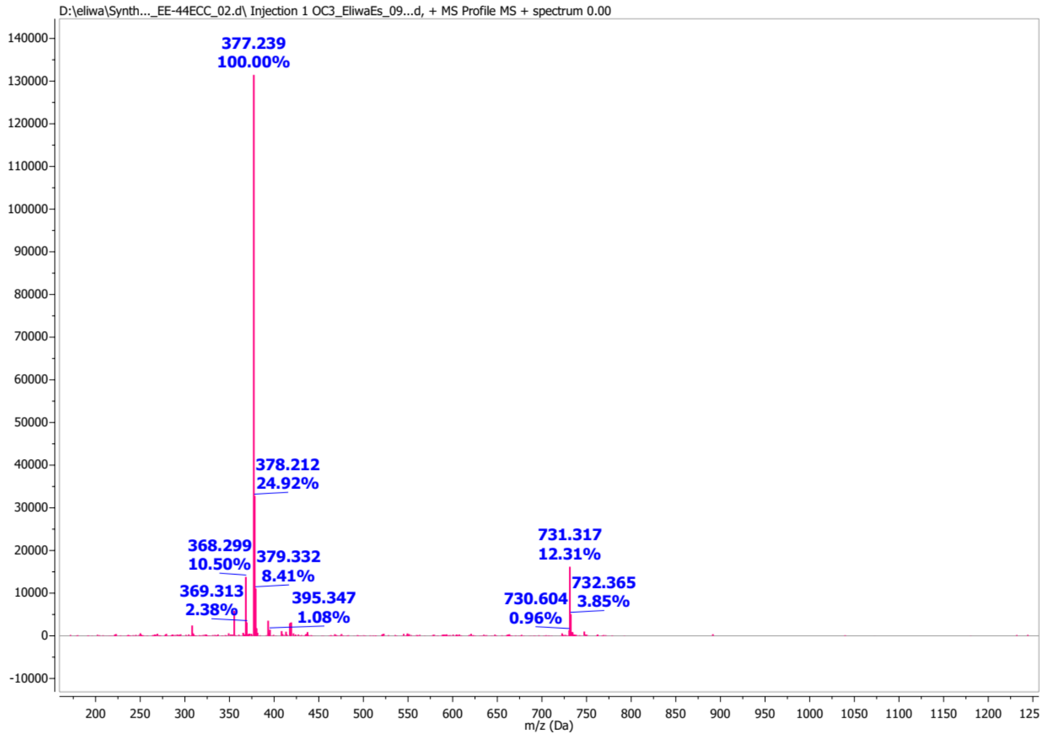

Figure S9. (+)-ESI-MS of **4c**

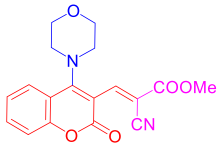

4d

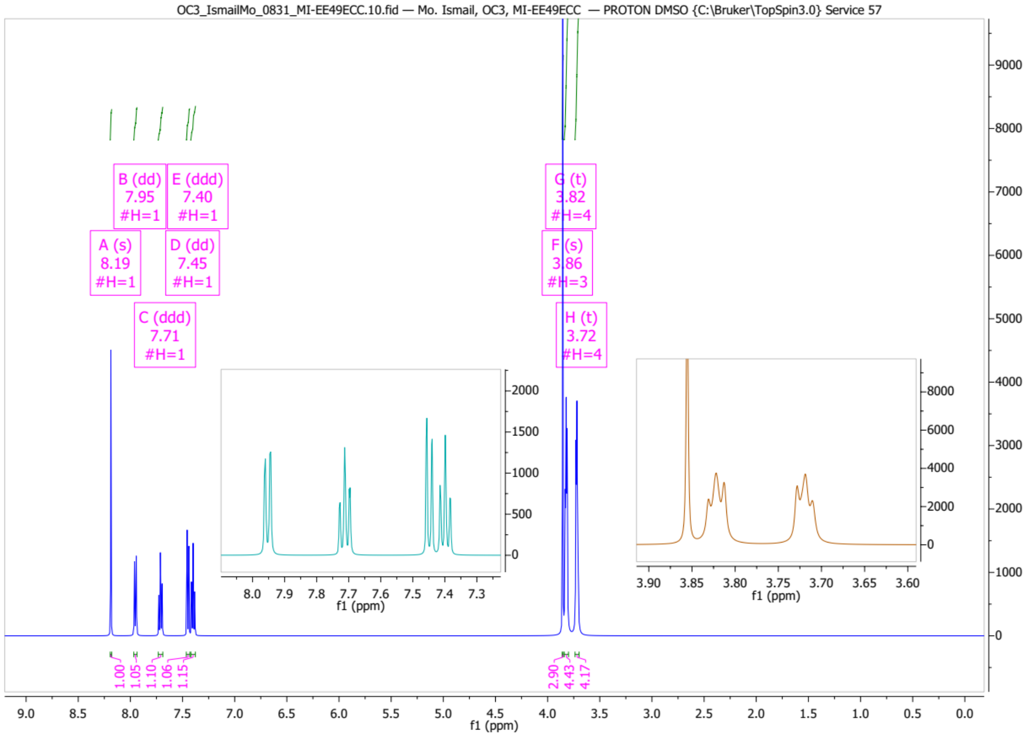

Figure S10. ^{1}H NMR (500 MHz, DMSO-d_6) of **4d**

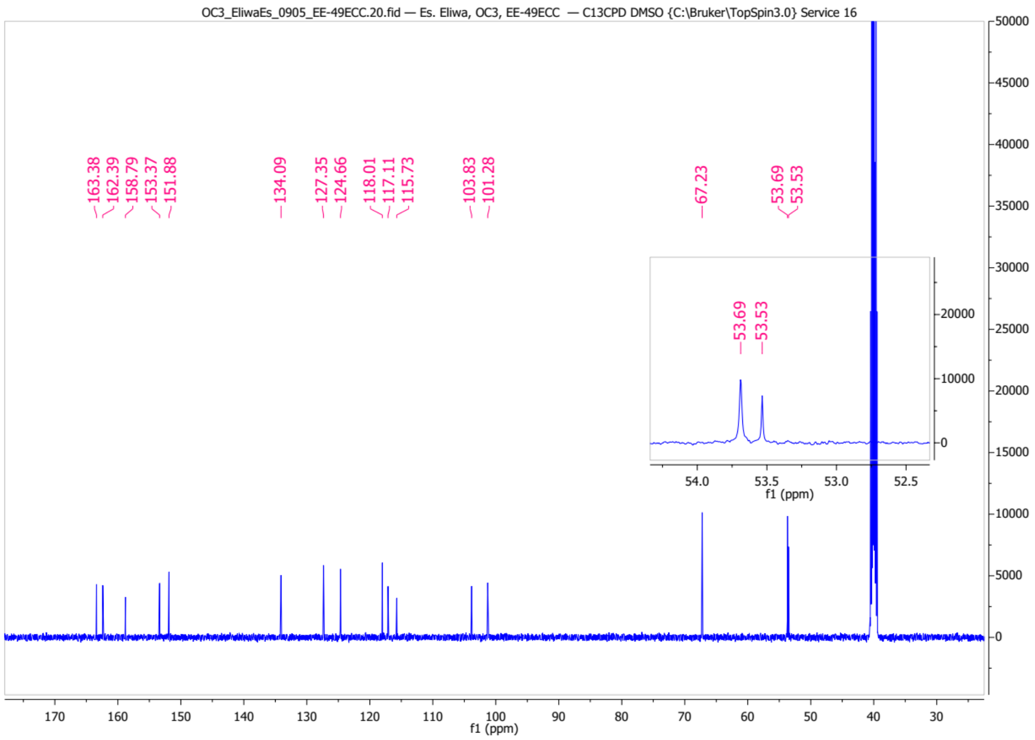

Figure S11. ^{13}C NMR (125 MHz, DMSO-d_6) of **4d**

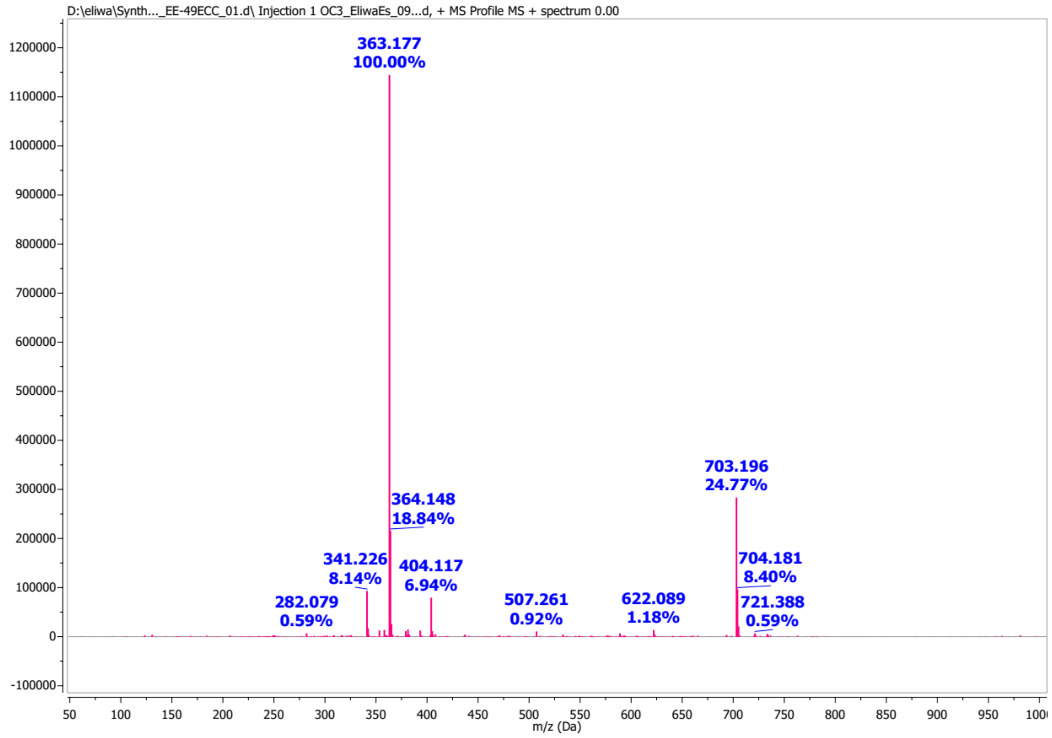

Figure S12. (+)-ESI-MS of **4d**

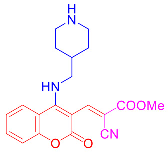

4e

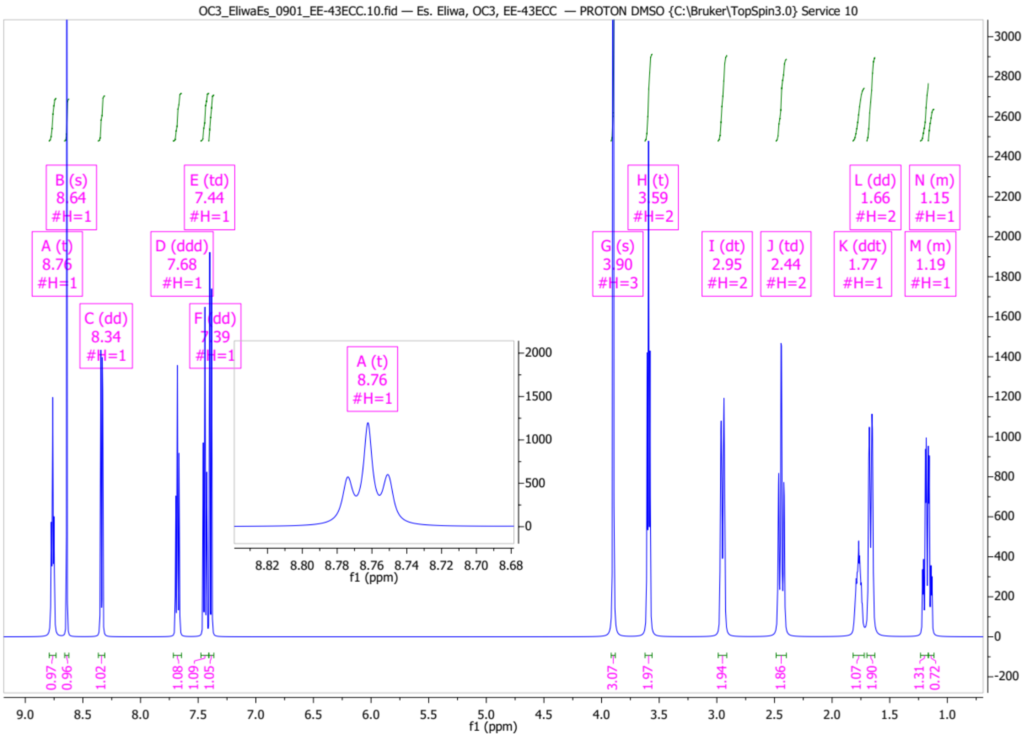

Figure S13. ^{1}H NMR (500 MHz, DMSO-d_6) of **4e**

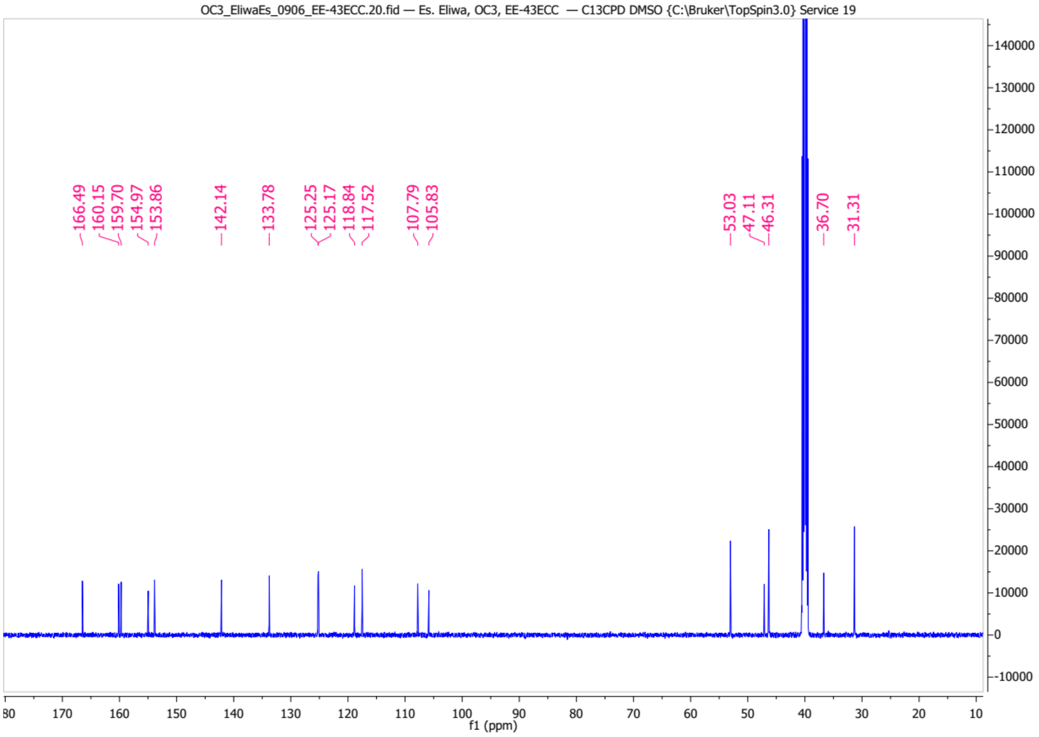

Figure S14. ^{13}C NMR (125 MHz, DMSO-d_6) of **4e**

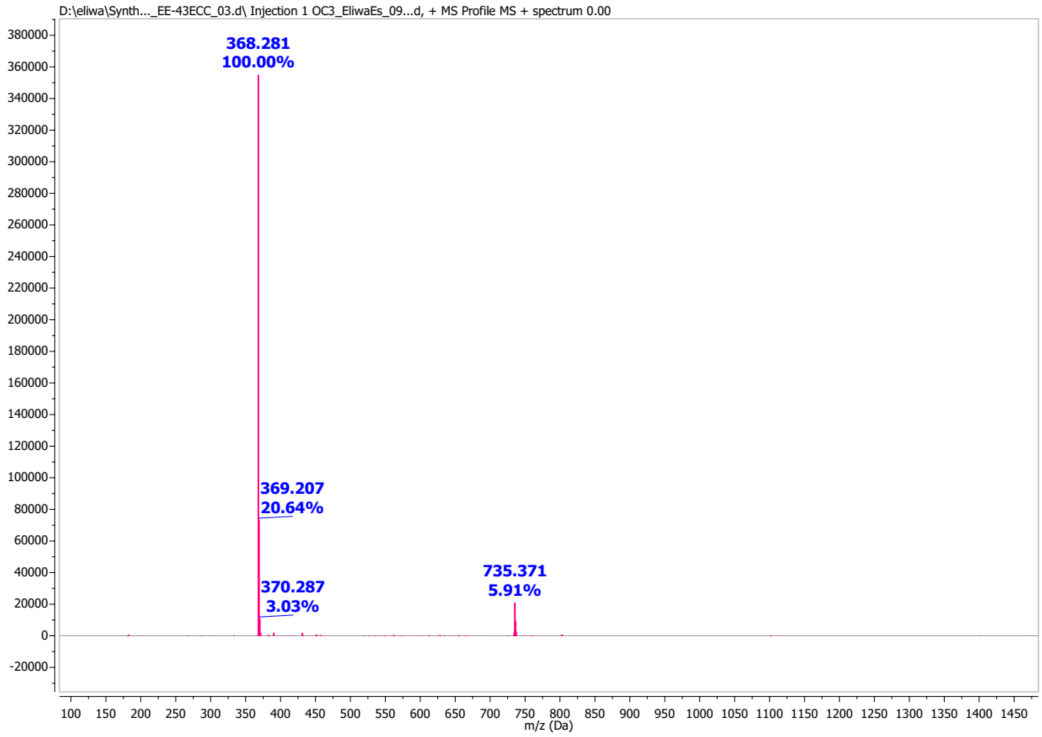

Figure S15. (+)-ESI-MS of **4e**

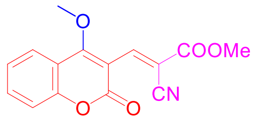

4f

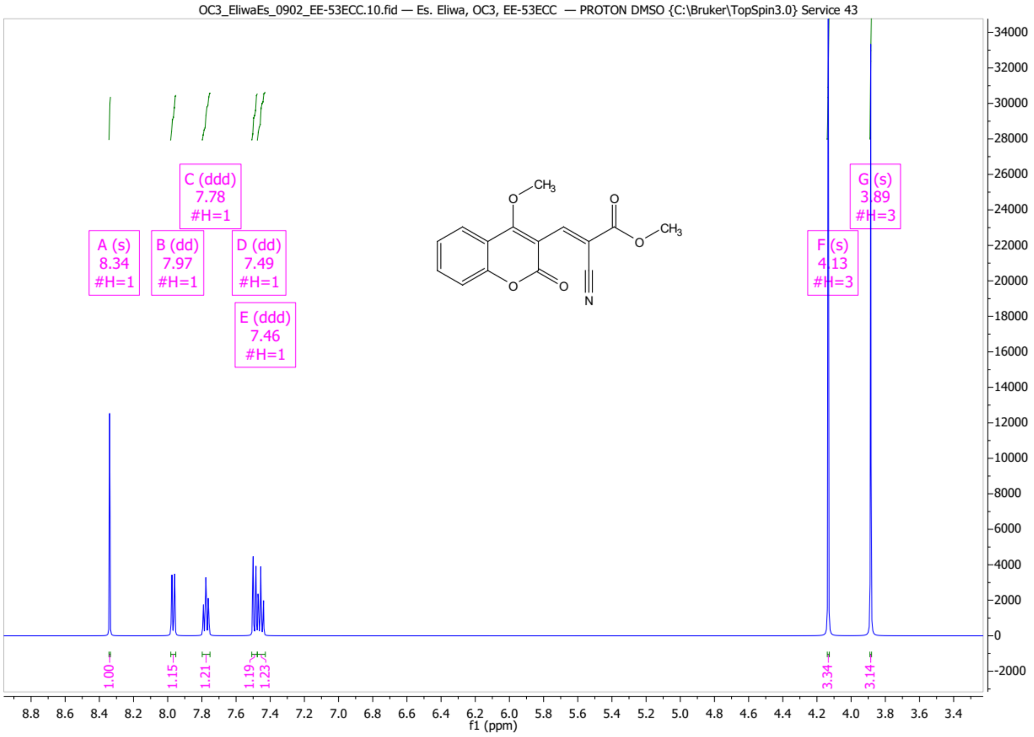

Figure S16. ^{1}H NMR (500 MHz, DMSO-d_6) of **4f**

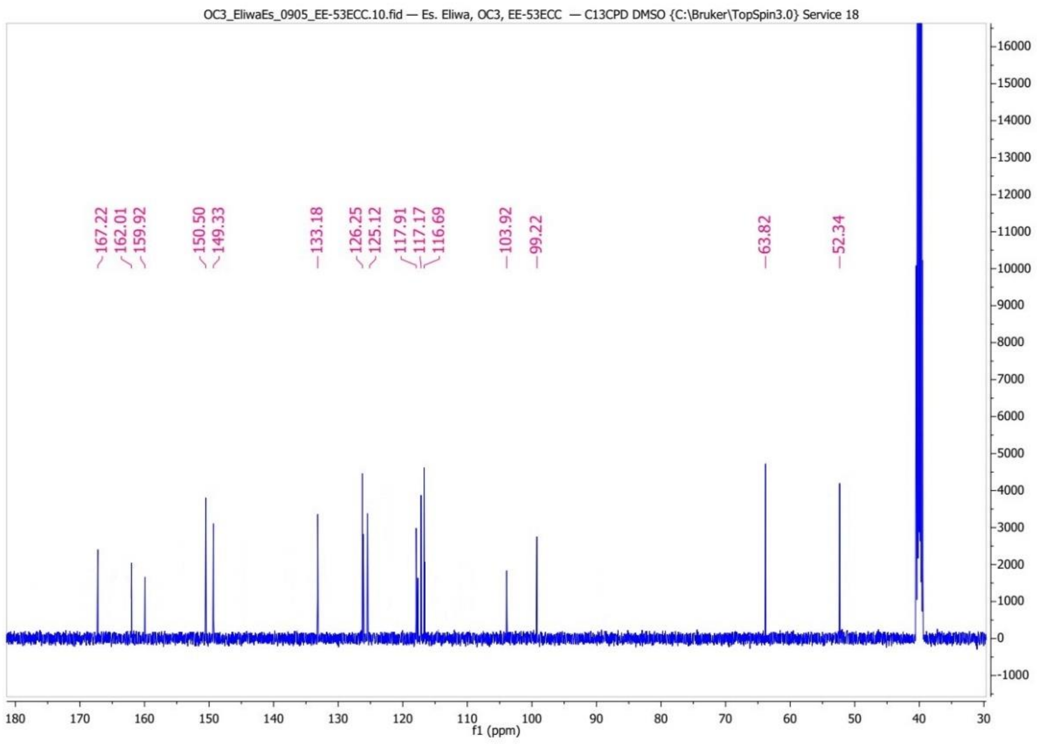

Figure S17. ^{13}C NMR (125 MHz, DMSO-d_6) of **4f**

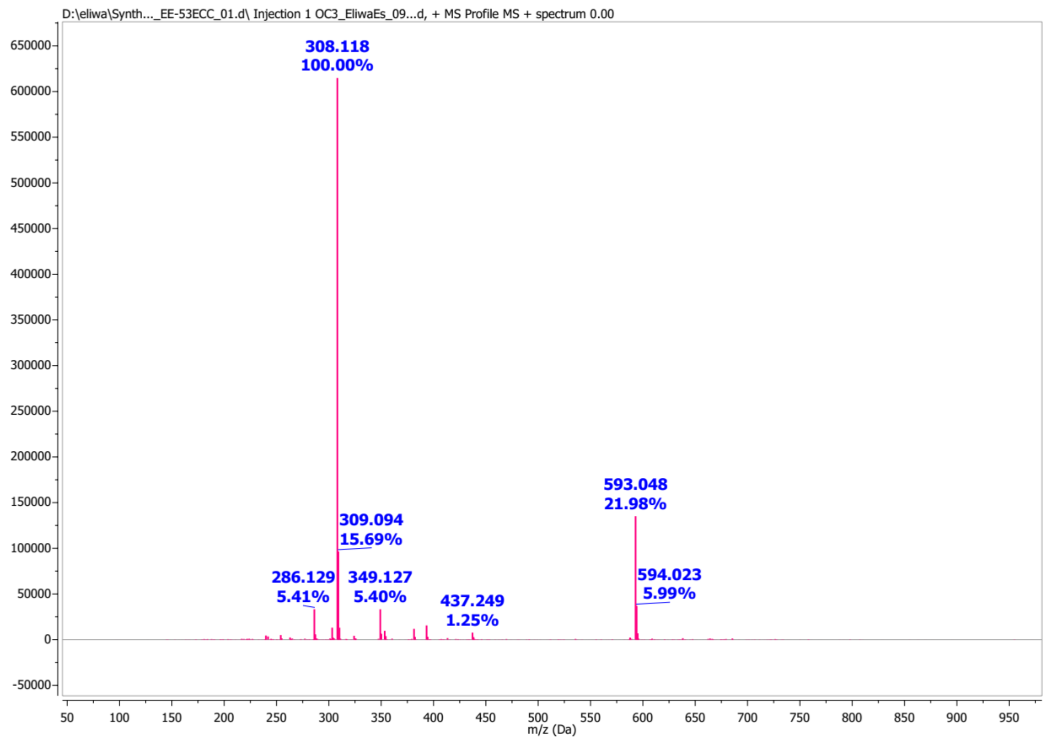

Figure S18. (+)-ESI-MS of **4f**

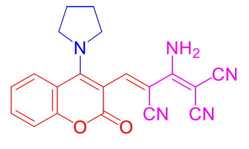

8a

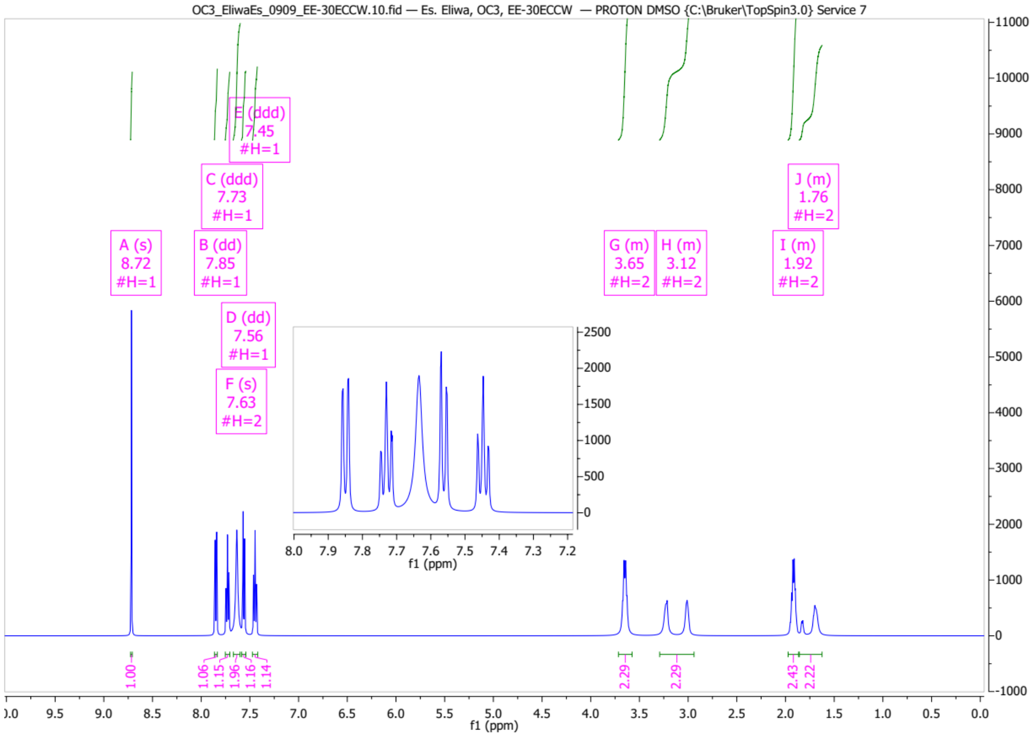

Figure S19. ^{1}H NMR (500 MHz, DMSO-d_6) of **8a**

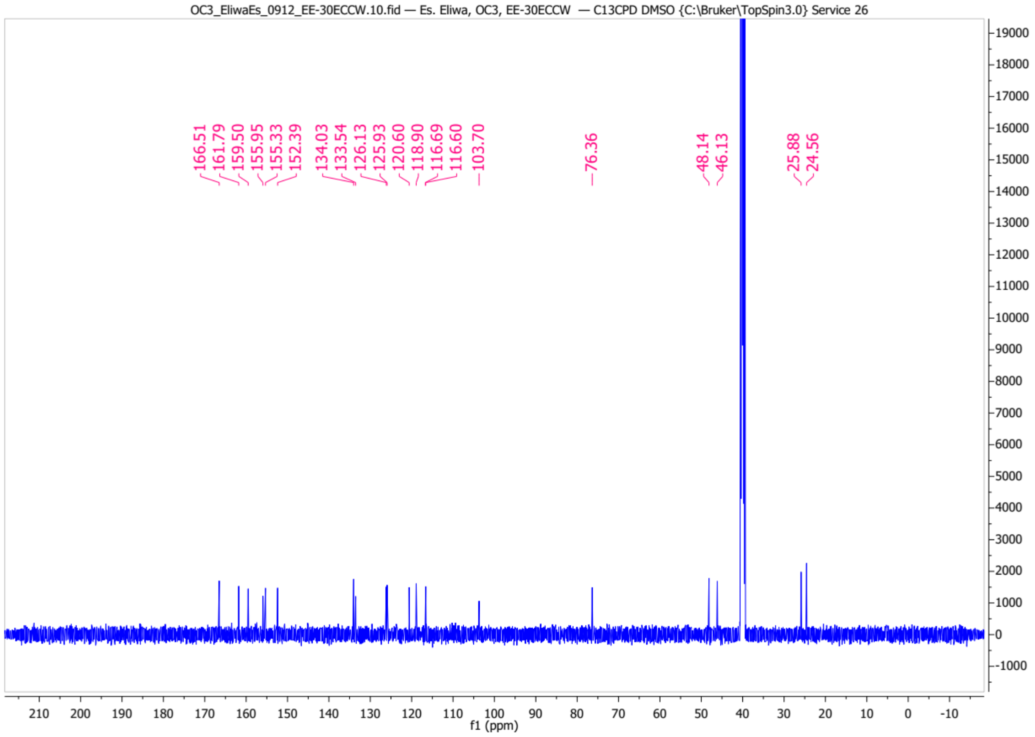

Figure S20. ^{13}C NMR (125 MHz, DMSO-d_6) of **8a**

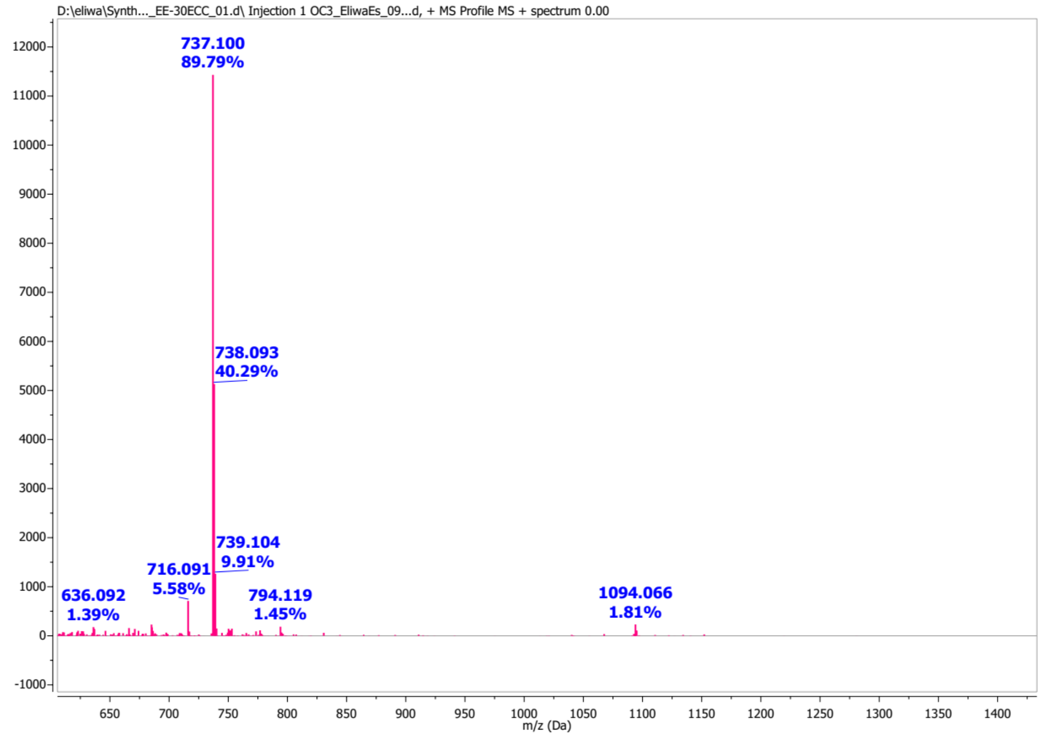

Figure S21. (+)-ESI-MS of **8a**

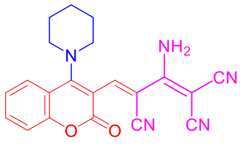

8b

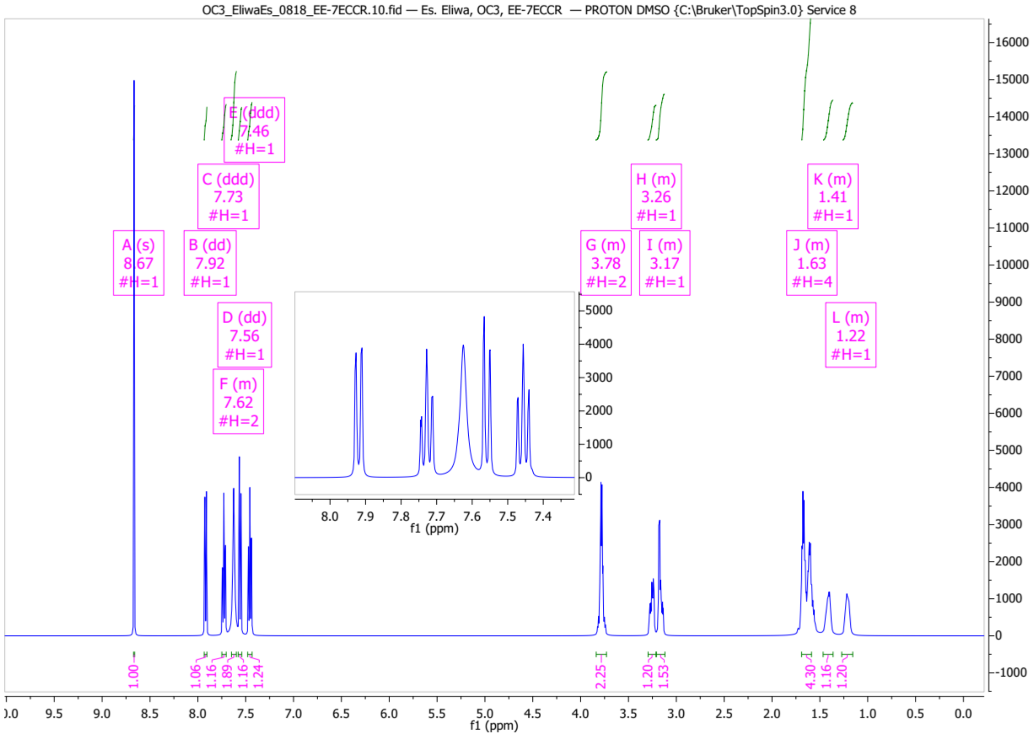

Figure S22. ^{1}H NMR (500 MHz, DMSO-d_6) of **8b**

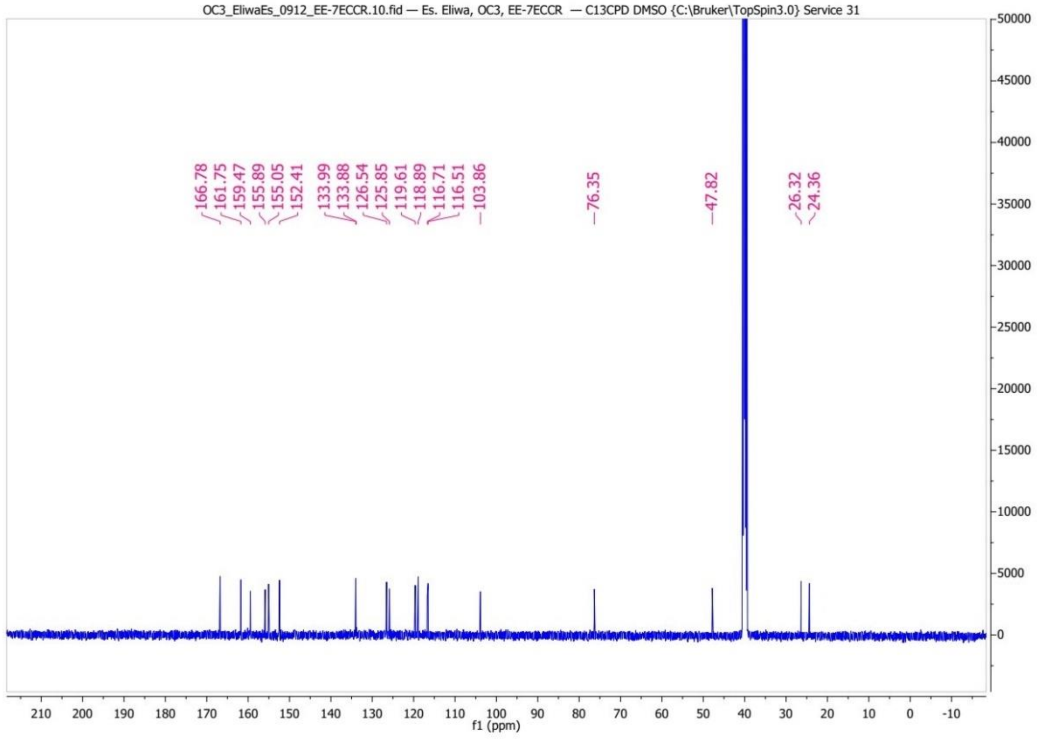

Figure S23. ^{13}C NMR (125 MHz, DMSO-d_6) of **8b**

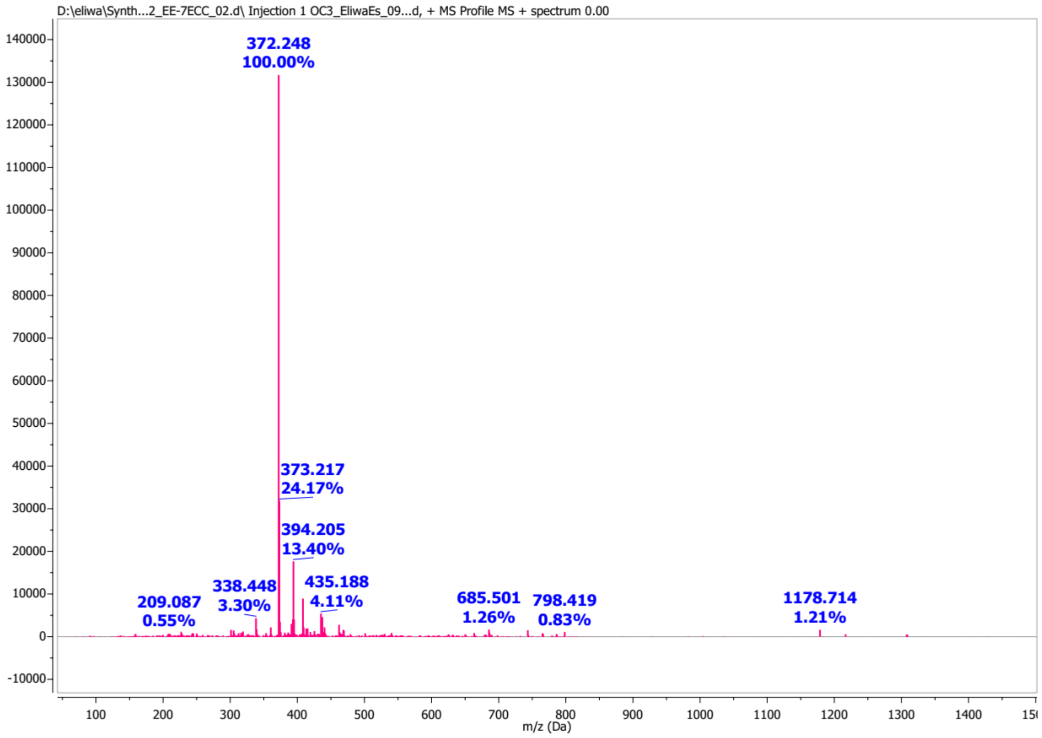

Figure S24. (+)-ESI-MS of **8b**

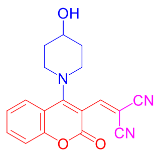

8c

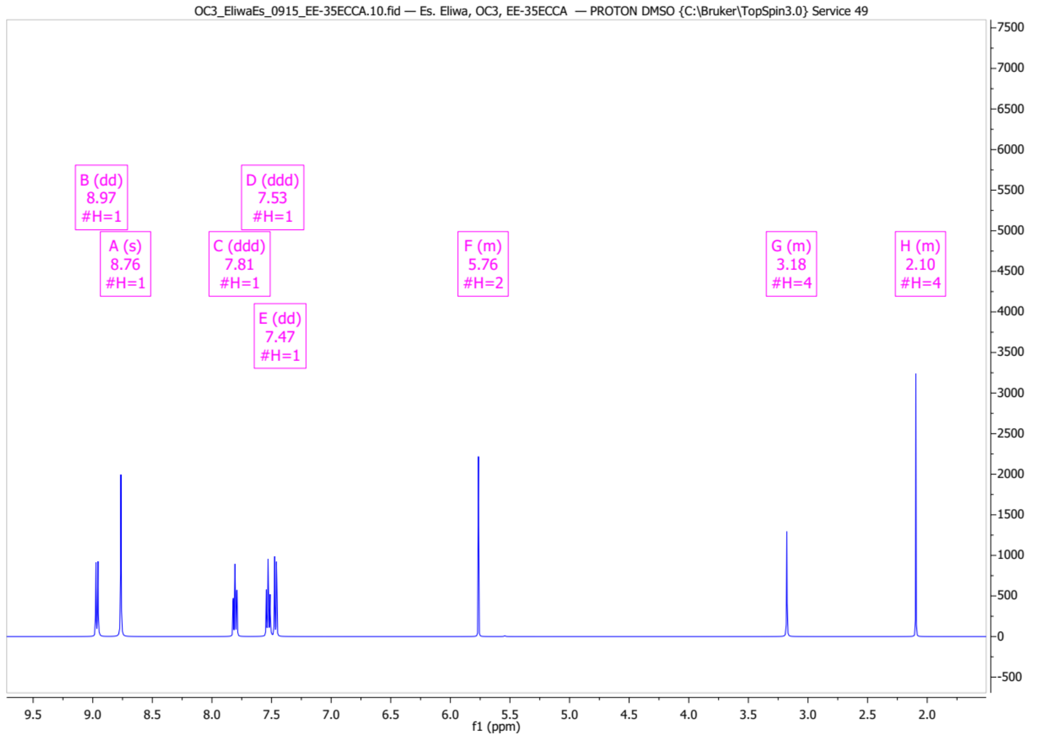

Figure S25. ^{1}H NMR (500 MHz, DMSO-d_6) of **8c**

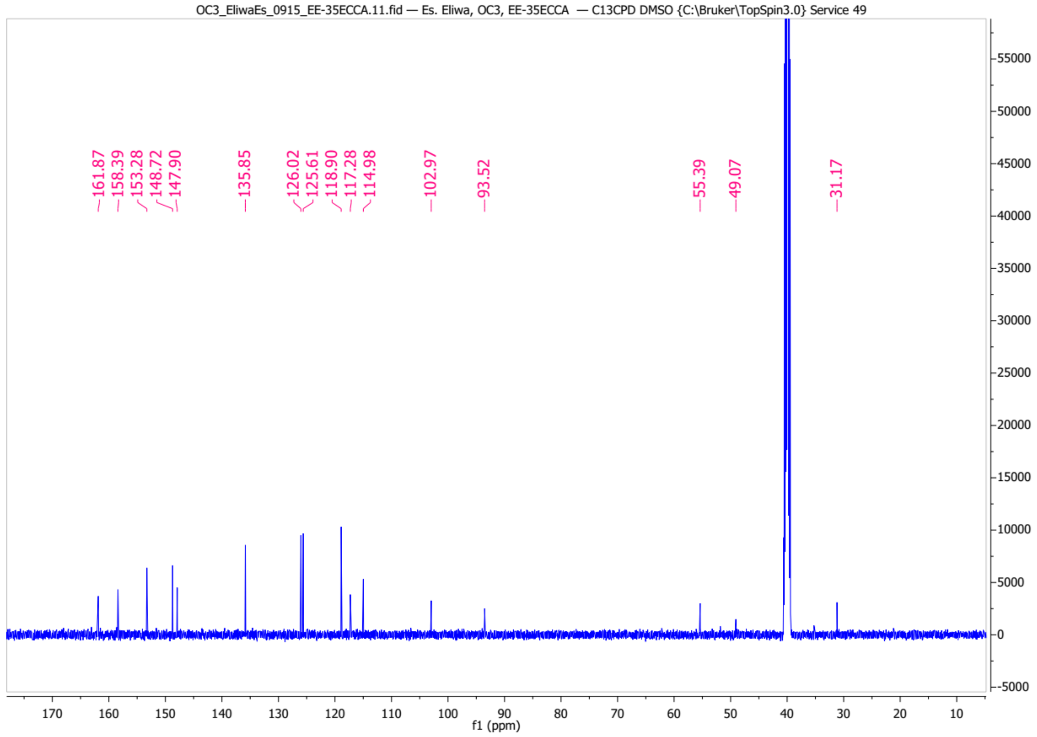

Figure S26. ^{13}C NMR (125 MHz, DMSO-d_6) of **8c**

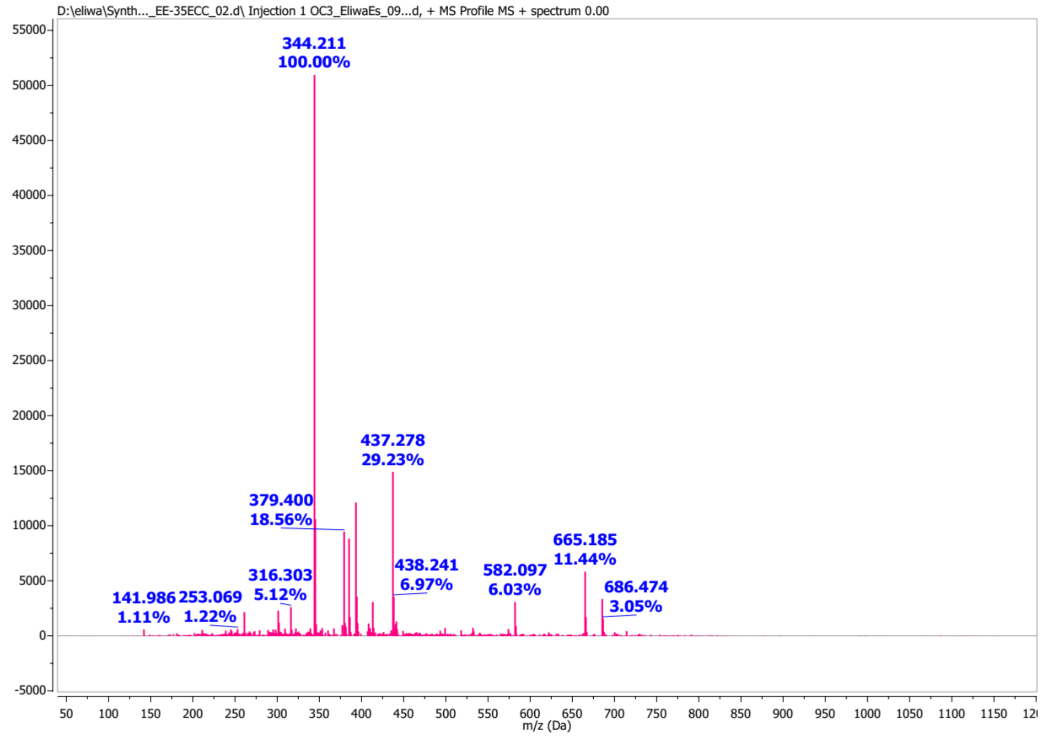

Figure S27. (+)-ESI-MS of **8c**

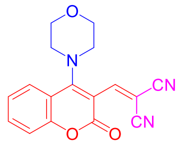

8d

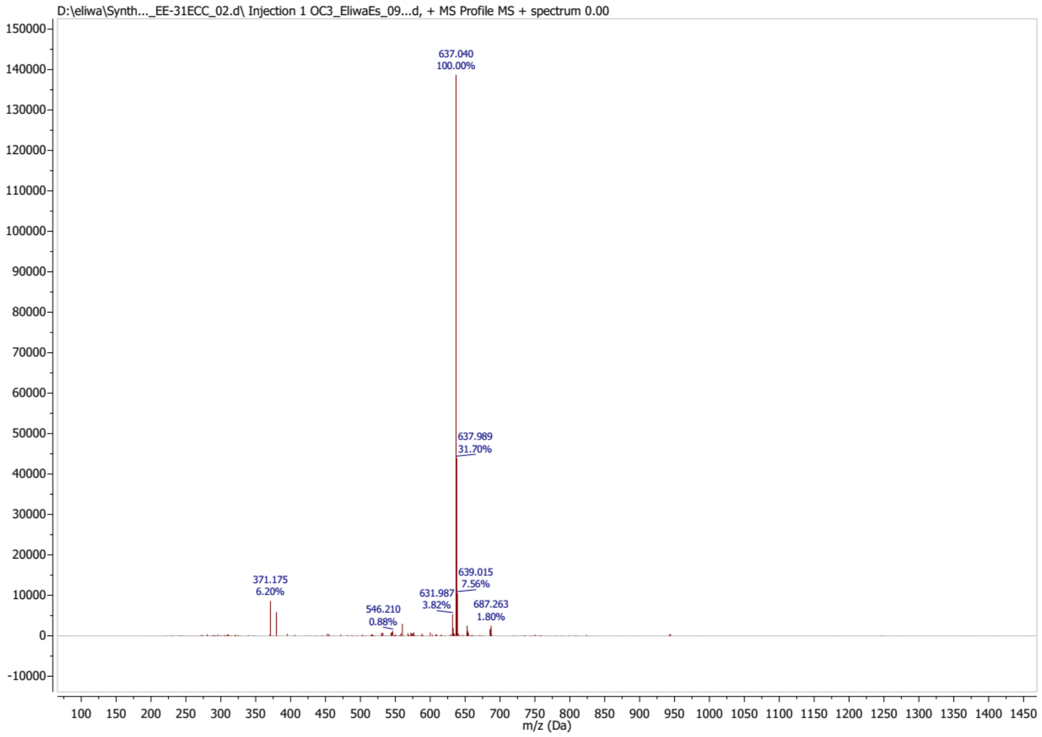

Figure S28. (+)-ESI-MS of **8d**

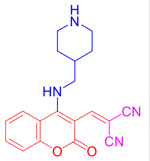

8e

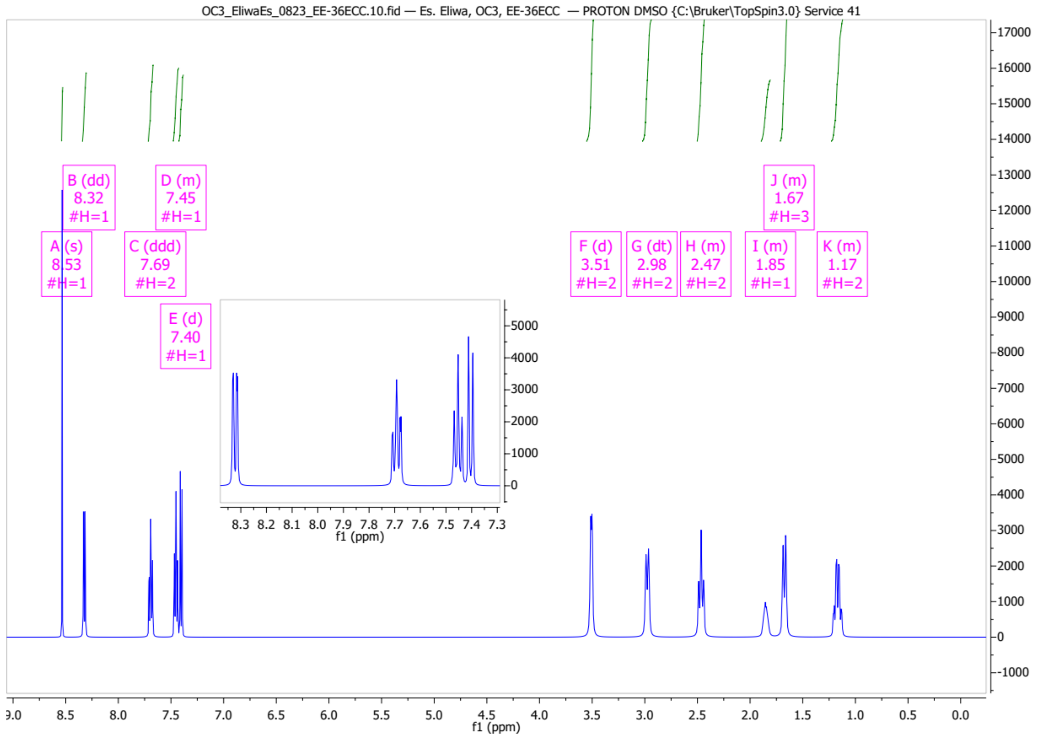

Figure S29. ^{1}H NMR (500 MHz, DMSO-d_6) of **8e**

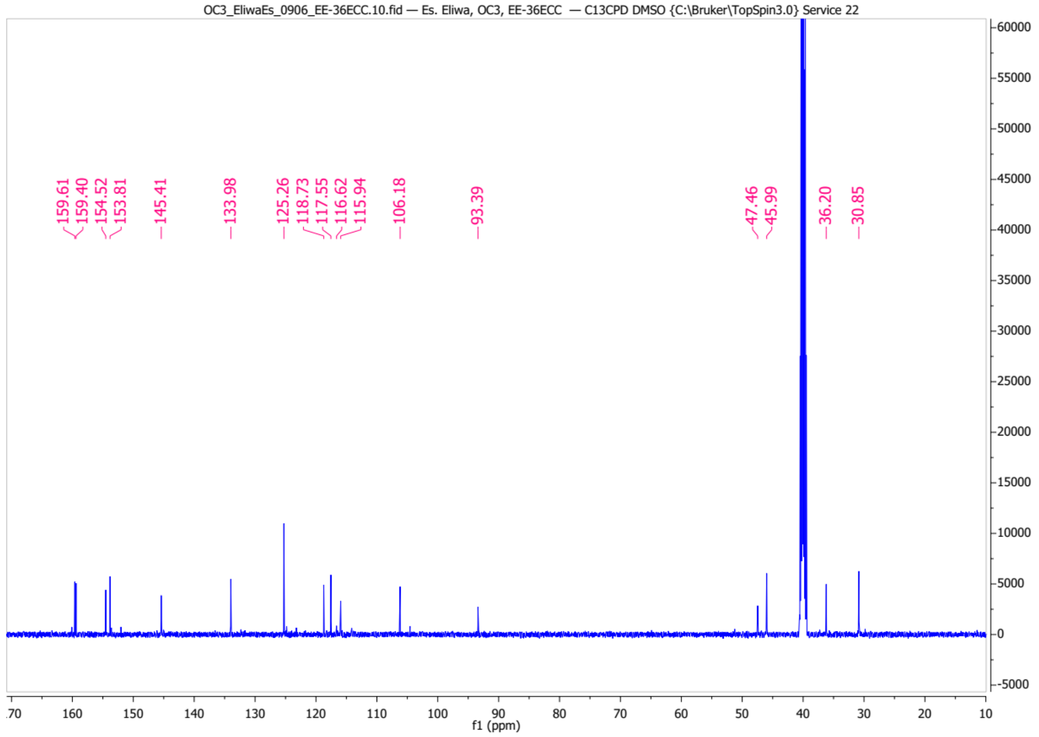

Figure S30. ^{13}C NMR (125 MHz, DMSO-d_6) of **8e**

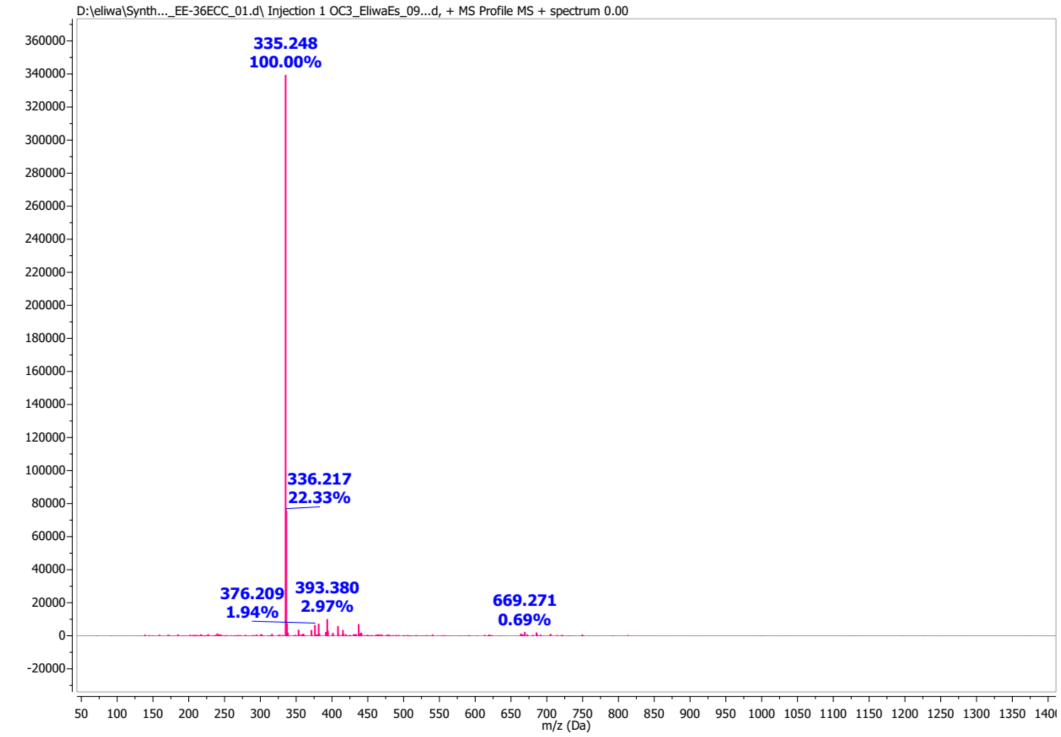

Figure S31. (+)-ESI-MS of **8e**

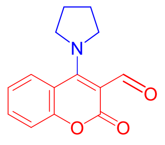

5a

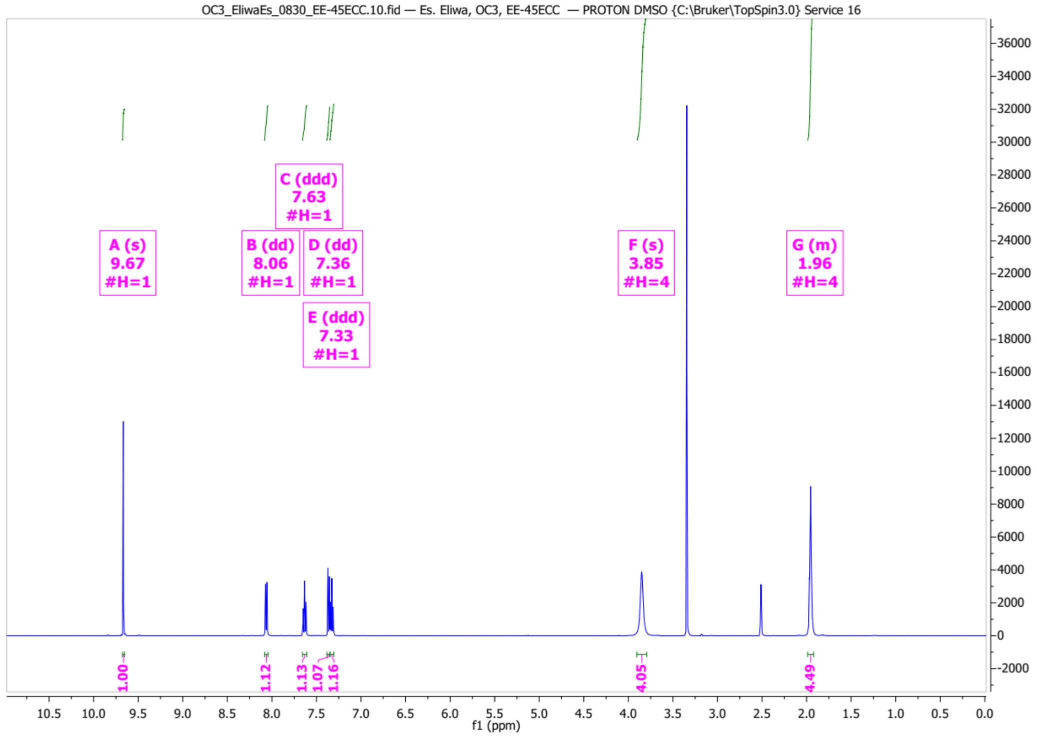

Figure S32. ^{1}H NMR (500 MHz, DMSO-d_6) of **5a**

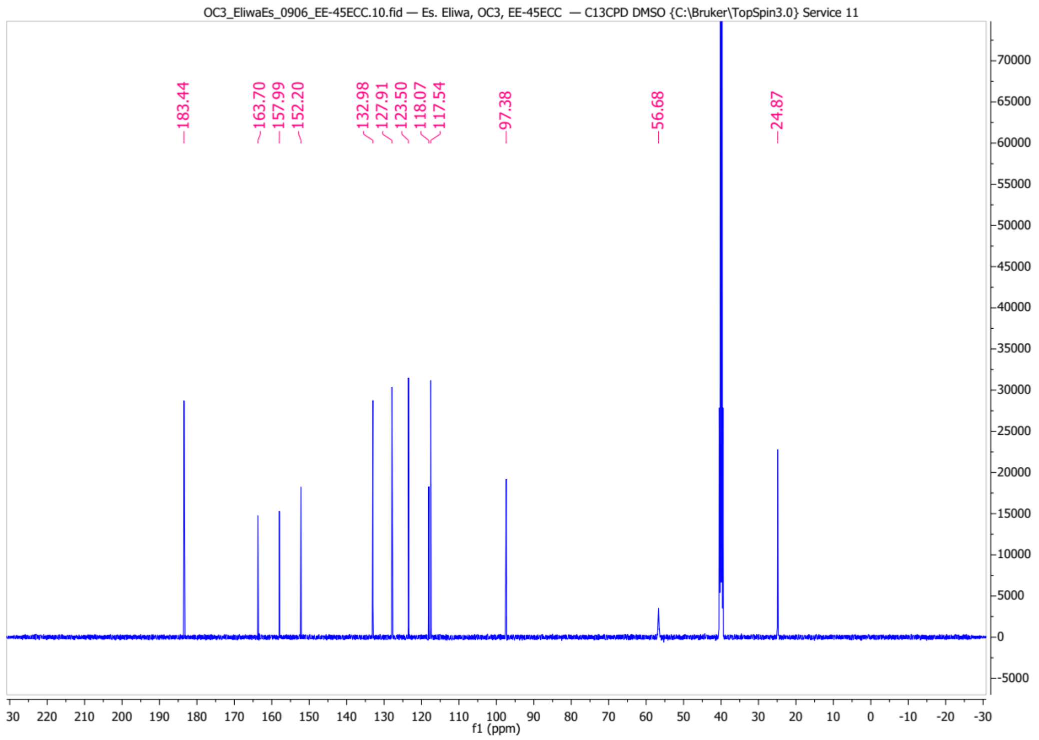

Figure S33. ^{13}C NMR (125 MHz, DMSO-d_6) of **5a**

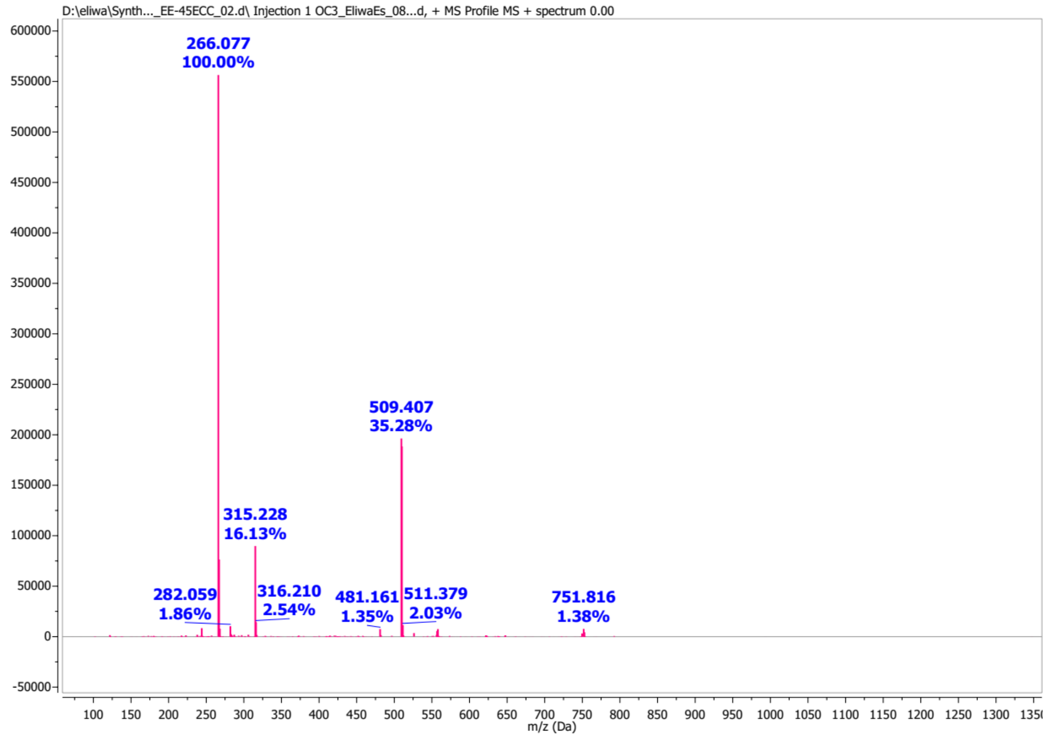

Figure S34. (+)-ESI-MS of **5a**

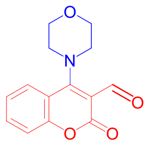

5d

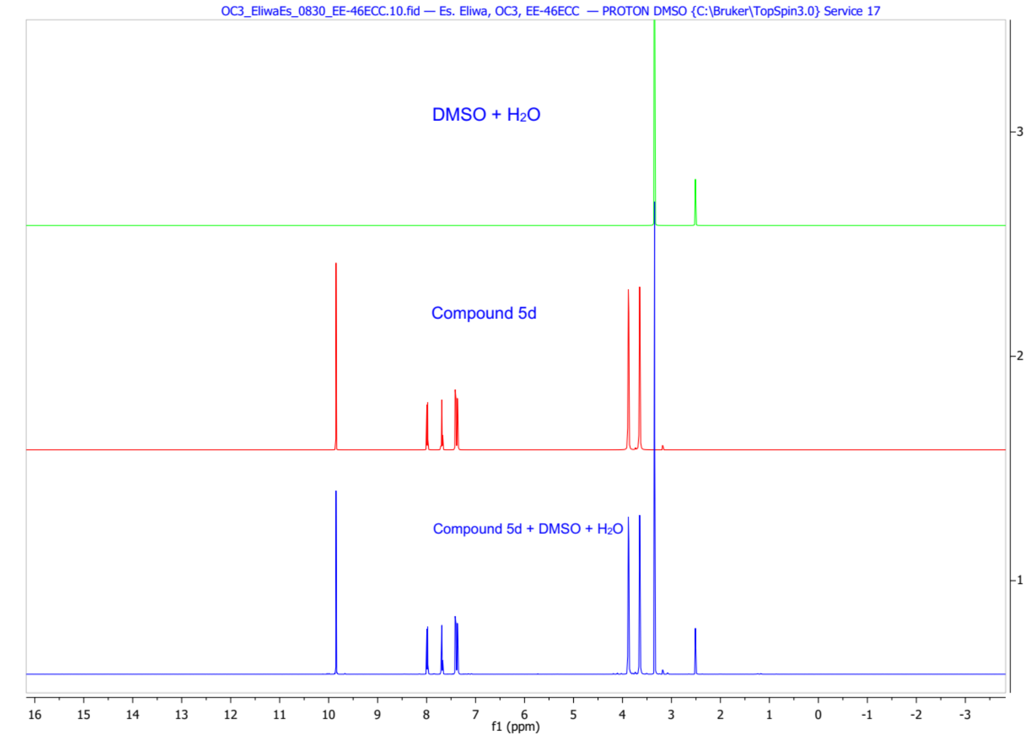

Figure S35A. ^{1}H NMR (500 MHz, DMSO-d_6) of **5d**

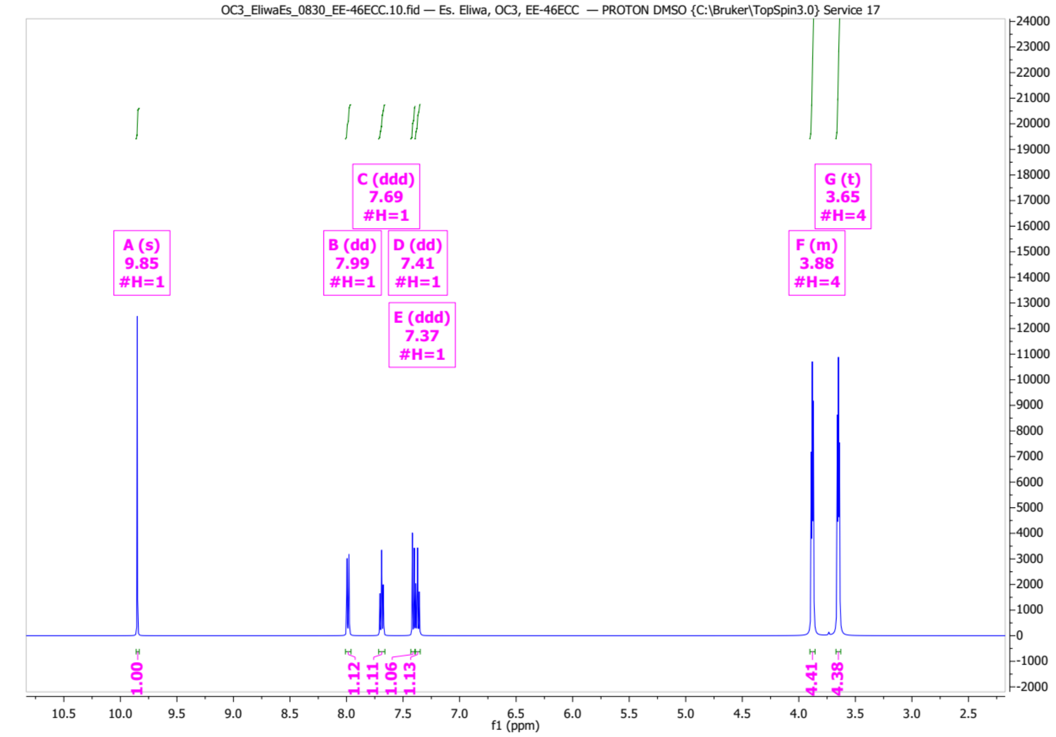

Figure S35B. ^{1}H NMR (500 MHz, DMSO-d_6) of **5d**

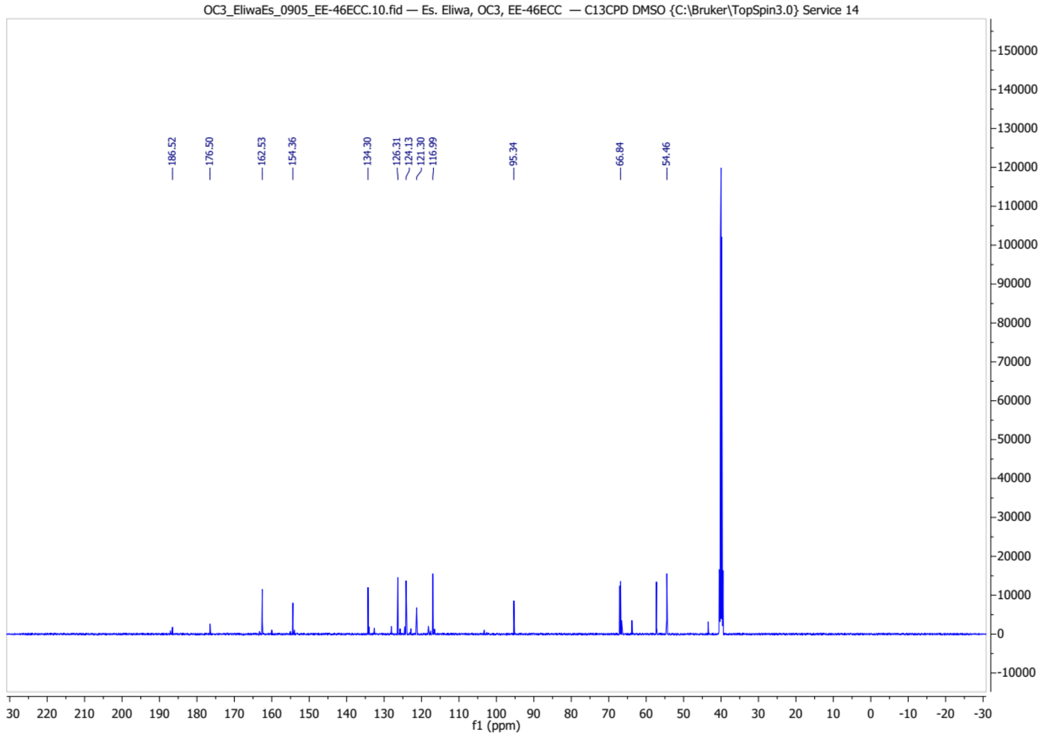

Figure S36. ^{13}C NMR (125 MHz, DMSO-d_6) of **5d**

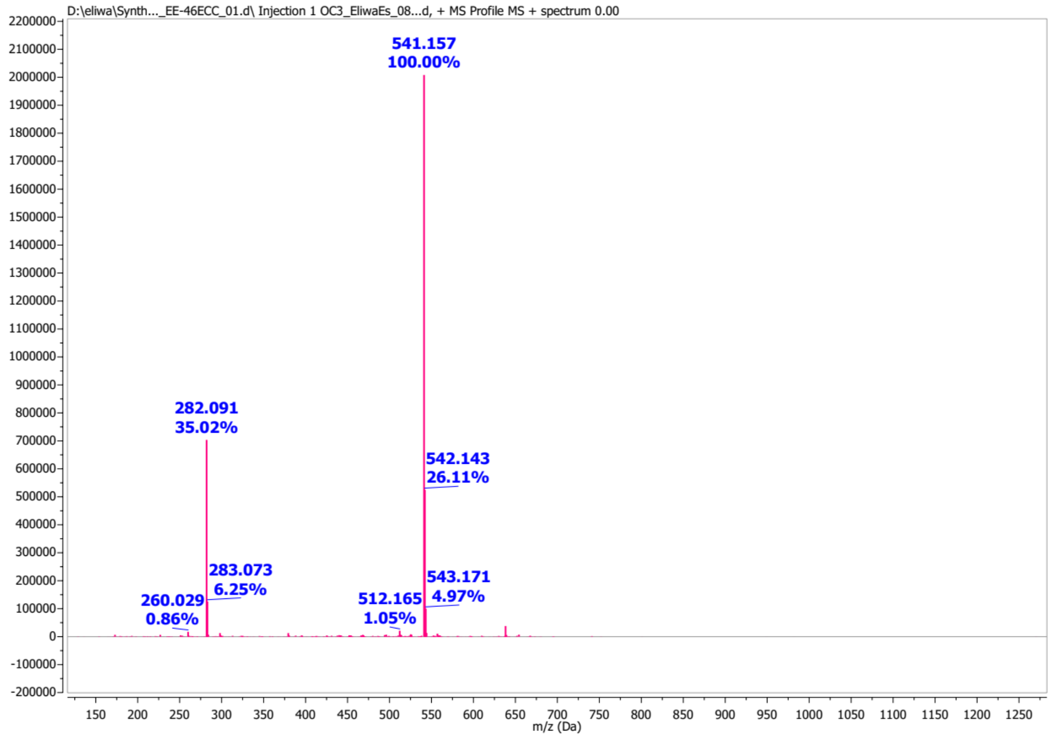

Figure S37. (+)-ESI-MS of **5d**

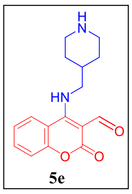

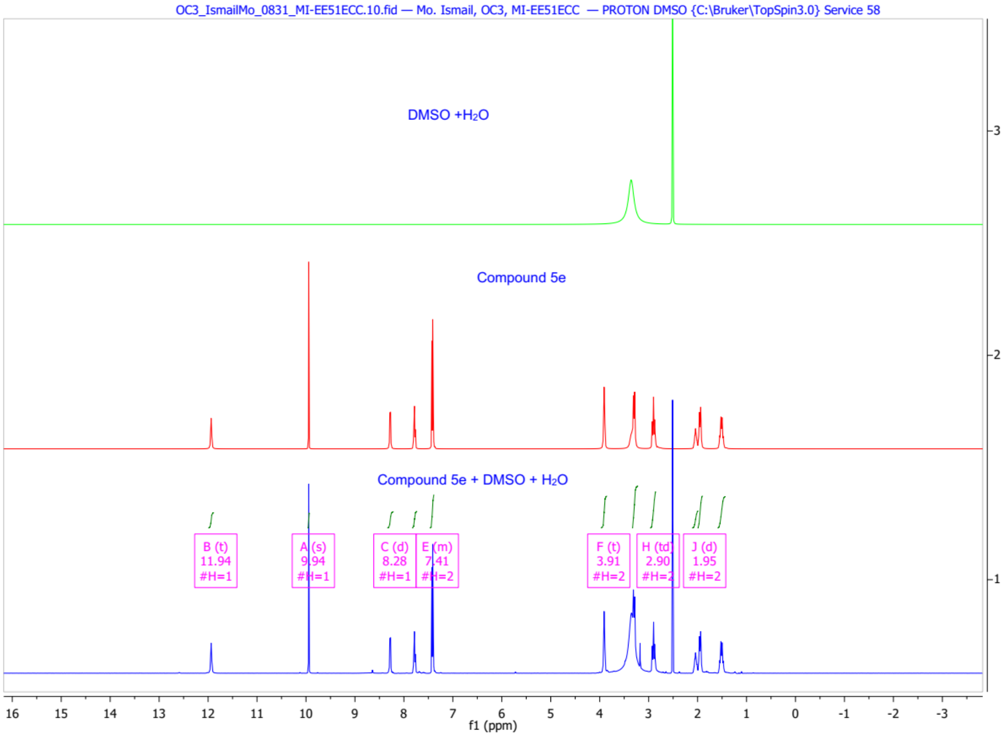

Figure S38A. ^{1}H NMR (500 MHz, DMSO-d_6) of **5e**

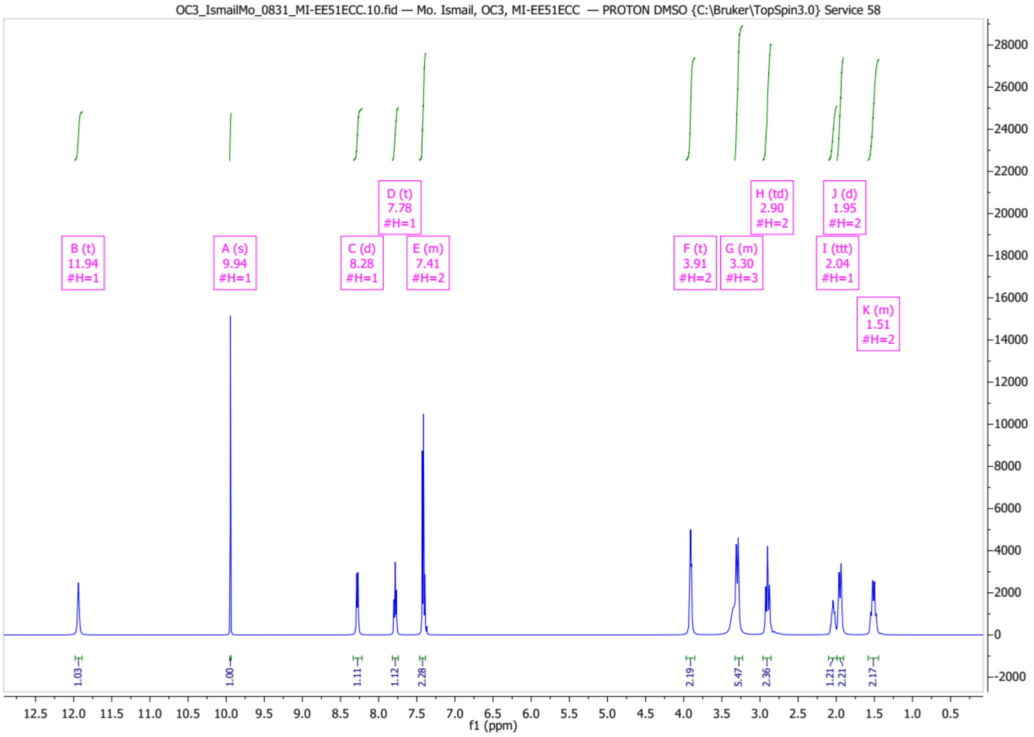

Figure S38B. ^{1}H NMR (500 MHz, DMSO-d_6) of **5e**

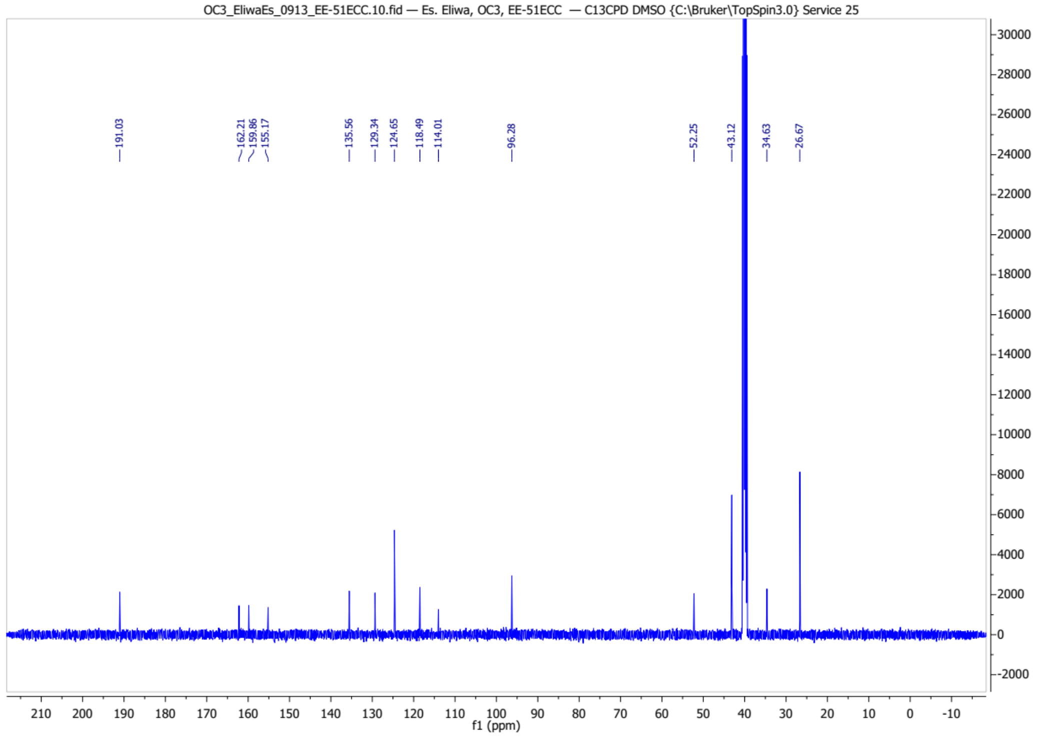

Figure S39. ^{13}C NMR (125 MHz, DMSO-d_6) of **5e**

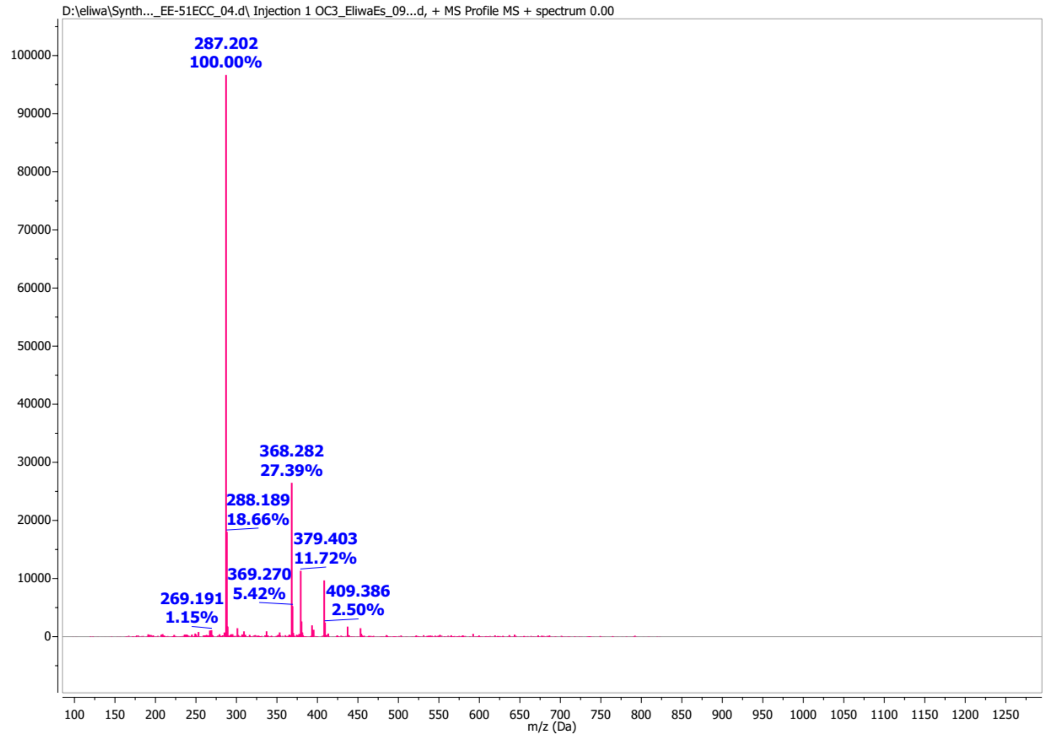

Figure S40. (+)-ESI-MS of **5e**

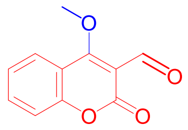

5f

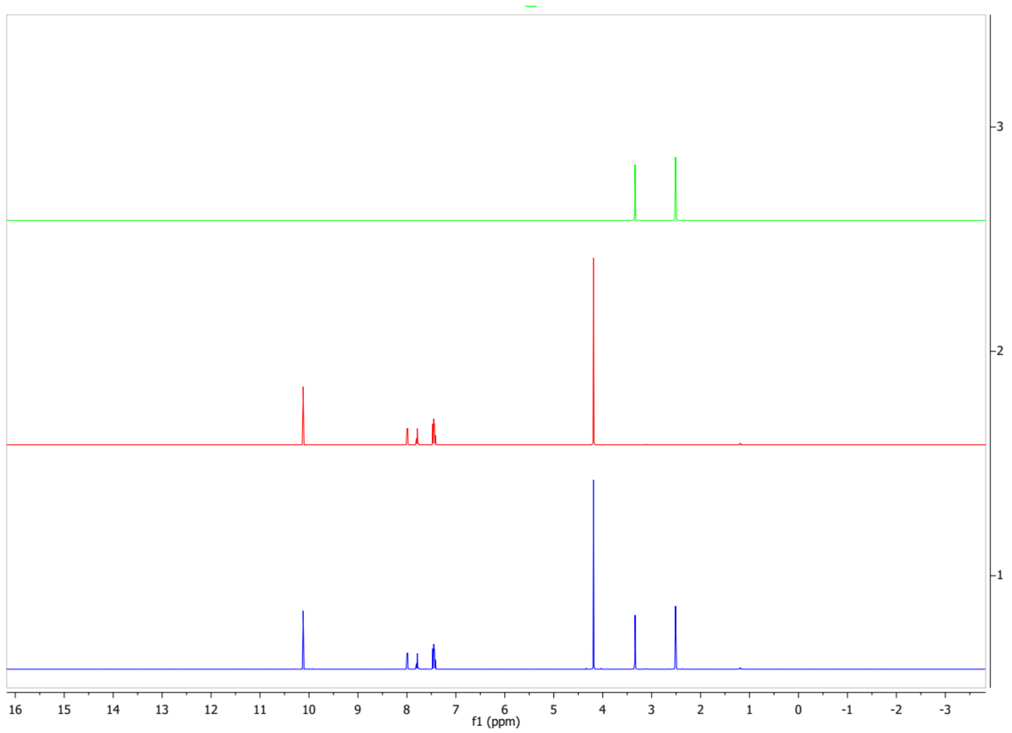

Figure S41A. ^{1}H NMR (500 MHz, DMSO-d_6) of **5f**

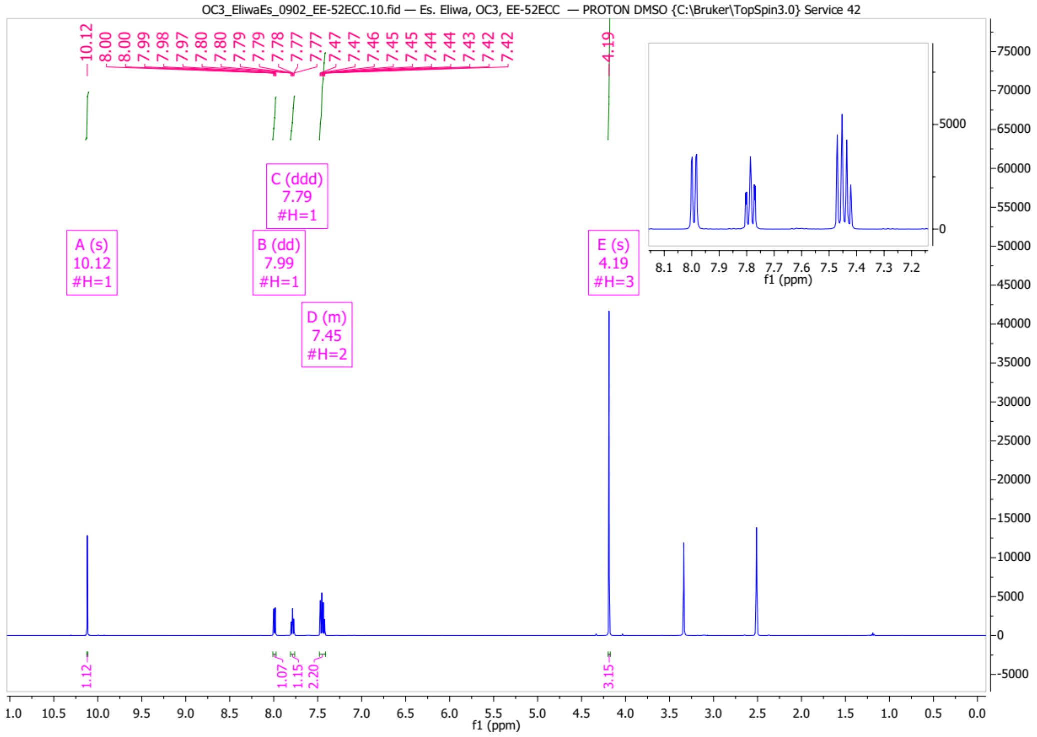

Figure S41B. ^{1}H NMR (500 MHz, DMSO-d_6) of **5f**

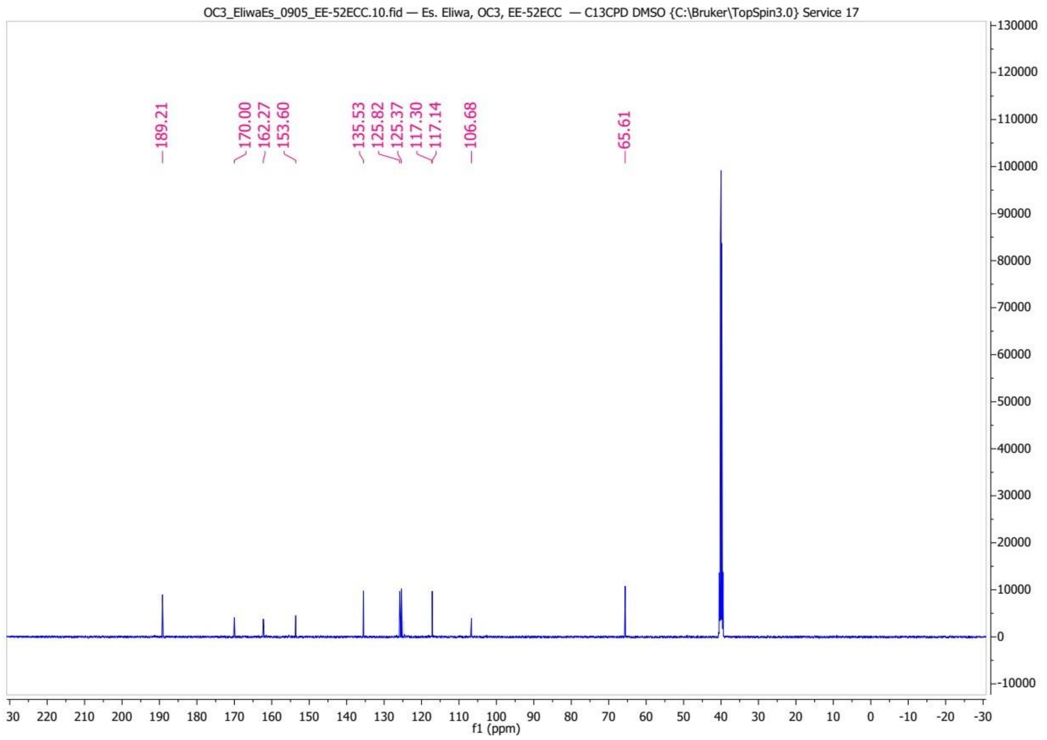

Figure S42. ^{13}C NMR (125 MHz, DMSO-d_6) of **5f**

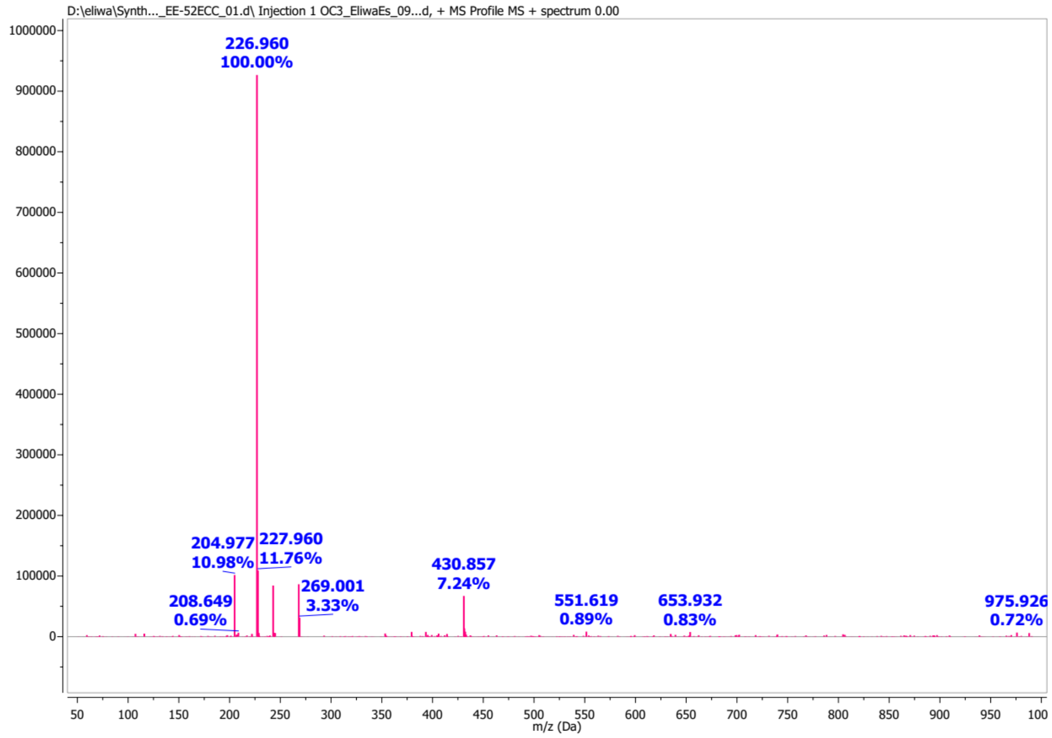

Figure S43. (+)-ESI-MS of **5f**

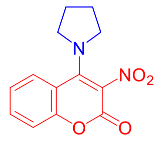

9a

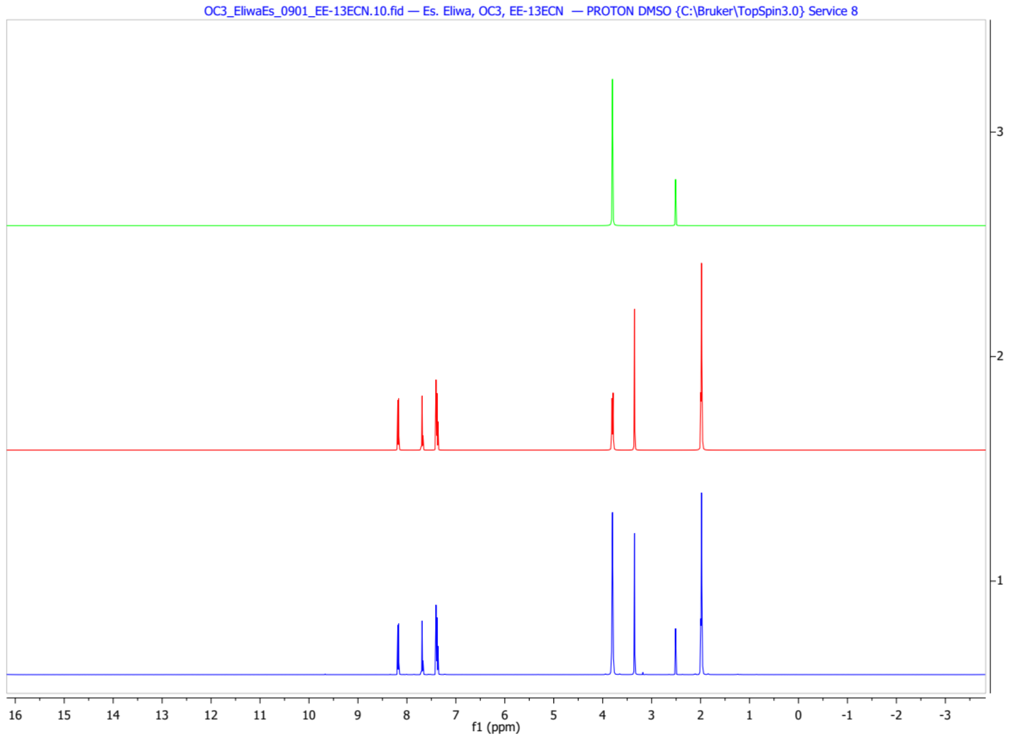

Figure S44A. ^{1}H NMR (500 MHz, DMSO-d_6) of **9a**

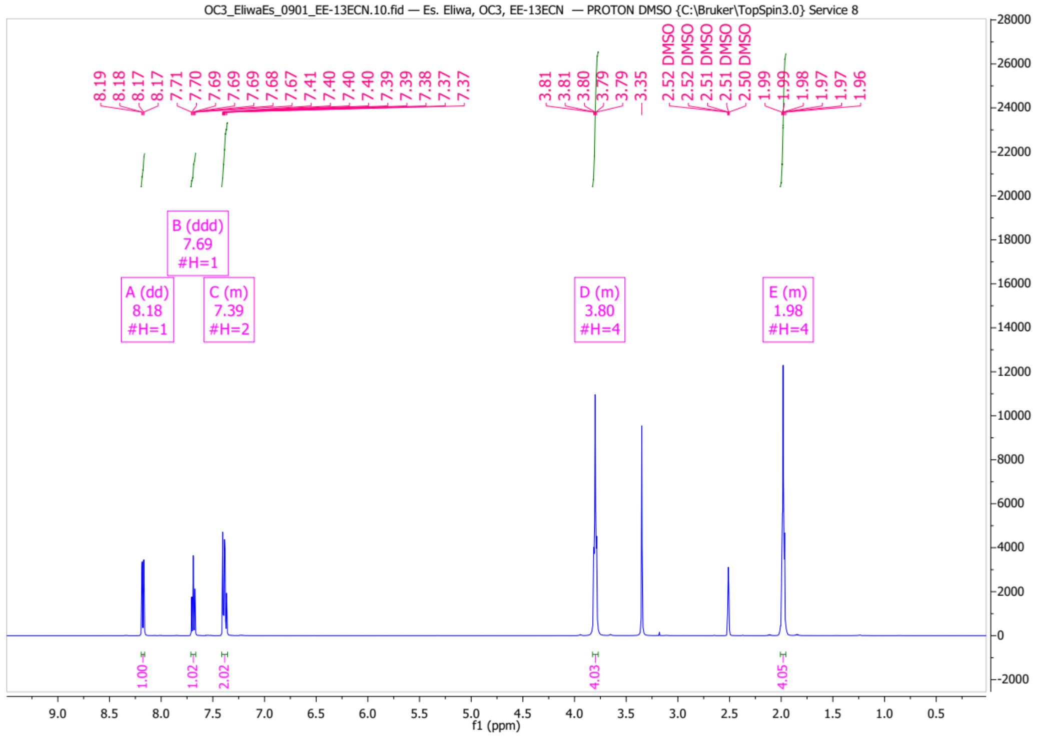

Figure S44B. ^{1}H NMR (500 MHz, DMSO-d_6) of **9a**

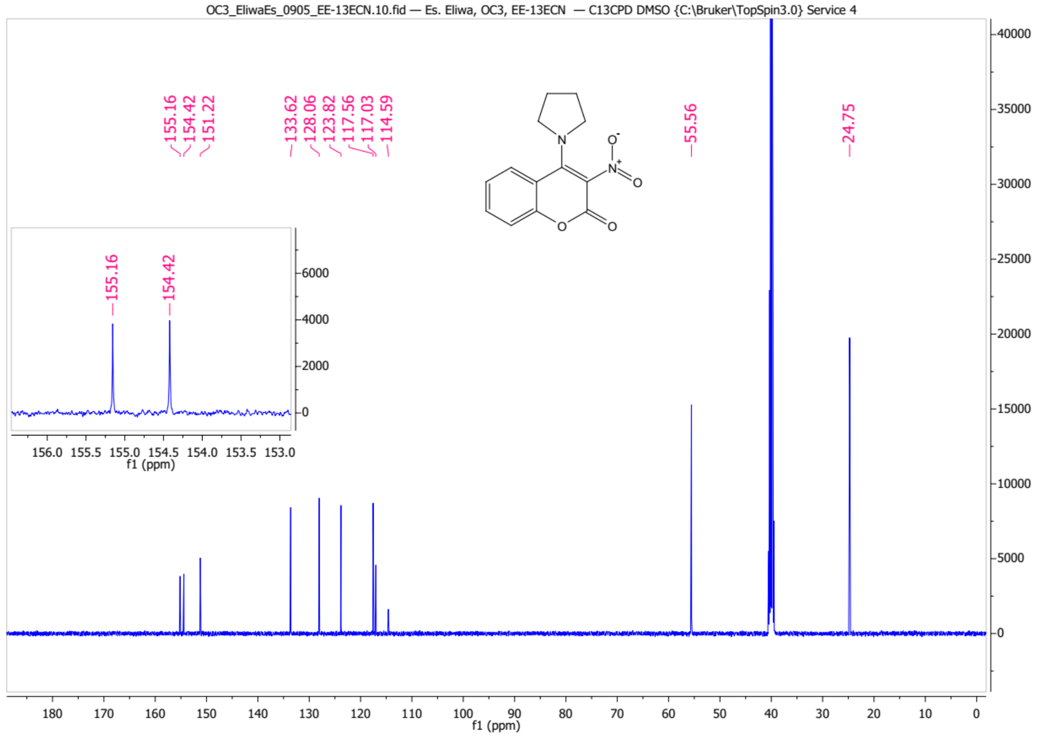

Figure S45. ^{13}C NMR (125 MHz, DMSO-d_6) of **9a**

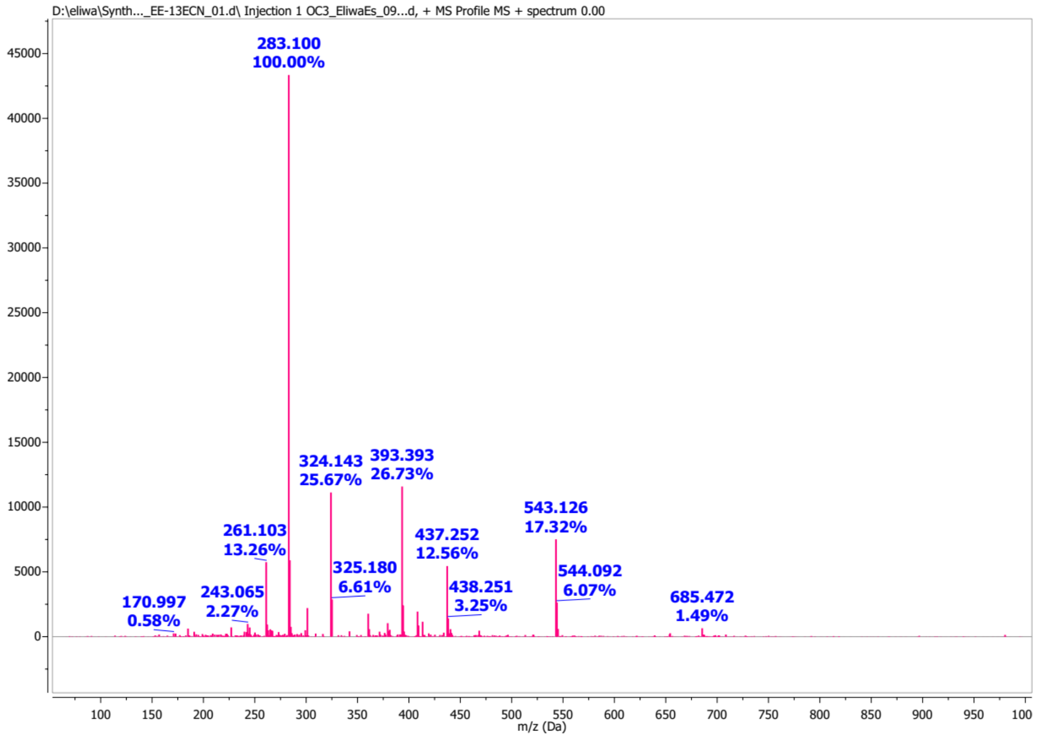

Figure S46. (+)-ESI-MS of **9a**

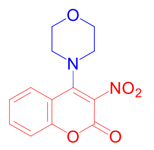

9d

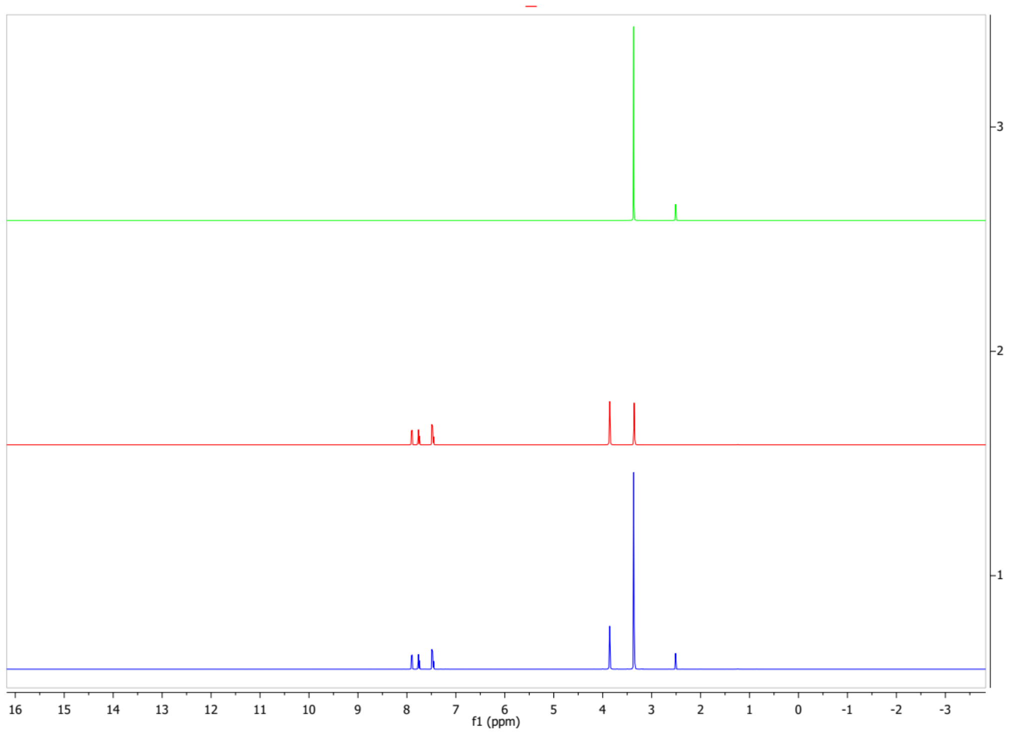

Figure S47A. ^{1}H NMR (500 MHz, DMSO-d_6) of **9d**

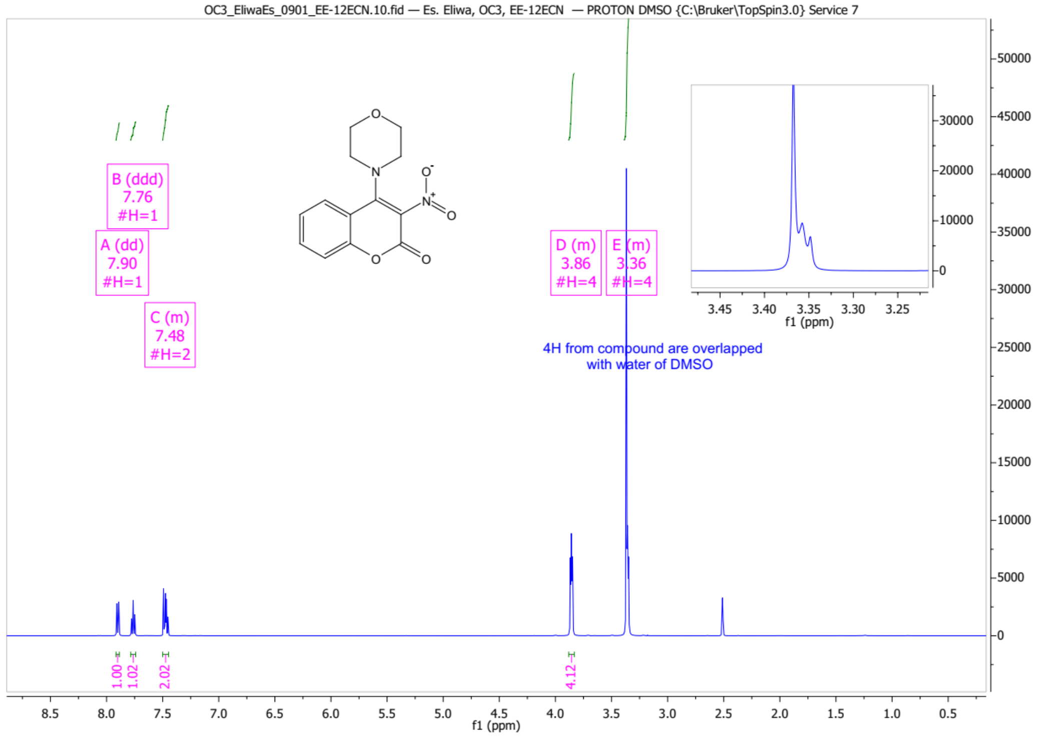

Figure S47B. ^{1}H NMR (500 MHz, DMSO-d_6) of **9d**

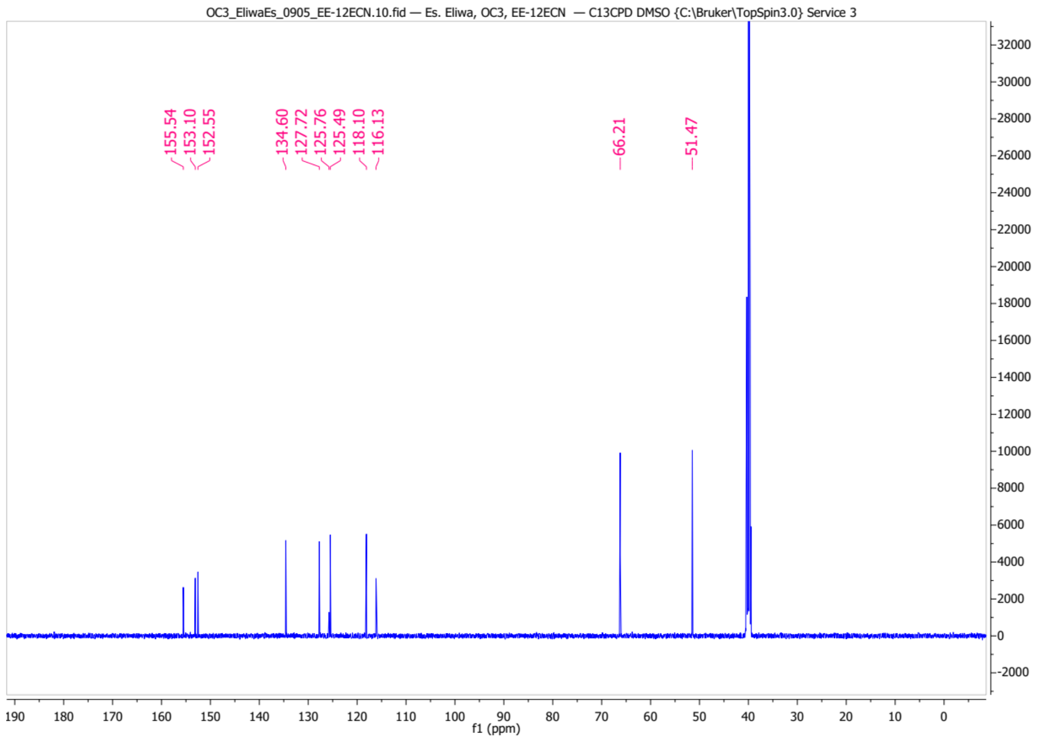

Figure S48. ^{13}C NMR (125 MHz, DMSO-d_6) of **9d**

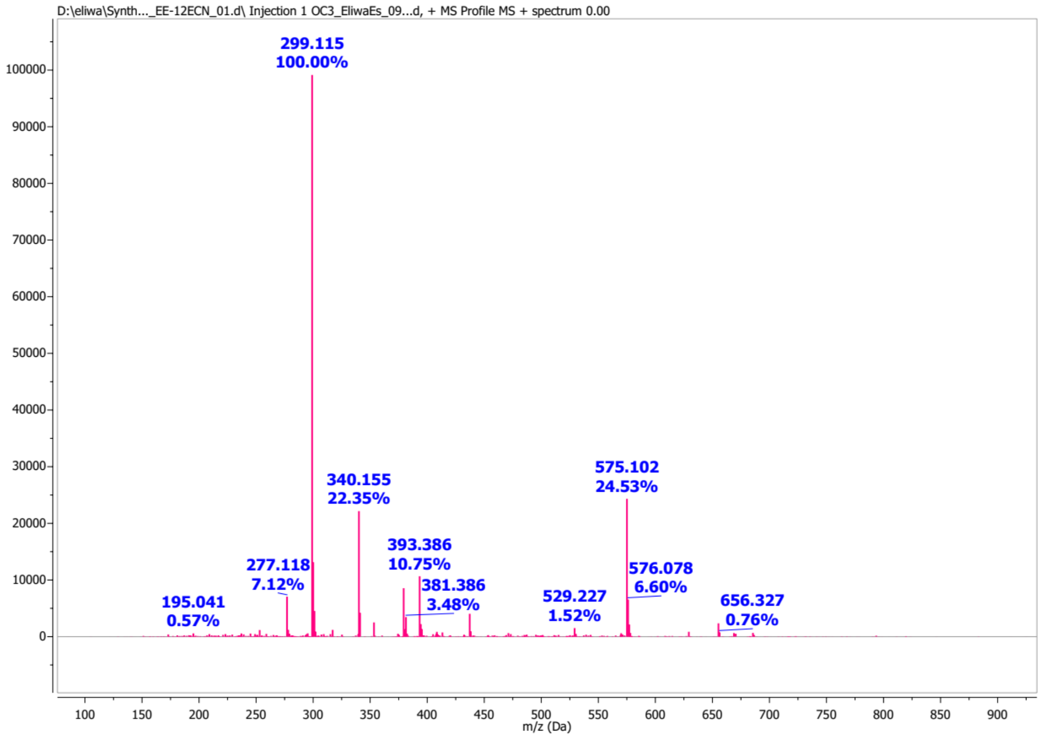

Figure S49. (+)-ESI-MS of **9d**

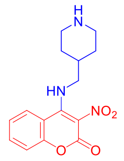

9e

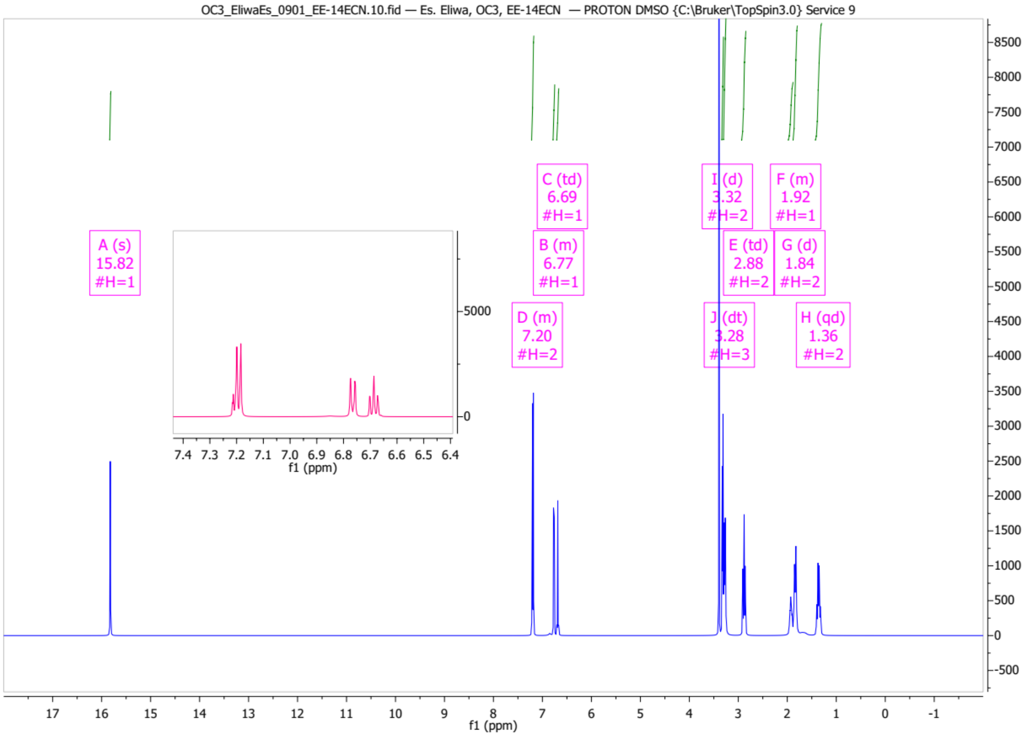

Figure S50. ^{1}H NMR (500 MHz, DMSO-d_6) of **9e**

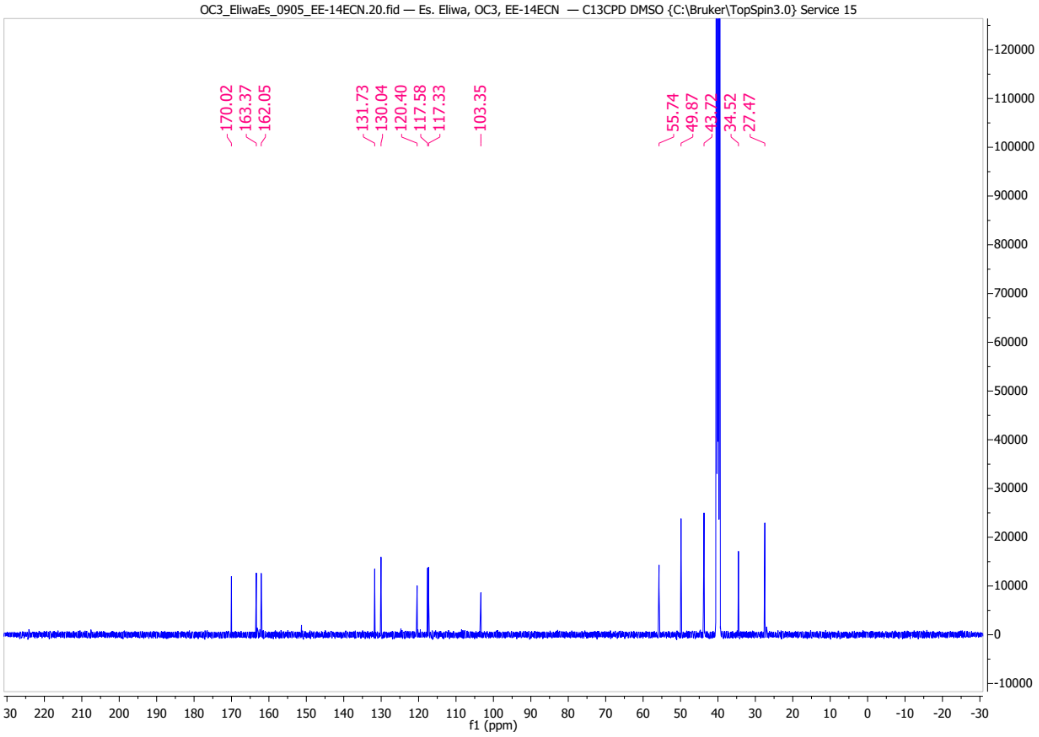

Figure S51. ^{13}C NMR (125 MHz, DMSO-d_6) of **9e**

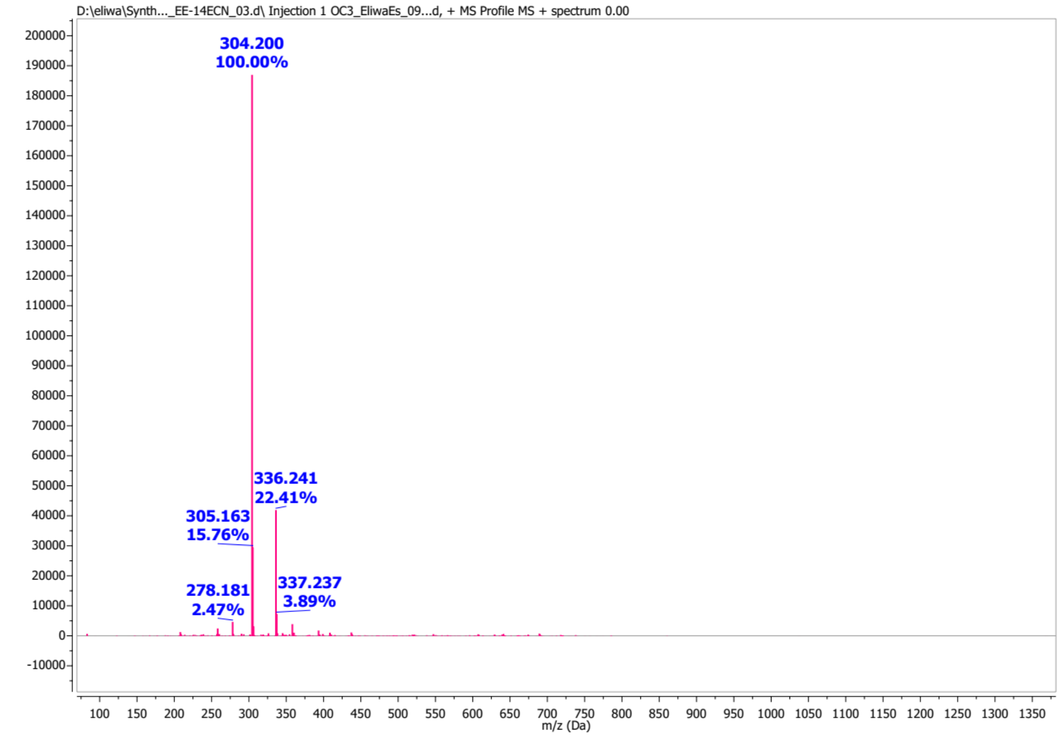

Figure S52. (+)-ESI-MS of **9e**

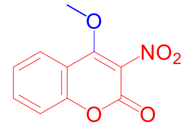

9f

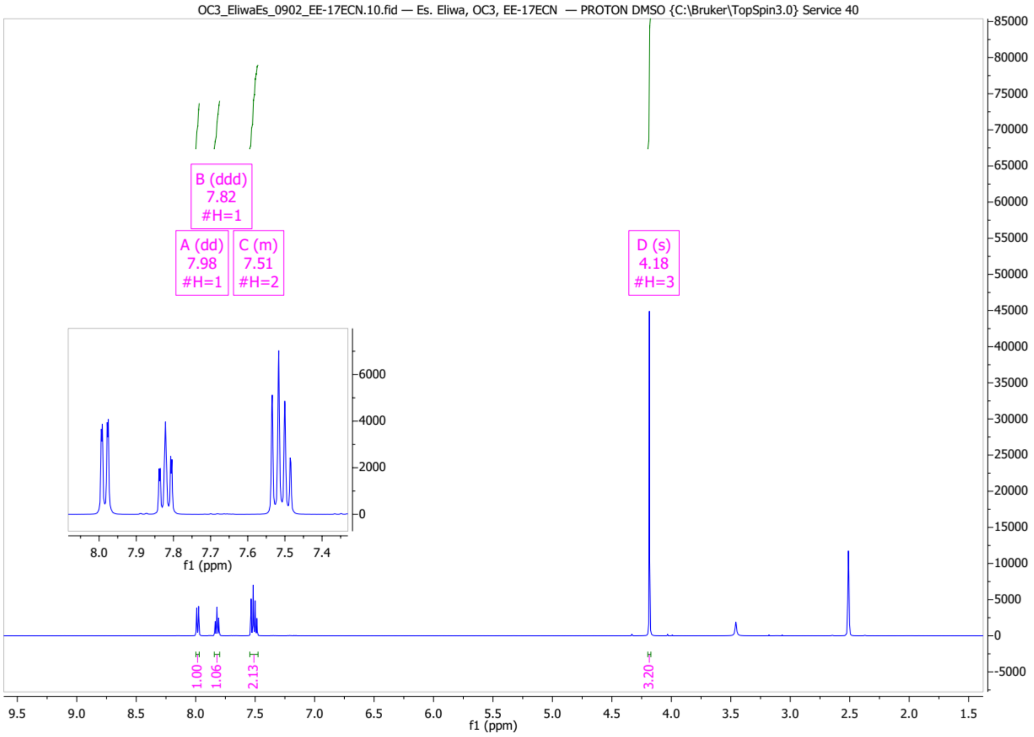

Figure S53. ^{1}H NMR (500 MHz, DMSO-d_6) of **9f**

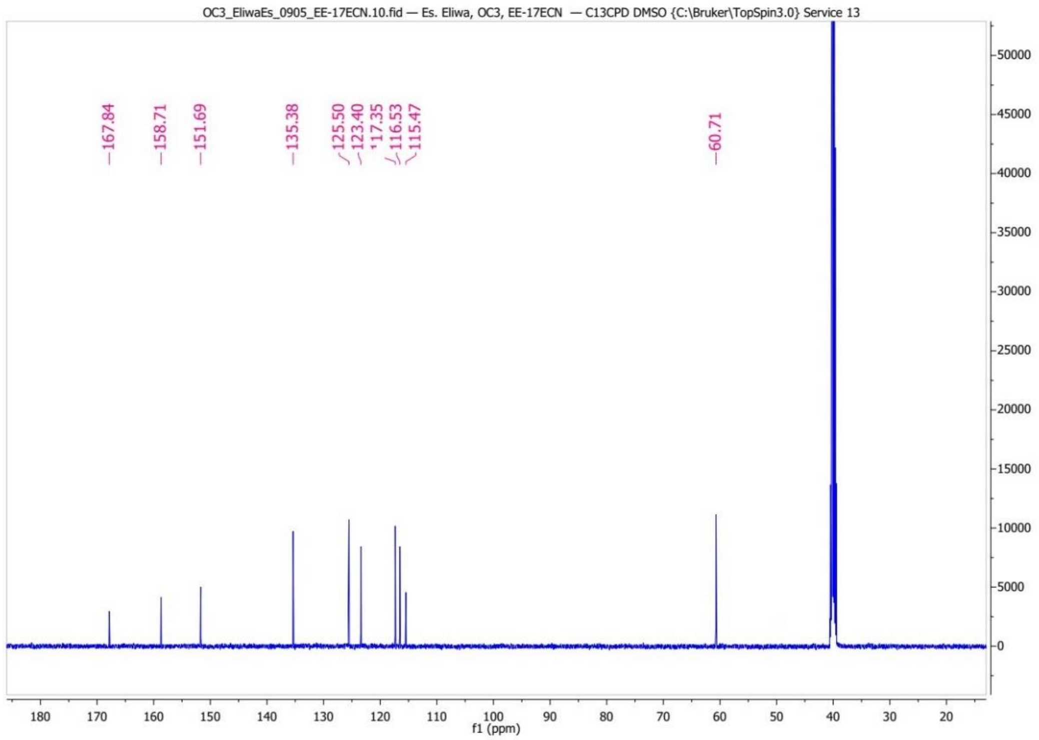

Figure S54. ^{13}C NMR (125 MHz, DMSO-d_6) of **9f**

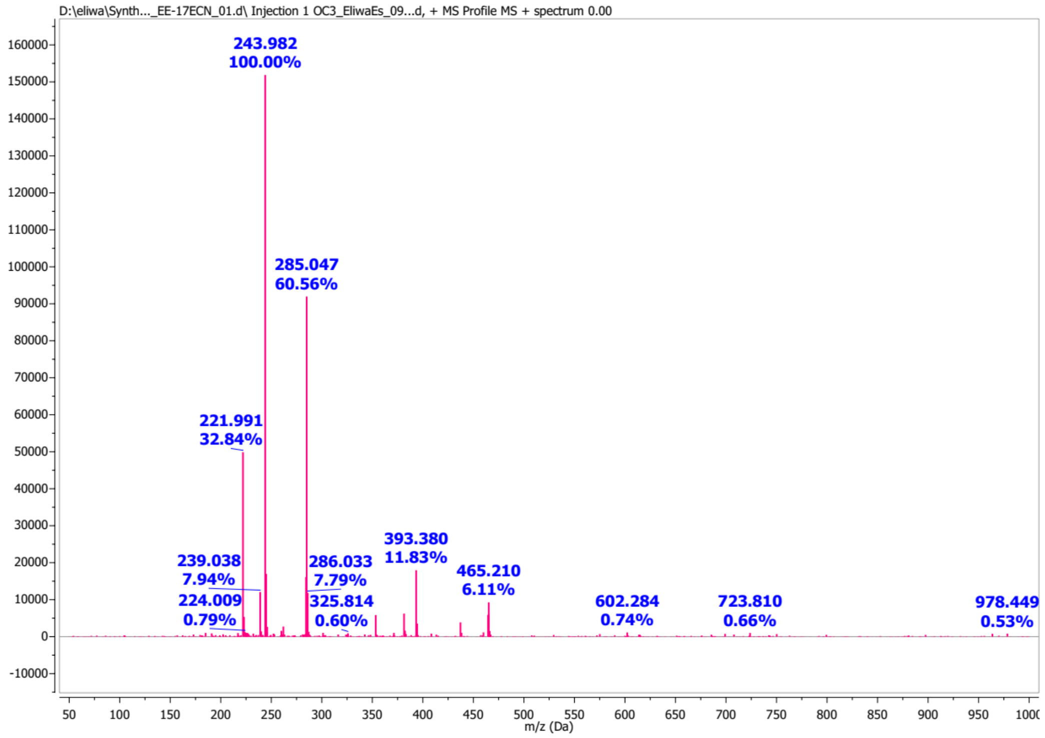

Figure S55. (+)-ESI-MS of **9f**
